# Disability Diagnoses Identified by the American Community Survey 6-Question Sequence

**DOI:** 10.1001/jamahealthforum.2025.6302

**Published:** 2026-01-23

**Authors:** Ari Ne’eman

**Affiliations:** 1 Department of Health Policy and Management, Harvard T.H. Chan School of Public Health, Boston, Massachusetts

## Abstract

**Importance:**

Federal survey data collection identifies people with disabilities predominantly by using a 6-question sequence asking about different functional impairments known as the American Community Survey-6 (ACS-6). However, little is known about the specific diagnoses identified by the ACS-6 or whether they vary across demographic subgroups.

**Objective:**

To characterize the disability diagnoses identified by the ACS-6 and assess to what extent they identify a consistent population across demographic subgroups.

**Design, Setting, and Participants:**

This cross-sectional study among people with disabilities responding to the 2023 or 2024 Survey of Income and Program Participation (SIPP) assessed the prevalence of 36 different diagnosis groupings in the ACS-6 as a whole and within each of the individual questions, as well as how the identified diagnoses varied by age group, race and ethnicity, sex, and educational attainment, with further disaggregation by cognitive disability status. Data were analyzed between August 1 and September 15, 2025.

**Exposure:**

Identification as people with disabilities using the ACS-6.

**Main Outcomes and Measures:**

Diagnoses reported by survey respondents as causing the functional impairments they listed in the SIPP.

**Results:**

A total of 13 341 people with disabilities (52.2% female; mean [SD] age, 53.0 [23.0] years) responding to the SIPP were included. Among people with disabilities aged 22 to 64 years, the most common diagnoses were anxiety or obsessive-compulsive disorders (prevalence, 15.6%; 95% CI, 14.5%-16.9%), depression (15.3%; 95% CI, 14.1%-16.5%), unspecified musculoskeletal issues (13.5%; 95% CI, 12.5%-14.6%), back or spinal problems (11.6%; 95% CI, 10.6%-12.6%), and unspecified neurologic disorders (10.8%; 95% CI, 9.8%-11.8%). The most common disability diagnoses reported by respondents identified by the ACS-6 were different across age groups but similar across demographic groups defined by sex, race and ethnicity, and educational attainment.

**Conclusions and Relevance:**

The results of this cross-sectional study suggest that the ACS-6 identifies a similar population across demographic subgroups not characterized by age but highlight substantial heterogeneity in the population of people with disabilities within these subgroups and across age groups. Contemporary debates regarding future revisions to disability data collection in federal population surveys should address the ability to account for this heterogeneity in survey design.

## Key Points

**Question:**

What diagnoses are identified by the American Community Survey-6 (ACS-6) used to locate people with disabilities in federal population surveys?

**Findings:**

In this cross-sectional study of 13 341 people with disabilities in the Survey of Income and Program Participation, diagnosis groupings identified by the ACS-6 varied within each question and across age groups but were consistent across race and ethnicity, sex, and education subgroups.

**Meaning:**

These findings suggest research involving people with disabilities should determine subgroups appropriate to the research question rather than approach disability as a binary category and should account for disability heterogeneity alongside attempts to avoid underidentification of people with disabilities.

## Teaser

This cross-sectional study assesses the diagnoses identified by the American Community Survey-6 disability sequence among people with disabilities reporting functional impairments.

## Introduction

Since 2008, the American Community Survey (ACS), Current Population Survey (CPS), and multiple other federal population surveys have asked respondents a series of 6 questions regarding disability status, each inquiring about a different type of functional impairment. These questions permit researchers to identify people with disabilities in survey data, even as respondents may carry different understandings of what the term *disability* implies. This 6-question sequence (ACS-6) asks about respondent impairments in hearing, seeing, cognition, mobility, self-care, and independent living (eTable 1 in Supplement 1 reports question wording). The ACS-6 has been endorsed by the US Department of Health and Human Services as a uniform data collection standard for inclusion in population surveys^1^ and has also been used to study a broad array of topics affecting people with disabilities.^2,3,4,5,6,7,8,9,10,11,12^ With the recent recognition of people with disabilities as a population with health disparities by the National Institutes of Health, identifying disability in survey data is increasingly policy relevant.^13^

Prior research^5,14,15,16,17,18,19^ has found limitations to the ACS-6, focusing primarily on whom it does not identify. However, despite the prominence of the ACS-6 in disability measurement, relatively little research has examined whom these commonly used questions do identify and to what extent they capture a consistent population across demographic subgroups. In the absence of this information, data users often attribute the impairments reported by respondents and subsequent identification of populations with disabilities by the ACS-6 to specific diagnoses without evidence. For instance, prior work^7,9^ has used the population with cognitive disability identified by the ACS-6 to study employment outcomes for people with intellectual and developmental disabilities (I/DD). Similarly, after researchers documented an increase in cognitive disability diagnoses, defined as serious difficulty concentrating, remembering, or making decisions because of a physical, mental, or emotional condition, during the COVID-19 public health emergency,^8,20,21^ some observers attributed this to long COVID.^22,23,24^ In the absence of research linking ACS-6 questions to specific diagnoses, it is difficult to evaluate the appropriateness of these and other usages of the ACS-6.

A related concern is the degree of consistency across demographic groups in how the ACS-6 is interpreted. If questions are interpreted differently by different respondents, this creates a risk of across-group measurement bias if propensity to underreport is correlated with demographics. The latter risk is particularly important for disability research. If the ACS-6 does not identify a consistent population with disabilities across specific demographic groups, stratified analyses, such as analyses of disparities by race or ethnicity, age, or educational attainment, will be irreparably biased.

The Survey of Income and Program Participation (SIPP), which includes the ACS-6, recently began collecting information on the specific conditions that caused respondents’ functional impairments, clarifying prior problems in question wording in 2023.^25^ Using SIPP data, this study reports the first estimates to our knowledge from a federal population survey of what diagnoses are identified by the ACS-6, analyzing the extent to which they identify a consistent population across demographic subgroups.

## Methods

### Data

The SIPP is a nationally representative longitudinal survey of the US civilian noninstitutionalized population that collects a broad array of information on income, employment, household composition, demographic characteristics, and public program participation.^26^ Weighted response rates were 46.50 for 2023 and 46.40 for 2024.^27^ In this cross-sectional study, we included all respondents who reported an ACS-6 functional impairment in the 2023 SIPP and all respondents who reported an ACS-6 impairment in the 2024 SIPP who were not previously surveyed in 2023. To ensure results were not skewed from using data from 2 distinct years, we replicated our primary analyses using data from only 2024. This study was deemed not human participant research using the Harvard Longwood Campus Institutional Review Board Decision Tool and therefore exempt from institutional review board review. This cross-sectional study used the Strengthening the Reporting of Observational Studies in Epidemiology (STROBE) reporting guideline.

### Collection Method

Respondents were asked the ACS-6 and other disability screening questions in personal interviews, typically conducted in person.^28^ Respondents who provided an affirmative response to a disability question, asking whether they had challenges with certain activities, were asked to report the condition that caused the difficulty with the activities. Respondents could choose from an answer list of more than 400 conditions. Interviewers could type the first 3 letters of the diagnosis shared by the respondent, and all conditions beginning with those letters would appear for selection. Respondents and interviewers were able to report up to 3 conditions. Answers were recoded by survey administrators, including into the scheme of 36 diagnostic categories used in this study.

### Statistical Analysis

Data were analyzed between August 1 and September 15, 2025. Due to the increase in cognitive disability rates during the COVID-19 public health emergency,^8,20,21^ we report most results separately for persons with and without cognitive disabilities to examine unique dynamics associated with cognitive disability and maximize generalizability across time for other people with disabilities.

To assess the extent to which persons identified by the ACS-6 within the SIPP are comparable with those identified by the same questions in other surveys, the most common combinations of ACS-6 functional impairments reported by prevalence were determined for the SIPP analytic sample from 2023 to 2024 used in our study, the CPS 2023 Annual Social and Economic Supplement (ASEC), and the 2023 ACS. Then, for the 15 most common combinations in the SIPP, pairwise Spearman correlation coefficients were calculated to assess the similarity of the rank orders and Pearson correlation coefficients were calculated to asses the similarity in the proportion of each diagnosis captured by the ACS-6 between the SIPP and each other sample. We conducted these comparisons both overall and disaggregated by cognitive disability status. We did not expect precise alignment across surveys, but correlation offers insight into generalizability.^29^

We then report the most common disability diagnoses overall and within mutually exclusive cognitive and noncognitive categories. To evaluate the degree to which the ACS-6 identifies common diagnoses across demographic subgroups, we calculated Spearman and Pearson correlation coefficients within the cognitive and noncognitive categories comparing different age subgroups, defined as children and young adults (aged 5 to 21 years; selected to correspond to the Individuals With Disabilities Education Act authorization for certain children with disabilities to stay in public school until age 22 years), working-age adults (aged 22 to 64 years), and older adults (aged 65 years or older); sex groups (male and female); racial and ethnic groups (Asian, Black, Hispanic, non-Hispanic White, and other [no further breakdown is available]); and educational attainment groups (no high school diploma, high school diploma, some college, and Bachelor’s degree or higher). Race and ethnicity were self-reported. There were 2 variables: one for Asian, Black, White, and other (race), and another for Hispanic status. We created a single 5-level variable from the 2 variables. We report correlations between each of these subgroups for both the cognitive and noncognitive categories. Because rare diagnoses clustered at the bottom of the distribution for all groups may have artificially inflated the correlation coefficients, we conducted a robustness check limited to the 15 most frequent diagnoses.

We also report the prevalence of each of the 36 diagnostic subgroupings in the overall ACS-6 population and within each of the 6 functional impairment categories in the ACS-6. Unlike the analyses disaggregated by cognitive disability status, these functional impairment categories are overlapping and not mutually exclusive, permitting a respondent who reported multiple functional impairments to contribute to reported prevalence within multiple impairment categories. We report this both overall and by demographic subgroups. Because the cognitive disability variable has been used with the intention of studying the I/DD service system,^7,9^ we report the prevalence of intellectual disability and autism diagnoses within the cognitive disability category. Because the increase in disability rates has been attributed by many to long COVID, we also report rates of reporting COVID-19 in the ACS-6 in general and within the cognitive disability variable in particular.^22,23,24^ To assess generalizability across datasets, we present cognitive disability rates normalized to 2019 levels from 2018 to 2023 for the SIPP, CPS, and ACS. Finally, we also report the percentage of respondents who reported no diagnosis by age category.

All SIPP prevalence estimates were weighted using the person-level annual weight from the month-12 observation for their respective year. We calculated 95 CIs using a logit transformation to ensure that all estimates were bound between 0 and 100. Analyses used R version 4.3.1 (R Foundation for Statistical Computing). The significance of correlations was calculated using 2-sided *P* values, with significance at the *P* < .001 level.

## Results

A total of 13 341 people with disabilities (52.2% female; mean [SD] age, 53.0 [23.0] years) responding to the SIPP were included. Disability subgroups were relatively constant across the SIPP, ASEC, and ACS. The correlation coefficients were high, indicating they identified a very similar diagnosis population. Spearman rank correlation was ρ = 0.857 (*P* < .001) when comparing the SIPP with the ASEC and ρ = 0.882 (*P* < .001) when comparing the SIPP with the ACS. Pearson correlation was *r* = 0.845 when comparing the SIPP with the ASEC and *r* = 0.948 when comparing the SIPP with the ACS. Results were similar across cognitive disability status (eTables 2 to 5 in Supplement 1).

Table 1 reports Spearman and Pearson correlation coefficients, showing the degree of correlation in rank order and proportions of diagnoses captured by the ACS-6 across different demographic subgroups. Diagnoses were strongly correlated across demographic subgroups defined by sex, race and ethnicity, and educational attainment but were only weakly or inversely correlated when comparing diagnoses identified across age groups, indicating that the ACS-6 identifies different disability populations depending on age. Results were substantively similar when limited to the 15 most frequent diagnoses overall (eTable 6 in Supplement 1). eTable 7 in Supplement 1 lists the 5 most common disability diagnoses overall and for persons with and without cognitive disabilities. eTable 8 in Supplement 1 reports prevalence estimates overall and disaggregated by cognitive disability status for all diagnoses.

**Table 1.** Correlation of Disability Diagnosis Prevalence Across Demographic Groups by Cognitive Disability Status

Demographic characteristic	Cognitive		Noncognitive	
	Spearman^a^	Pearson^b^	Spearman^a^	Pearson^b^
Age group, y				
5-21 or 22-64	0.580^c^	0.664^c^	0.386^d^	0.325
22-64 or ≥65	0.299	0.257	0.798^c^	0.913^c^
5-21 or ≥65	−0.266	−0.180	0.103	0.162
Sex				
Male or female	0.851^c^	0.865^c^	0.855^c^	0.901^c^
Race and ethnicity^e^				
Asian or Hispanic	0.773^c^	0.887^c^	0.884^c^	0.960^c^
Asian or other	0.634^c^	0.835^c^	0.770^c^	0.918^c^
Black or Asian	0.737^c^	0.825^c^	0.881^c^	0.924^c^
Black or Hispanic	0.871^c^	0.933^c^	0.850^c^	0.913^c^
Black or other	0.734^c^	0.843^c^	0.835^c^	0.925^c^
Hispanic or other	0.813^c^	0.908^c^	0.776^c^	0.877^c^
White or Asian	0.817^c^	0.891^c^	0.888^c^	0.944^c^
White or Black	0.891^c^	0.899^c^	0.867^c^	0.916^c^
White or Hispanic	0.901^c^	0.966^c^	0.848^c^	0.911^c^
White or other	0.813^c^	0.937^c^	0.827^c^	0.926^c^
Educational level^f^				
No HS or HS diploma	0.882^c^	0.865^c^	0.938^c^	0.935^c^
No HS or some college	0.791^c^	0.794^c^	0.927^c^	0.932^c^
No HS or BA or higher	0.709^c^	0.617^c^	0.881^c^	0.858^c^
HS diploma or some college	0.856^c^	0.957^c^	0.937^c^	0.987^c^
HS diploma or BA or higher	0.801^c^	0.860^c^	0.936^c^	0.965^c^
Some college or BA or higher	0.917^c^	0.934^c^	0.929^c^	0.960^c^

Abbreviations: BA, bachelor’s degree; HS, high school.

^a^ Spearman correlation coefficients show the correlation in the rank order of diagnoses across populations of people with disabilities under comparison.

^b^ Pearson correlation coefficients show the correlation in the share of each diagnosis across populations of people with disabilities under comparison.

^c^ *P* < .001.

^d^ *P* < .05.

^e^ Race and ethnicity were self-reported. There were 2 variables: one for Asian, Black, White, and other (race [no further breakdown is available]), and another for Hispanic status. We created a single 5-level variable from the 2 variables.

^f^ Educational comparisons only included persons aged 22 years or older.

To examine age-related heterogeneity, data from respondents (eTable 7 in Supplement 1) were separated by age group. For persons aged 5 to 21 years (Table 2), the 5 most common disability diagnoses were attention-deficit disorder (ADD) or attention-deficit/hyperactivity disorder (ADHD) (40.1%; 95% CI, 37.1%-43.3%), anxiety or obsessive-compulsive disorder (OCD; 22.5%; 95% CI, 19.9%-25.3%), autism spectrum disorder or Asperger syndrome (autism; 17.2%; 95% CI, 14.9%-19.8%), depression (11.6%; 95% CI, 9.7%-13.8%), and learning disability (6.5%; 95% CI, 5.0%-8.4%). Within the cognitive disability category, the most common diagnoses for persons aged 5 to 21 years were ADD or ADHD (46.6%; 95% CI, 43.1%-50.2%), anxiety or OCD (25.4%; 95% CI, 22.4%-28.7%), autism (19.3%; 95% CI, 16.6%-22.3%), depression (13.4%; 95% CI, 11.2%-16.0%), and learning disability (7.5%; 95% CI, 5.8%-9.8%). Within the noncognitive disability category, the most common diagnoses for persons aged 5 to 21 years were blindness or vision problems (12.5%; 95% CI, 8.3%-18.3%), ADD or ADHD (11.2%; 95% CI, 7.7%-16.0%), deafness or hearing difficulty (10.9%; 95% CI, 7.2%-16.1%), anxiety or OCD (9.5%; 95% CI, 6.0%-14.9%), and autism (8.1%; 95% CI, 4.8%-13.1%).

**Table 2.** Most Common Disability Diagnoses Among Individuals Aged 5 to 21 Years

Diagnosis	Prevalence, % (95% CI)^a^
Overall	
Neurodevelopmental or neurobehavioral disorders: ADD or ADHD	40.1 (37.1-43.3)
Mental or emotional disorders: anxiety or obsessive-compulsive disorder	22.5 (19.9-25.3)
Neurodevelopmental or neurobehavioral disorders: autism spectrum disorder or Asperger syndrome	17.2 (14.9-19.8)
Mental or emotional disorders: depression	11.6 (9.7-13.8)
Neurodevelopmental or neurobehavioral disorders: learning disability	6.5 (5.0-8.4)
Cognitive disability	
Neurodevelopmental or neurobehavioral disorders: ADD or ADHD	46.6 (43.1-50.2)
Mental or emotional disorders: anxiety or obsessive-compulsive disorder	25.4 (22.4-28.7)
Neurodevelopmental or neurobehavioral disorders: autism spectrum disorder or Asperger syndrome	19.3 (16.6-22.3)
Mental or emotional disorders: depression	13.4 (11.2-16.0)
Neurodevelopmental or neurobehavioral disorders: learning disability	7.5 (5.8-9.8)
No cognitive disability	
Sensory or speech disorders: blindness or vision problems	12.5 (8.3-18.3)
Neurodevelopmental or neurobehavioral disorders: ADD or ADHD	11.2 (7.7-16.0)
Sensory or speech disorders: deafness or hearing difficulty	10.9 (7.2-16.1)
Mental or emotional disorders: anxiety or obsessive-compulsive disorder	9.5 (6.0-14.9)
Neurodevelopmental or neurobehavioral disorders: autism spectrum disorder or Asperger syndrome	8.1 (4.8-13.1)

Abbreviations: ADD, attention-deficit disorder; ADHD, attention-deficit/hyperactivity disorder.

^a^ Prevalence statistics show the percentage of the population of people with disabilities in question with a listed diagnosis; 95% CIs were calculated using logit transformation.

For persons aged 22 to 64 years (Table 3), the 5 most common disability diagnoses were anxiety or OCD (15.6%; 95% CI, 14.5%-16.9%), depression (15.3%; 95% CI, 14.1%-16.5%), unspecified musculoskeletal issues (13.5%; 95% CI, 12.5%-14.6%), back or spinal problems (11.6%; 95% CI, 10.6%-12.6%), and unspecified neurologic disorders (10.8%; 95% CI, 9.8%-11.8%). Within the cognitive disability category, the most common diagnoses for persons aged 22 to 64 years were depression (24.2%; 95% CI, 22.3%-26.2%), anxiety or OCD (23.7%; 95% CI, 21.9%-25.7%), ADD or ADHD (16.7%; 95% CI, 15.1%-18.5%), unspecified neurologic disorders (12.1%; 95% CI, 10.8%-13.6%), and back or spinal problems (9.5%; 95% CI, 8.3%-10.8%). Within the noncognitive disability category, the most common diagnoses for persons aged 22 to 64 years were unspecified musculoskeletal issues (19.3%; 95% CI, 17.5%-21.1%), back or spinal problems (13.9%; 95% CI, 12.5%-15.6%), arthritis (10.5%; 95% CI, 9.2%-11.9%), deafness or hearing difficulty (9.7%; 95% CI, 8.3%-11.2%), and unspecified neurologic disorders (9.3%; 95% CI, 8.1%-10.6%).

**Table 3.** Most Common Disability Diagnoses Among Individuals Aged 22 to 64 Years

Disability group and diagnosis	Prevalence, % (95% CI)^a^
Overall	
Mental or emotional disorders: anxiety or obsessive-compulsive disorder	15.6 (14.5-16.9)
Mental or emotional disorders: depression	15.3 (14.1-16.5)
Musculoskeletal issues: any other or unspecified	13.5 (12.5-14.6)
Musculoskeletal issues: back or spinal problems	11.6 (10.6-12.6)
Neurologic disorders: any other or unspecified	10.8 (9.8-11.8)
Cognitive disability	
Mental or emotional disorders: depression	24.2 (22.3-26.2)
Mental or emotional disorders: anxiety or obsessive-compulsive disorder	23.7 (21.9-25.7)
Neurodevelopmental or neurobehavioral disorders: ADD or ADHD	16.7 (15.1-18.5)
Neurologic disorders: any other or unspecified	12.1 (10.8-13.6)
Musculoskeletal issues: back or spinal problems	9.5 (8.3-10.8)
No cognitive disability	
Musculoskeletal issues: any other or unspecified	19.3 (17.5-21.1)
Musculoskeletal issues: back or spinal problems	13.9 (12.5-15.6)
Musculoskeletal issues: arthritis (other or unspecified)	10.5 (9.2-11.9)
Sensory or speech disorders: deafness or hearing difficulty	9.7 (8.3-11.2)
Neurologic disorders: any other or unspecified	9.3 (8.1-10.6)

Abbreviations: ADD, attention-deficit disorder; ADHD, attention-deficit/hyperactivity disorder.

^a^ Prevalence statistics show the percentage of the population of people with disabilities in question with a listed diagnosis; 95% CIs were calculated using logit transformation.

For persons aged 65 years or older (Table 4), the 5 most common disability diagnoses were unspecified musculoskeletal issues (18.8%; 95% CI, 17.7%-20.0%), arthritis (17.8%; 95% CI, 16.7%-18.9%), back or spinal problems (12.5%; 95% CI, 11.6%-13.5%), unspecified cardiovascular disorders (11.1%; 95% CI, 10.3%-12.0%), and deafness or hearing difficulty (9.7%; 95% CI, 8.9%-10.6%). Within the cognitive disability category, the most common diagnoses for persons aged 65 years or older were arthritis (16.9%; 95% CI, 15.1%-18.8%), unspecified musculoskeletal issues (16.6%; 95% CI, 14.8%-18.7%), unspecified neurologic disorders (14.8%; 95% CI, 13.1%-16.8%), unspecified cardiovascular disorders (12.3%; 95% CI, 10.8%-14.0%), and back or spinal problems (11.0%; 95% CI, 9.5%-12.8%). Within the noncognitive disability category, the most common diagnoses for persons aged 65 years or older were unspecified musculoskeletal issues (19.7%; 95% CI, 18.4%-21.2%), arthritis (18.2%; 95% CI, 16.9%-19.5%), back or spinal problems (13.2%; 95% CI, 12.0%-14.4%), deafness or hearing difficulty (11.6%; 95% CI, 10.6%-12.7%), and unspecified cardiovascular disorders (10.6%; 95% CI, 9.6%-11.7%).

**Table 4.** Most Common Disability Diagnoses Among Individuals Aged 65 Years or Older

Disability group and diagnosis	Prevalence, % (95% CI)^a^
Overall	
Musculoskeletal issues: any other or unspecified	18.8 (17.7-20.0)
Musculoskeletal issues: arthritis (other or unspecified)	17.8 (16.7-18.9)
Musculoskeletal issues: back or spine problems	12.5 (11.6-13.5)
Cardiovascular system disorders: any other or unspecified	11.1 (10.3-12.0)
Sensory or speech disorders: deafness or hearing difficulty	9.7 (8.9-10.6)
Cognitive disability	
Musculoskeletal issues: arthritis (other or unspecified)	16.9 (15.1-18.8)
Musculoskeletal issues: any other or unspecified	16.6 (14.8-18.7)
Neurologic disorders: any other or unspecified	14.8 (13.1-16.8)
Cardiovascular system disorders: any other or unspecified	12.3 (10.8-14.0)
Musculoskeletal issues: back or spinal problems	11.0 (9.5-12.8)
No cognitive disability	
Musculoskeletal issues: any other or unspecified	19.7 (18.4-21.2)
Musculoskeletal issues: arthritis (other or unspecified)	18.2 (16.9-19.5)
Musculoskeletal issues: back or spinal problems	13.2 (12.0-14.4)
Sensory or speech disorders: deafness or hearing difficulty	11.6 (10.6-12.7)
Cardiovascular system disorders: any other or unspecified	10.6 (9.6-11.7)

^a^ Prevalence statistics show the percentage of the population of people with disabilities in question with a listed diagnosis; 95% CIs were calculated using logit-transformation.

Table 5 reports prevalence estimates overall and disaggregated by each of the ACS-6 functional impairment questions for all diagnoses. Of people who indicated a cognitive disability via the ACS-6, only 2.8% (95% CI, 2.3%-3.4%) had an intellectual disability and 7.3% (95% CI, 6.4%-8.2%) were autistic. Only 0.5% (95% CI, 0.3%-0.8%) of respondents reporting a cognitive disability and fewer than 1.0% of respondents in any functional impairment category reported COVID-19 as 1 of their 3 most relevant diagnoses. eFigure 1 in Supplement 1 shows that the increase in cognitive disability rates during COVID-19 was equally visible in the SIPP, CPS, and ACS, supporting the generalizability of this finding. eFigure 2 in Supplement 1 shows percentages of respondents identified by the ACS-6 who reported no diagnosis (11.1% for persons aged 5 to 21 years, 10.3% for persons aged 22 to 64 years, and 11.7% for persons aged 65 years or older). eTables 9 to 12 and eFigure 3 in Supplement 1 show results from our primary analyses using only 2024 data. eTables 13 to 26 in Supplement 1 replicate data reported in Table 5 across demographic subgroups.

**Table 5.** Prevalence of Diagnoses Overall and by ACS-6 Question

Diagnosis	Prevalence, % (95% CI)^a^						
	Overall	Cognitive	Self-care	Mobility	Independent living	Hearing	Vision
Musculoskeletal issues: any other or unspecified	13.9 (13.2-14.6)	8.6 (7.8-9.4)	18.0 (16.1-20.0)	23.5 (22.3-24.8)	15.0 (13.7-16.4)	13.1 (11.9-14.4)	12.4 (11.0-13.9)
Mental or emotional disorders: anxiety or obsessive-compulsive disorder	12.0 (11.3-12.8)	20.1 (18.8-21.4)	9.0 (7.6-10.5)	6.9 (6.2-7.7)	13.7 (12.3-15.1)	6.2 (5.3-7.3)	8.6 (7.3-10.0)
Mental or emotional disorders: depression	10.8 (10.1-11.5)	18.3 (17.1-19.5)	9.3 (8.0-10.9)	8.1 (7.3-8.9)	11.8 (10.6-13.2)	6.4 (5.6-7.5)	8.6 (7.3-10.0)
Musculoskeletal issues: arthritis (other or unspecified)	10.5 (10.0-11.1)	6.9 (6.2-7.6)	12.8 (11.4-14.5)	17.8 (16.7-18.8)	11.9 (10.8-13.1)	11.1 (10.1-12.2)	9.8 (8.6-11.1)
Musculoskeletal issues: back or spinal problems	10.5 (9.9-11.1)	7.6 (7.1-8.7)	15.6 (13.8-17.6)	16.9 (15.9-18.0)	12.3 (11.1-13.6)	10.7 (9.6-11.9)	8.6 (7.5-9.9)
Neurodevelopmental or neurobehavioral disorders: ADD or ADHD	10.4 (9.8-11.1)	19.9 (18.7-21.3)	4.6 (3.7-5.8)	2.4 (2.0-2.9)	5.1 (4.3-6.1)	2.9 (2.3-3.6)	3.9 (3.1-4.9)
Neurologic disorders: any other or unspecified	9.2 (8.6-9.8)	10.4 (9.5-11.4)	16.0 (14.2-18.1)	13.2 (12.2-14.3)	13.6 (12.3-15.0)	8.1 (7.2-9.2)	8.9 (7.7-10.4)
Cardiovascular system disorders: any other or unspecified	7.0 (6.5-7.5)	5.5 (4.9-6.2)	10.0 (8.6-11.5)	10.6 (9.8-11.5)	9.9 (8.8-11.0)	8.7 (7.8-9.7)	7.7 (6.7-8.8)
Sensory or speech disorders: deafness or hearing difficulty	6.7 (6.2-7.2)	2.4 (2.0-2.9)	3.00 (2.2-4.0)	2.6 (2.2-3.1)	2.9 (2.3-3.5)	22.9 (21.4-24.6)	3.4 (2.7-4.2)
Other: any other conditions, including those not sufficiently specific to classify	5.5 (5.1-6.0)	6.3 (5.6-7.2)	7.2 (6.0-8.6)	7.0 (6.3-7.7)	6.9 (6.0-7.9)	4.1 (3.4-4.8)	4.6 (3.8-5.6)
Endocrine disorders: diabetes	5.5 (5.1-5.9)	4.6 (4.1-5.3)	8.2 (6.9-9.6)	8.3 (7.5-9.1)	7.9 (6.9-8.9)	5.0 (4.3-5.8)	7.5 (6.4-8.7)
Sensory or speech disorders: blindness or vision problems	5.1 (4.7-5.6)	2.9 (2.4-3.5)	4.6 (3.7-5.8)	3.8 (3.3-4.4)	5.6 (4.8-6.5)	3.9 (3.3-4.6)	20.8 (18.9-22.7)
Cardiovascular system disorders: high blood pressure	4.4 (4.0-4.8)	3.4 (3.0-3.9)	5.4 (4.5-6.6)	5.9 (5.3-6.6)	5.1 (4.4-5.9)	4.3 (3.7-5.1)	5.6 (4.7-6.6)
Neurodevelopmental or neurobehavioral disorders: autism spectrum disorder or Asperger syndrome	3.9 (3.5-4.4)	7.3 (6.4-8.2)	5.5 (4.4-6.9)	1.2 (0.9-1.7)	4.9 (4.0-6.0)	1.3 (0.9-1.9)	1.9 (1.3-2.7)
Mental or emotional disorders: any other or unspecified	3.5 (3.1-3.9)	6.3 (5.5-7.1)	3.5 (2.6-4.7)	2.4 (1.9-2.9)	4.8 (4.0-5.8)	2.0 (1.5-2.7)	3.0 (2.3-4.1)
Mental or emotional disorders: bipolar disorder	3.2 (2.8-3.6)	5.9 (5.1-6.7)	3.9 (2.9-5.4)	2.5 (2.0-3.0)	4.3 (3.5-5.3)	2.2 (1.7-2.8)	2.6 (1.8-3.7)
Mental or emotional disorders: trauma or stressor-related disorder	3.0 (2.7-3.5)	4.9 (4.3-5.7)	3.7 (2.7-5.1)	2.6 (2.2-3.2)	3.1 (2.5-3.9)	2.7 (2.1-3.4)	2.1 (1.5-3.0)
Other: pain (unspecified)	2.8 (2.5-3.2)	3.3 (2.8-3.8)	4.9 (3.8-6.2)	4.2 (3.7-4.9)	4.2 (3.5-5.1)	2.1 (1.6-2.7)	3.0 (2.3-3.9)
Respiratory disorders: COPD	2.3 (2.1-2.7)	1.8 (1.5-2.2)	3.8 (3.00-4.7)	3.8 (3.4-4.4)	3.9 (3.3-4.6)	2.8 (2.3-3.4)	2.5 (1.8-3.4)
Neurologic disorders: stroke or brain aneurysm	2.2 (1.9-2.5)	2.7 (2.3-3.2)	4.6 (3.7-5.7)	3.4 (2.9-3.9)	4.0 (3.3-4.7)	2.5 (2.0-3.1)	2.4 (1.9-3.2)
Cancer, tumor, cyst, or growth	2.0 (1.8-2.3)	1.8 (1.4-2.2)	3.2 (2.4-4.2)	2.7 (2.3-3.2)	3.3 (2.7-4.1)	2.3 (1.9-2.9)	2.2 (1.6-2.9)
Neurodevelopmental or neurobehavioral disorders: learning disability	2.0 (1.7-2.3)	3.3 (2.7-4.0)	1.0 (0.6-1.8)	1.0 (0.7-1.3)	1.4 (1.0-1.9)	0.8 (0.6-1.2)	1.2 (0.7-1.9)
Immune system disorders	1.7 (1.5-2.0)	2.0 (1.6-2.5)	2.6 (1.8-3.6)	2.2 (1.8-2.7)	2.4 (1.8-3.1)	1.1 (0.8-1.6)	2.0 (1.4-2.9)
Respiratory disorders: asthma	1.7 (1.4-2.0)	1.6 (1.2-2.1)	2.2 (1.5-3.2)	1.8 (1.5-2.2)	1.6 (1.2-2.2)	1.2 (0.8-1.7)	1.8 (1.1-2.8)
Digestive system disorders (including liver conditions, stomach problems)	1.7 (1.4-2.0)	2.0 (1.6-2.4)	1.9 (1.4-2.7)	2.0 (1.7-2.5)	2.0 (1.5-2.5)	1.4 (1.1-1.9)	1.9 (1.2-2.8)
Sensory or speech disorders: any other or unspecified (including speech disorders, vestibular problems)	1.6 (1.4-1.9)	1.4 (1.1-1.9)	2.1 (1.6-2.9)	2.2 (1.8-2.6)	2.4 (1.9-3.0)	2.1 (1.6-2.6)	1.5 (1.1-2.1)
Musculoskeletal issues: rheumatoid arthritis	1.6 (1.4-1.9)	1.2 (0.9-1.6)	3.0 (2.3-3.9)	2.7 (2.3-3.2)	2.2 (1.7-2.7)	1.2 (0.8-1.6)	1.8 (1.3-2.5)
Neurodevelopmental or neurobehavioral disorders: any other and unspecified	1.6 (1.3-1.9)	2.7 (2.2-3.3)	3.8 (2.9-5.2)	1.4 (1.0-1.8)	2.5 (1.8-3.3)	0.6 (0.4-1.1)	1.6 (1.1-2.4)
Neurodevelopmental or neurobehavioral disorders: intellectual disability (due to congenital disorder [eg, Down syndrome] or other cause)	1.5 (1.3-1.9)	2.8 (2.3-3.4)	4.4 (3.2-5.9)	1.3 (0.9-1.7)	3.6 (2.8-4.5)	0.6 (0.4-0.9)	1.0 (0.6-1.7)
Other: aging	1.5 (1.3-1.7)	1.1 (0.9-1.4)	2.3 (1.7-3.1)	2.1 (1.8-2.5)	2.3 (1.9-2.8)	2.7 (2.2-3.3)	1.6 (1.2-2.2)
Respiratory disorders: any other or unspecified	1.5 (1.3-1.7)	1.2 (0.9-1.6)	2.5 (1.8-3.4)	2.3 (2.0-2.8)	2.2 (1.8-2.8)	1.4 (1.0-1.9)	1.2 (0.8-1.7)
Genitourinary disorders	1.4 (1.2-1.6)	1.2 (0.9-1.6)	2.6 (1.9-3.5)	1.9 (1.6-2.3)	2.2 (1.7-2.8)	1.3 (1.0-1.8)	2.1 (1.5-2.8)
Neurologic disorders: dementia or Alzheimer disease	1.4 (1.2-1.6)	2.7 (2.4-3.2)	4.6 (3.8-5.5)	2.1 (1.8-2.5)	4.1 (3.5-4.8)	2.0 (1.6-2.4)	1.7 (1.3-2.2)
Neurologic disorders: epilepsy or seizures	1.1 (0.9-1.4)	1.8 (1.4-2.3)	2.0 (1.4-2.9)	1.0 (0.7-1.3)	2.4 (1.9-3.2)	0.7 (0.4-1.1)	1.0 (0.7-1.6)
Endocrine disorders: any other or unspecified	0.8 (0.7-1.1)	0.6 (0.4-0.9)	1.1 (0.7-1.8)	1.1 (0.9-1.5)	0.8 (0.5-1.3)	0.8 (0.5-1.2)	0.6 (0.4-1.1)
Other: COVID-19 or coronavirus	0.4 (0.2-0.5)	0.5 (0.3-0.8)	0.8 (0.5-1.6)	0.5 (0.3-0.9)	0.7 (0.4-1.2)	0.2 (0.1-0.4)	0.2 (0.1-0.7)

Abbreviations: ACS-6, American Community Survey-6; ADD, attention-deficit disorder; ADHD, attention-deficit/hyperactivity disorder; COPD, chronic obstructive pulmonary disease.

^a^ Prevalence statistics show the percentage of the population of people with disabilities identified by each of the ACS-6 questions (described by the column titles) with the listed diagnosis; 95% CIs were calculated using logit transformation.

Diagnoses are relatively constant across demographic subgroups. Some exceptions exist; for instance, the prevalence of intellectual disability was lower among college-educated persons, and the prevalence of autism was higher among male respondents. However, much like the differences across age groups, these largely corresponded to expected differences documented in prior literature,^30,31^ suggesting that there are no observable differences in whom the ACS-6 identifies across demographic subgroups other than age.

## Discussion

Results of this cross-sectional study show a highly heterogeneous population of people with disabilities in which diagnoses were most distinct across age groups but identified a similar population across other demographic categories. That these results were derived from a federal population survey provides them with added credibility. One challenge to disability measurement research taking place outside this context is the lack of proxy respondents in telephone-based and online-based sample collection, effectively removing the substantial portion of the disabled population identified only via proxy response. Use of the SIPP, which incorporates proxy respondents, to examine the ACS-6 avoids this problem and promotes comparability with the ACS and CPS.

The low rates of COVID-19 reporting as a diagnosis causing functional impairment substantially complicates the prevailing narrative that the increase in cognitive disability in surveys using the ACS-6 is directly attributable to long COVID, although it remains possible that persons with long COVID may be reporting more salient conditions or that the US Census Bureau might have grouped respondents reporting long COVID under other diagnoses. An alternative theory for the persistence of the COVID-era increase in cognitive disability might be found in the high proportions of people with cognitive disabilities reporting a diagnosis of depression (20.1%) and anxiety/OCD (18.3%), as listed in Table 5. Other work has documented a substantial increase in depression and anxiety during the COVID-19 public health emergency.^32,33^ These findings suggest that these mental health conditions are potentially associated with the increase in cognitive disability status. A distinct but compatible theory might be found in the increase in screen time documented among both children and adults during COVID-19, potentially associated with rates of both mental health conditions and other forms of impairment.^34^ Future research should more closely examine the causes of the increase in cognitive disability rates, a substantial public health concern and among the most important compositional shifts in the disabled population since the ACS-6 introduction in 2008.

The ACS-6 was designed with the goal of capturing a population of people with disabilities that more closely corresponds to the broad civil rights protections of the Americans with Disabilities Act of 1990, which encompass many who do not think of themselves as disabled.^29^ It is thus appropriate that the ACS-6 identifies both diagnoses clearly socially constructed as disabilities, such as autism and blindness, as well as diagnoses that qualify for Americans with Disabilities Act protections but are often constructed as chronic health conditions, such as depression and chronic obstructive pulmonary disease. Although this breadth is important, it does present methodologic challenges for operationalizing the measurement of disability health disparities. For instance, recent work by Juhasz and Byers^4^ assessed the rate of chronic condition prevalence among people with disabilities identified using the ACS-6. The insights provided by the SIPP into diagnoses captured by the ACS-6 complicate such findings, since in many cases the chronic conditions being studied are in whole or in part the reason a person reports an ACS-6 functional impairment, meaning high rates of chronic condition prevalence follow mechanically.

This illustrates a conceptual challenge that must be addressed to meaningfully implement the recent recognition of people with disabilities as a population with health disparities; disability is both an input into and an output of disparities. Many of the methodologic approaches for operationalizing racial disparities in health care access and use, which rely on setting constant health status between majority and minority groups, will require adaptation or replacement for a context in which health status is part of the definition of the minority population.^35,36^

Our findings demonstrate that both the ACS-6 as a whole and each individual ACS-6 question include a wide diversity of diagnoses. Caution is needed in matching ACS-6 functional impairment questions to specific research questions. Given that our results show low rates of intellectual disability and autism diagnoses among persons with a cognitive disability, use of the cognitive disability variable alone to evaluate policy changes specific to the I/DD service system may be ill advised. Additional refinement may be necessary to align the population of people with disabilities being studied with the relevant policy context. This may involve making use of combinations of disability questions on the ACS-6, potentially with other demographic variables. Clemens et al^9^ offer such an approach by making use of ACS data to impute a severe disability category by building a model based on veterans’ disability ratings and applying it to the civilian population of people with disabilities. The disability diagnoses embedded within the SIPP offer a pathway for further improvement, such as making use of SIPP data to impute diagnoses into higher-frequency datasets, similar to prior work by Kruse et al,^37^ who used the ACS-6 to impute cancer survivorship from the Behavioral Risk Factor Surveillance System into the CPS.

Such efforts would likely benefit from a more granular approach to disability data collection. A 2023 proposal from the Census Bureau to shift the ACS to a modified version of the Washington Group Short Set (WG-SS), which asks respondents to report their degree of difficulty with a given activity from 4 options, offers a viable alternative.^38^ Because the modified WG-SS would yield a lower reported disability prevalence because of its recommended cutoff of requiring respondents to report a lot of difficulty with or complete inability to perform a task before they are recognized as having a disability, it prompted criticism that the new question set “artificially reduced the estimate of the US disabled population,” leading the Census Bureau to freeze the plan.^39,40^

However, the more nuanced approach to collecting disability data with the WG-SS would have given researchers additional capacity to examine the diversity of disability experiences (and a larger population of people with disabilities could still be identified via the WG-SS by using a more lenient cutoff point).^41^ Such flexible measurement approaches may better allow researchers to examine the heterogeneity highlighted by this study’s results and match a research question to an appropriate population of people with disabilities.^42^ Rather than seeking to identify as many members as possible of a single unitary population of people with disabilities, survey designers should acknowledge the need to examine many different populations of people with disabilities and adopt measures like the WG-SS that better enable tailoring the group of people with disabilities identified to the research question at issue.

### Limitations

Although the SIPP is a nationally representative probability sample, similar to most surveys using the ACS-6 (except the ACS itself) it does not include institutionalized persons within its sampling frame. Because more specific diagnoses were not available from the Census Bureau, it was not possible to examine the heterogeneity reflected within broad categories. Because the SIPP allows respondents to list only 3 diagnoses, it may also underestimate the prevalence of conditions that are not salient enough relative to other comorbidities to be listed in the top 3 by the respondent. As respondents were asked to report diagnoses for all functional impairments they reported, it was not possible to ascertain which diagnoses corresponded to which ACS-6 questions or other SIPP disability screening questions.

## Conclusions

The findings of this study show that the ACS-6 identifies a broad and diverse population of people with disabilities. Future disability research should account for the heterogeneity of the population of people with disabilities in both the use of existing data resources and the revision of existing approaches to disability data collection.

## Notes

Supplement 1. **eAppendix.** Details on Generation of Logit-Transformed 95% Confidence Intervals **eTable 1.** Disability Identification Questions Making Up the ACS-6 **eTable 2.** Comparing Functional Impairment Combinations Across the 2023/2024 SIPP, 2023 ACS and 2023 ASEC - Overall and by Cognitive Disability Status **eTable 3.** Ranking of Functional Impairment Combinations Across the SIPP, ACS and ASEC - Overall **eTable 4.** Ranking of Functional Impairment Combinations Across the SIPP, ACS and ASEC - Cognitive Disability Only **eTable 5.** Ranking of Functional Impairment Combinations Across the SIPP, ACS and ASEC - Non-Cognitive Disability Only **eTable 6.** Correlation Coefficients Limited to Top 15 Most Common Diagnoses **eTable 7.** Most Common Disability Diagnoses, Cognitive and Non-Cognitive Disability Populations **eTable 8.** Diagnosis Groups, Overall - Cognitive and Non-Cognitive Disability **eTable 9.** Most Common Diagnosis Groups, Cognitive and Non-Cognitive Disability Populations (2024 Data Only) **eTable 10.** Correlation Coefficients, By Cognitive Disability Status (2024 Data Only) **eTable 11.** Most Common Diagnosis Groups By Age Group, Overall and by Cognitive Disability Status (2024 Data Only) **eTable 12.** Prevalence of Diagnosis Groups, Overall and by ACS-6 Question (2024 Data Only) **eTable 13.** Prevalence of Diagnoses, Overall and by ACS-6 Question - Age 5-21 Only **eTable 14.** Prevalence of Diagnoses, Overall and by ACS-6 Question - Age 22-64 Only **eTable 15.** Prevalence of Diagnoses, Overall and by ACS-6 Question - Age 65+ Only **eTable 16.** Prevalence of Diagnoses, Overall and by ACS-6 Question - White Only **eTable 17.** Prevalence of Diagnoses, Overall and by ACS-6 Question - Black Only **eTable 18.** Prevalence of Diagnoses, Overall and by ACS-6 Question - Hispanic Only **eTable 19.** Prevalence of Diagnoses, Overall and by ACS-6 Question - Asian Only **eTable 20.** Prevalence of Diagnoses, Overall and by ACS-6 Question - Other Only **eTable 21.** Prevalence of Diagnoses, Overall and by ACS-6 Question - Male Only **eTable 22.** Prevalence of Diagnoses, Overall and by ACS-6 Question - Female Only **eTable 23.** Prevalence of Diagnoses, Overall and by ACS-6 Question - No HS Diploma Only **eTable 24.** Prevalence of Diagnoses, Overall and by ACS-6 Question - HS Diploma **eTable 25.** Prevalence of Diagnoses, Overall and by ACS-6 Question - Some College **eTable 26.** Prevalence of Diagnoses, Overall and by ACS-6 Question - BA+ **eFigure 1.** Frequency of Reporting No Diagnosis, By Age Group **eFigure 2.** Frequency of Reporting No Diagnosis, By Age Group (2024 Data Only) **eFigure 3.** Change Over Time in Cognitive Disability Status Relative to 2019, 2018-2023

Supplement 2.
Data Sharing Statement
