# Supplementary material for: Disability Diagnoses Identified by the American Community Survey 6-Question Sequence
Source: JAMA Health Forum. 2026 Jan 23;7(1):e256302. doi: 10.1001/jamahealthforum.2025.6302 (PMC12831152; doi:10.1001/jamahealthforum.2025.6302)
Supplement: Supplement 1. — eAppendix. Details on Generation of Logit-Transformed 95% Confidence Intervals eTable 1. Disability Identification Questions Making Up the ACS-6 eTable 2. Comparing Functional Impairment Combinations Across the 2023/2024 SIPP, 2023 ACS and 2023 ASEC - Overall and by Cognitive Disability Status eTable 3. Ranking of Functional Impairment Combinations Across the SIPP, ACS and ASEC - Overall eTable 4. Ranking of Functional Impairment Combinations Across the SIPP, ACS and ASEC - Cognitive Disability Only eTable 5. Ranking of Functional Impairment Combinations Across the SIPP, ACS and ASEC - Non-Cognitive Disability Only eTable 6. Correlation Coefficients Limited to Top 15 Most Common Diagnoses eTable 7. Most Common Disability Diagnoses, Cognitive and Non-Cognitive Disability Populations eTable 8. Diagnosis Groups, Overall - Cognitive and Non-Cognitive Disability eTable 9. Most Common Diagnosis Groups, Cognitive and Non-Cognitive Disability Populations (2024 Data Only) eTable 10. Correlation Coefficients, By Cognitive Disability Status (2024 Data Only) eTable 11. Most Common Diagnosis Groups By Age Group, Overall and by Cognitive Disability Status (2024 Data Only) eTable 12. Prevalence of Diagnosis Groups, Overall and by ACS-6 Question (2024 Data Only) eTable 13. Prevalence of Diagnoses, Overall and by ACS-6 Question - Age 5-21 Only eTable 14. Prevalence of Diagnoses, Overall and by ACS-6 Question - Age 22-64 Only eTable 15. Prevalence of Diagnoses, Overall and by ACS-6 Question - Age 65+ Only eTable 16. Prevalence of Diagnoses, Overall and by ACS-6 Question - White Only eTable 17. Prevalence of Diagnoses, Overall and by ACS-6 Question - Black Only eTable 18. Prevalence of Diagnoses, Overall and by ACS-6 Question - Hispanic Only eTable 19. Prevalence of Diagnoses, Overall and by ACS-6 Question - Asian Only eTable 20. Prevalence of Diagnoses, Overall and by ACS-6 Question - Other Only eTable 21. Prevalence of Diagnoses, Overall and by ACS-6 Question - Male Only eTable 22. [file jamahealthforum-e256302-s001.pdf]

Supplemental Online Content

Ne'eman A. Disability diagnoses identified by the American Community Survey 6-Question Sequence *JAMA Health Forum*. 2026;7(1):e256302. doi:10.1001/jamahealthforum.2025.6302

eAppendix. Details on Generation of Logit-Transformed 95% Confidence Intervals

eTable 1. Disability Identification Questions Making Up the ACS-6

eTable 2. Comparing Functional Impairment Combinations Across the 2023/2024 SIPP, 2023 ACS and 2023 ASEC - Overall and by Cognitive Disability Status

eTable 3. Ranking of Functional Impairment Combinations Across the SIPP, ACS and ASEC - Overall

eTable 4. Ranking of Functional Impairment Combinations Across the SIPP, ACS and ASEC - Cognitive Disability Only

eTable 5. Ranking of Functional Impairment Combinations Across the SIPP, ACS and ASEC - Non-Cognitive Disability Only

eTable 6. Correlation Coefficients Limited to Top 15 Most Common Diagnoses

eTable 7. Most Common Disability Diagnoses, Cognitive and Non-Cognitive Disability Populations

eTable 8. Diagnosis Groups, Overall - Cognitive and Non-Cognitive Disability

eTable 9. Most Common Diagnosis Groups, Cognitive and Non-Cognitive Disability Populations (2024 Data Only)

eTable 10. Correlation Coefficients, By Cognitive Disability Status (2024 Data Only)

eTable 11. Most Common Diagnosis Groups By Age Group, Overall and by Cognitive Disability Status (2024 Data Only)

eTable 12. Prevalence of Diagnosis Groups, Overall and by ACS-6 Question (2024 Data Only)

eTable 13. Prevalence of Diagnoses, Overall and by ACS-6 Question - Age 5-21 Only

eTable 14. Prevalence of Diagnoses, Overall and by ACS-6 Question - Age 22-64 Only

eTable 15. Prevalence of Diagnoses, Overall and by ACS-6 Question - Age 65+ Only

eTable 16. Prevalence of Diagnoses, Overall and by ACS-6 Question - White Only

eTable 17. Prevalence of Diagnoses, Overall and by ACS-6 Question - Black Only

eTable 18. Prevalence of Diagnoses, Overall and by ACS-6 Question - Hispanic Only

eTable 19. Prevalence of Diagnoses, Overall and by ACS-6 Question - Asian Only

eTable 20. Prevalence of Diagnoses, Overall and by ACS-6 Question - Other Only

eTable 21. Prevalence of Diagnoses, Overall and by ACS-6 Question - Male Only

eTable 22. Prevalence of Diagnoses, Overall and by ACS-6 Question - Female Only

eTable 23. Prevalence of Diagnoses, Overall and by ACS-6 Question - No HS Diploma Only

eTable 24. Prevalence of Diagnoses, Overall and by ACS-6 Question - HS Diploma

© 2026 Ne'eman A. *JAMA Health Forum*.

eTable 25. Prevalence of Diagnoses, Overall and by ACS-6 Question - Some College
eTable 26. Prevalence of Diagnoses, Overall and by ACS-6 Question - BA+
eFigure 1. Frequency of Reporting No Diagnosis, By Age Group
eFigure 2. Frequency of Reporting No Diagnosis, By Age Group (2024 Data Only)
eFigure 3. Change Over Time in Cognitive Disability Status Relative to 2019, 2018-2023

© 2026 Ne'eman A. *JAMA Health Forum*.

APPENDIX:

Details on Generation of Logit-Transformed 95% Confidence Intervals:

The logit-transformed CIs are calculated first in the logit (log-odds) space and then transformed into the probability space. We specifically use the svyciprop command in R using the logit method. This command first estimates an intercept-only logistic regression model with a binary outcome, which estimates the log-odds and its standard error. A Wald-type 95% confidence interval is calculated in the log-odds space. Then, the bounds of the confidence interval are transformed into the probability space using the inverse logit function. This logit-transformation ensures that the confidence intervals are bound by (0,1) and improves accuracy when the estimated proportion is close to 0 or 1.

Appendix Exhibits:

Appendix Table S1: Disability Identification Questions Making Up the ACS-6

Deaf	Are you deaf, or do you have serious difficulty hearing?
Blind	Are you blind, or do you have serious difficulty seeing, even when wearing glasses?
Cognitive	Because of a physical, mental, or emotional condition, do you have serious difficulty concentrating, remembering, or making decisions?
Mobility	Do you have serious difficulty walking or climbing stairs?
Self-Care	Do you have difficulty dressing or bathing?
Independent Living	Because of a physical, mental, or emotional condition, do you have difficulty doing errands alone such as visiting a doctor's office or shopping?

Notes: ACS-6 indicates the 6-item disability sequence used in federal surveys.

© 2026 Ne’eman A. *JAMA Health Forum*.

Appendix Table S2: Comparing Functional Impairment Combinations Across the 2023/2024 SIPP, 2023 ACS and 2023 ASEC - Overall and by Cognitive Disability Status

	**Overall**		**Cognitive**		**Non-Cognitive**	
	**Spearman’s**	**Pearson’s**	**Spearman’s**	**Pearson’s**	**Spearman’s**	**Pearson’s**
**SIPP vs. ASEC**	0.857***	0.845***	0.764**	0.959***	0.968***	0.975***
**SIPP vs. ACS**	0.882***	0.948***	0.761**	0.967***	0.979***	0.988***

© 2026 Ne’eman A. *JAMA Health Forum*.

Appendix Table S3: Ranking of Functional Impairment Combinations Across the SIPP, ACS and ASEC - Overall

**Overall**						
	**2023/2024 SIPP**		**2023 ASEC**		**2023 ACS**	
**15 Most Common ACS-6 Disability Diagnoses in 2023/2024 SIPP**	**Rank**	**Prevalence**	**Rank**	**Prevalence**	**Rank**	**Prevalence**
Cognitive	1	19.67%	3	11.28%	1	14.52%
Mobility	2	13.87%	1	19.45%	2	14.51%
Hearing	3	11.70%	2	14.63%	3	12.66%
Vision	4	6.61%	6	4.79%	4	6.77%
Cognitive + Indep. Living	5	4.00%	5	4.99%	5	5.80%
Cognitive + Mobility	6	3.33%	12	2.10%	12	1.91%
Cognitive + Self-Care + Mobility + Indep. Living	7	3.23%	8	3.58%	6	4.48%
Mobility + Indep. Living	8	3.13%	4	5.47%	8	4.30%
Self-Care + Mobility + Indep. Living	9	2.84%	7	4.25%	7	4.38%
Mobility + Hearing	10	2.52%	9	3.30%	10	2.36%
Cognitive + Mobility + Indep. Living	11	2.40%	11	2.30%	11	2.02%
Cognitive + Hearing	12	2.00%	14	1.19%	19	1.07%
Indep. Living	13	1.59%	10	3.18%	9	3.70%
Cognitive + Vision	14	1.43%	26	0.56%	21	0.92%
Hearing + Vision	15	1.37%	19	0.95%	16	1.20%

© 2026 Ne’eman A. *JAMA Health Forum*.

Appendix Table S4: Ranking of Functional Impairment Combinations Across the SIPP, ACS and ASEC - Cognitive Disability Only

Cognitive Disability						
	2023/2024 SIPP		2023 ASEC		2023 ACS	
15 Most Common ACS-6 Disability Diagnoses in 2023/2024 SIPP	Rank	Prevalence	Rank	Prevalence	Rank	Prevalence
Cognitive	1	40.77%	1	32.41%	1	35.15%
Cognitive + Indep. Living	2	8.28%	2	14.34%	2	14.04%
Cognitive + Mobility	3	6.90%	5	6.05%	5	4.62%
Cognitive + Self-Care + Mobility + Indep. Living	4	6.70%	3	10.29%	3	10.84%
Cognitive + Mobility + Indep. Living	5	4.97%	4	6.62%	4	4.89%
Cognitive + Hearing	6	4.14%	6	3.41%	10	2.58%
Cognitive + Vision	7	2.97%	13	1.61%	11	2.22%
Cognitive + Self-Care + Mobility + Indep. Living + Vision	8	2.80%	12	1.93%	9	2.58%
Cognitive + Self-Care + Mobility + Indep. Living + Hearing + Vision	9	2.36%	7	2.93%	6	3.16%
Cognitive + Mobility + Hearing	10	2.33%	11	2.07%	15	1.25%
Cognitive + Self-Care + Mobility + Indep. Living + Hearing	11	2.05%	8	2.87%	7	2.94%
Cognitive + Self-Care	12	1.69%	24	0.35%	12	1.76%
Cognitive + Mobility + Vision	13	1.60%	16	1.00%	20	0.83%

© 2026 Ne’eman A. *JAMA Health Forum*.

Cognitive + Mobility + Indep. Living + Hearing	14	1.58%	9	2.55%	13	1.42%
Cognitive + Self-Care + Indep. Living	15	1.53%	10	2.35%	8	2.62%

© 2026 Ne’eman A. *JAMA Health Forum*.

Appendix Table S5: Ranking of Functional Impairment Combinations Across the SIPP, ACS and ASEC - Non-Cognitive Disability Only

Non-Cognitive Disability						
	**2023/2024 SIPP**		**2023 ASEC**		**2023 ACS**	
**15 Most Common ACS-6 Disability Diagnoses in 2023/2024 SIPP**	**Rank**	**Prevalence**	**Rank**	**Prevalence**	**Rank**	**Prevalence**
Mobility	1	26.81%	1	29.83%	1	24.72%
Hearing	2	22.44%	2	22.44%	2	21.57%
Vision	3	12.78%	4	7.35%	3	11.54%
Mobility + Indep. Living	4	6.05%	3	8.39%	5	7.33%
Self-Care + Mobility + Indep. Living	5	5.49%	5	6.52%	4	7.46%
Mobility + Hearing	6	4.87%	6	5.06%	7	4.02%
Indep. Living	7	3.07%	7	4.88%	6	6.31%
Hearing + Vision	8	2.65%	11	1.46%	9	2.05%
Mobility + Vision	9	2.59%	9	1.70%	10	1.77%
Self-Care + Mobility	10	2.25%	8	2.08%	8	2.22%
Mobility + Indep. Living + Hearing	11	1.45%	10	1.66%	11	1.42%
Self-Care + Mobility + Indep. Living + Hearing	12	1.26%	12	0.99%	12	1.30%
Mobility + Indep. Living + Vision	13	1.24%	13	0.98%	13	1.04%
Mobility + Hearing + Vision	14	1.16%	18	0.71%	17	0.82%
Self-Care + Mobility + Indep. Living + Vision	15	0.92%	15	0.78%	14	0.98%

© 2026 Ne’eman A. *JAMA Health Forum*.

Appendix Table S6: Correlation Coefficients Limited to Top 15 Most Common Diagnoses

	Cognitive		Non-Cognitive	
	Spearman's	Pearson's	Spearman's	Pearson's
Age Group				
5-21 vs 22-64	0.664**	0.638*	0.082	-0.083
22-64 vs 65+	0.039	-0.112	0.889***	0.885***
5-21 vs. 65+	-0.611*	-0.554*	-0.204	-0.235
Sex				
Male vs Female	0.768**	0.794***	0.929***	0.842***
Race/Ethnicity				
White vs Black	0.704**	0.870***	0.861***	0.870***
White vs Hispanic	0.821***	0.962***	0.814***	0.861***
White vs Asian	0.668**	0.831***	0.870***	0.920***
White vs Other	0.854***	0.931***	0.914***	0.909***
Black vs Hispanic	0.889***	0.925***	0.864***	0.871***
Black vs Asian	0.564*	0.690**	0.831***	0.880***
Black vs Other	0.686**	0.826***	0.900***	0.900***
Asian vs Hispanic	0.546*	0.794***	0.931***	0.948***
Asian vs Other	0.443	0.794***	0.931***	0.904***
Hispanic vs Other	0.743**	0.896***	0.843***	0.810***
Education*				
No HS vs HS Diploma	0.764**	0.697**	0.861***	0.896***
No HS vs Some College	0.675**	0.623*	0.875***	0.896***
No HS vs BA+	0.614*	0.372	0.818***	0.772***

© 2026 Ne'eman A. *JAMA Health Forum*.

**HS Diploma vs Some College**	**0.957*** **	**0.969*** **	**0.964*** **	**0.986*** **
**HS Diploma vs BA+**	**0.857*** **	**0.844*** **	**0.954*** **	**0.949*** **
**Some College vs BA+**	**0.907*** **	**0.916*** **	**0.979*** **	**0.944*** **

© 2026 Ne’eman A. *JAMA Health Forum*.

Appendix Table S7: Most Common Disability Diagnoses, Cognitive and Non-Cognitive Disability Populations

Rank	Overall		With Cognitive		Without Cognitive	
	Condition	Prevalence	Condition	Prevalence	Condition	Prevalence
1	Musculoskeletal issues: Any other or unspecified	13.87% [13.19%-14.58%]	Mental or emotional disorders: Anxiety or obsessive-compulsive disorders	20.05% [18.78%-21.38%]	Musculoskeletal issues: Any other or unspecified	18.81% [17.76%-19.92%]
2	Mental or emotional disorders: Anxiety or obsessive-compulsive disorders	12.01% [11.30%-12.75%]	Neurodevelopmental/behavioral: ADD or ADHD	19.93% [18.66%-21.27%]	Musculoskeletal issues: Arthritis (other or unspecified)	13.92% [13.03%-14.86%]
3	Mental or emotional disorders: Depression	10.79% [10.12%-11.49%]	Mental or emotional disorders: Depression	18.26% [17.05%-19.54%]	Musculoskeletal issues: Back or spine problems	12.93% [12.04%-13.86%]
4	Musculoskeletal issues: Arthritis (other or unspecified)	10.53% [9.96%-11.13%]	Neurological disorders: Any other or unspecified	10.43% [9.53%-11.39%]	Sensory/speech disorders: Deafness or hearing difficulty	10.72% [9.88%-11.62%]

© 2026 Ne'eman A. *JAMA Health Forum*.

5	Musculoskeletal issues: Back or spine problems	10.48% [9.88%-11.10%]	Musculoskeletal issues: Any other or unspecified	8.57% [7.79%-9.43%]	Cardiovascular system disorders: Any other or unspecified	8.28% [7.57%-9.05%]

Prevalence statistics show the percentage of the disabled population in question (overall disabled population in the top panel, cognitive disability population in the middle panel, non-cognitive disability population in the last panel) with the listed diagnosis. 95% Confidence Intervals are in brackets calculated using logit-transformation.

© 2026 Ne’eman A. *JAMA Health Forum*.

Appendix Table S8: Diagnosis Groups, Overall - Cognitive and Non-Cognitive Disability

Condition	Overall	Cognitive Disability	No Cognitive Disability
**Musculoskeletal issues: Any other or unspecified**	13.87% [13.19%-14.58%]	8.57% [7.79%-9.43%]	18.81% [17.76%-19.92%]
**Mental or emotional disorders: Anxiety or obsessive-compulsive disorders**	12.01% [11.30%-12.75%]	20.05% [18.78%-21.38%]	4.50% [3.93%-5.15%]
**Mental or emotional disorders: Depression**	10.79% [10.12%-11.49%]	18.26% [17.05%-19.54%]	3.81% [3.31%-4.38%]
**Musculoskeletal issues: Arthritis (other or unspecified)**	10.53% [9.96%-11.13%]	6.89% [6.22%-7.62%]	13.92% [13.03%-14.86%]
**Musculoskeletal issues: Back or spine problems**	10.48% [9.88%-11.10%]	7.85% [7.09%-8.69%]	12.93% [12.04%-13.86%]
**Neurodevelopmental or neurobehavioral disorders: Attention Deficit Disorder (ADD) or Attention Deficit-Hyperactivity Disorder (ADHD)**	10.43% [9.76%-11.13%]	19.93% [18.66%-21.27%]	1.56% [1.25%-1.93%]
**Neurological disorders: Any other or unspecified**	9.20% [8.62%-9.81%]	10.43% [9.53%-11.39%]	8.06% [7.34%-8.84%]
**Cardiovascular system disorders: Any other or unspecified**	6.96% [6.48%-7.47%]	5.54% [4.93%-6.22%]	8.28% [7.57%-9.05%]
**Sensory or speech disorders: Deafness or hearing difficulty**	6.69% [6.20%-7.22%]	2.37% [1.95%-2.87%]	10.72% [9.88%-11.62%]
**Other: Any other conditions, including those not sufficiently specific to classify**	5.51% [5.05%-6.01%]	6.34% [5.61%-7.15%]	4.74% [4.19%-5.36%]
**Endocrine disorders: Diabetes**	5.48% [5.05%-5.94%]	4.62% [4.06%-5.25%]	6.28% [5.66%-6.97%]
**Sensory or speech disorders: Blindness or vision problems**	5.10% [4.65%-5.58%]	2.89% [2.41%-3.47%]	7.15% [6.45%-7.93%]

© 2026 Ne’eman A. *JAMA Health Forum*.

**Cardiovascular system disorders: High blood pressure**	4.38% [4.00%-4.79%]	3.41% [2.96%-3.93%]	5.28% [4.70%-5.93%]
**Neurodevelopmental or neurobehavioral disorders: Autism Spectrum Disorder and Asperger Syndrome**	3.89% [3.45%-4.38%]	7.28% [6.44%-8.23%]	0.72% [0.49%-1.07%]
**Mental or emotional disorders: Any other or unspecified**	3.46% [3.07%-3.89%]	6.28% [5.54%-7.12%]	0.82% [0.60%-1.13%]
**Mental or emotional disorders: Bipolar disorder**	3.22% [2.84%-3.64%]	5.87% [5.14%-6.69%]	0.75% [0.53%-1.06%]
**Mental or emotional disorders: Trauma and stressor-related disorders**	3.03% [2.66%-3.45%]	4.93% [4.26%-5.71%]	1.26% [0.97%-1.63%]
**Other: Pain (unspecified)**	2.78% [2.46%-3.15%]	3.26% [2.78%-3.83%]	2.33% [1.92%-2.84%]
**Respiratory disorders: Chronic Obstructive Pulmonary Disease (COPD)**	2.34% [2.05%-2.66%]	1.80% [1.46%-2.22%]	2.84% [2.40%-3.35%]
**Neurological disorders: Stroke or Brain Aneurysm**	2.16% [1.90%-2.46%]	2.73% [2.30%-3.24%]	1.63% [1.34%-1.98%]
**Cancer, tumor, cyst, or growth**	2.01% [1.76%-2.29%]	1.76% [1.43%-2.16%]	2.24% [1.88%-2.66%]
**Neurodevelopmental or neurobehavioral disorders: Learning disability**	1.96% [1.66%-2.32%]	3.31% [2.74%-3.99%]	0.71% [0.50%-1.01%]
**Immune system disorders**	1.71% [1.45%-2.03%]	2.00% [1.58%-2.53%]	1.45% [1.15%-1.82%]
**Respiratory disorders: Asthma**	1.71% [1.43%-2.04%]	1.60% [1.23%-2.08%]	1.81% [1.42%-2.30%]
**Digestive system disorders (including liver conditions, stomach problems)**	1.65% [1.39%-1.96%]	1.97% [1.58%-2.44%]	1.35% [1.02%-1.78%]
**Sensory or speech disorders: Any other or unspecified (including speech disorders, vestibular problems)**	1.63% [1.39%-1.91%]	1.41% [1.08%-1.85%]	1.84% [1.52%-2.22%]
**Musculoskeletal issues: Rheumatoid arthritis**	1.60% [1.36%-1.87%]	1.16% [0.86%-1.55%]	2.00% [1.66%-2.42%]

© 2026 Ne’eman A. *JAMA Health Forum*.

**Neurodevelopmental or neurobehavioral disorders: Any other and unspecified**	1.58% [1.32%-1.88%]	2.72% [2.24%-3.31%]	0.51% [0.33%-0.78%]
**Neurodevelopmental or neurobehavioral disorders: Intellectual disability (due to a congenital disorder, such as Down Syndrome, or some other cause)**	1.53% [1.27%-1.85%]	2.78% [2.27%-3.39%]	0.38% [0.23%-0.61%]
**Other: Aging**	1.49% [1.29%-1.72%]	1.11% [0.88%-1.40%]	1.85% [1.54%-2.22%]
**Respiratory disorders: Any other or unspecified**	1.48% [1.27%-1.73%]	1.20% [0.92%-1.56%]	1.74% [1.43%-2.11%]
**Genitourinary disorders**	1.38% [1.17%-1.63%]	1.18% [0.91%-1.55%]	1.56% [1.26%-1.93%]
**Neurological disorders: Dementia or Alzheimer's disease**	1.37% [1.19%-1.58%]	2.72% [2.35%-3.15%]	0.12% [0.06%-0.23%]
**Neurological disorders: Epilepsy or seizures**	1.10% [0.90%-1.35%]	1.79% [1.42%-2.25%]	0.46% [0.31%-0.69%]
**Endocrine disorders: Any other or unspecified**	0.84% [0.67%-1.05%]	0.63% [0.43%-0.93%]	1.03% [0.78%-1.35%]
**Other: COVID-19 or coronavirus**	0.35% [0.23%-0.52%]	0.47% [0.29%-0.76%]	0.23% [0.11%-0.48%]

© 2026 Ne'eman A. *JAMA Health Forum*.

Appendix Table S9: Most Common Diagnosis Groups, Cognitive and Non-Cognitive Disability Populations (2024 Data Only)

Rank	Condition	Prevalence
Five Most Common Disability Diagnoses - Overall		
1.	Musculoskeletal issues: Any other or unspecified	14.41% [13.57%-15.29%]
2.	Mental or emotional disorders: Anxiety or obsessive-compulsive disorders	12.50% [11.63%-13.42%]
3.	Mental or emotional disorders: Depression	12.16% [11.29%-13.09%]
4.	Neurodevelopmental or neurobehavioral disorders: Attention Deficit Disorder (ADD) or Attention Deficit-Hyperactivity Disorder (ADHD)	11.95% [11.08%-12.89%]
5.	Musculoskeletal issues: Back or spine problems	10.83% [10.08%-11.62%]
Five Most Common Disability Diagnoses - Cognitive Disability		
1.	Neurodevelopmental or neurobehavioral disorders: Attention Deficit Disorder (ADD) or Attention Deficit-Hyperactivity Disorder (ADHD)	22.88% [21.24%-24.62%]
2.	Mental or emotional disorders: Anxiety or obsessive-compulsive disorders	20.82% [19.26%-22.47%]
3.	Mental or emotional disorders: Depression	20.21% [18.65%-21.87%]
4.	Neurological disorders: Any other or unspecified	10.44% [9.37%-11.62%]
5.	Musculoskeletal issues: Any other or unspecified	9.25% [8.22%-10.38%]
Five Most Common Disability Diagnoses - No Cognitive Disability		
1.	Musculoskeletal issues: Any other or unspecified	19.22% [17.95%-20.55%]

© 2026 Ne'eman A. *JAMA Health Forum*.

**2.**	**Musculoskeletal issues: Arthritis (other or unspecified)**	**13.62%<br>[12.53%-14.79%]**
**3.**	**Musculoskeletal issues: Back or spine problems**	**12.66%<br>[11.60%-13.81%]**
**4.**	**Sensory or speech disorders: Deafness or hearing difficulty**	**11.34%<br>[10.36%-12.39%]**
**5.**	**Neurological disorders: Any other or unspecified**	**9.36%<br>[8.43%-10.40%]**

© 2026 Ne'eman A. *JAMA Health Forum*.

Appendix Table S10: Correlation Coefficients, By Cognitive Disability Status (2024 Data Only)

	Cognitive		Non-Cognitive	
	Spearman's	Pearson's	Spearman's	Pearson's
**Age Group**				
**5-21 vs 22-64**	0.596***	0.662***	0.370*	0.290
**22-64 vs 65+**	0.297	0.246	0.750***	0.878***
**5-21 vs. 65+**	-0.280	-0.183	0.014	0.084
**Sex**				
**Male vs Female**	0.824***	0.908***	0.849***	0.933***
**Race/Ethnicity**				
**White vs Black**	0.885***	0.894***	0.831***	0.863***
**White vs Hispanic**	0.882***	0.970***	0.826***	0.884***
**White vs Asian**	0.844***	0.943***	0.836***	0.888***
**White vs Other**	0.758***	0.917***	0.807***	0.911***
**Black vs Hispanic**	0.877***	0.926***	0.786***	0.937***
**Black vs Asian**	0.876***	0.915***	0.770***	0.856***
**Black vs Other**	0.708***	0.768***	0.803***	0.797***
**Asian vs Hispanic**	0.844***	0.957***	0.744***	0.888***
**Asian vs Other**	0.581***	0.840***	0.669***	0.772***
**Hispanic vs Other**	0.692***	0.882***	0.757***	0.832***
**Education***				
**No HS vs HS Diploma**	0.843***	0.802***	0.951***	0.940***
**No HS vs Some College**	0.762***	0.685***	0.921***	0.913***
**No HS vs BA+**	0.741***	0.521**	0.896***	0.807***

© 2026 Ne’eman A. *JAMA Health Forum*.

**HS Diploma vs Some College**	0.846***	0.931***	0.958***	0.989***
**HS Diploma vs BA+**	0.832***	0.817***	0.919***	0.940***
**Some College vs BA+**	0.882***	0.915***	0.940***	0.934***

© 2026 Ne’eman A. *JAMA Health Forum*.

Appendix Table S11: Most Common Diagnosis Groups By Age Group, Overall and by Cognitive Disability Status (2024 Data Only)

Age Group	Disability Group	Diagnosis	Prevalence
5-21	Overall	Neurodevelopmental or neurobehavioral disorders: Attention Deficit Disorder (ADD) or Attention Deficit-Hyperactivity Disorder (ADHD)	47.93% [43.39%-52.49%]
5-21	Overall	Neurodevelopmental or neurobehavioral disorders: Autism Spectrum Disorder and Asperger Syndrome	21.38% [17.87%-25.37%]
5-21	Overall	Mental or emotional disorders: Anxiety or obsessive-compulsive disorders	20.53% [16.98%-24.59%]
5-21	Overall	Mental or emotional disorders: Depression	10.14% [7.56%-13.49%]
5-21	Overall	Neurodevelopmental or neurobehavioral disorders: Any other and unspecified	7.80% [5.75%-10.50%]

© 2026 Ne’eman A. *JAMA Health Forum*.

**5-21**	**Cognitive Disability**	**Neurodevelopmental or neurobehavioral disorders: Attention Deficit Disorder (ADD) or Attention Deficit-Hyperactivity Disorder (ADHD)**	**53.86% [48.89%-58.76%]**
**5-21**	**Cognitive Disability**	**Neurodevelopmental or neurobehavioral disorders: Autism Spectrum Disorder and Asperger Syndrome**	**23.30% [19.37%-27.74%]**
**5-21**	**Cognitive Disability**	**Mental or emotional disorders: Anxiety or obsessive-compulsive disorders**	**22.12% [18.22%-26.59%]**

© 2026 Ne’eman A. *JAMA Health Forum*.

**5-21**	**Cognitive Disability**	**Mental or emotional disorders: Depression**	**10.39% [7.57%-14.11%]**
**5-21**	**Cognitive Disability**	**Neurodevelopmental or neurobehavioral disorders: Any other and unspecified**	**8.68% [6.36%-11.73%]**
**5-21**	**No Cognitive Disability**	**Neurodevelopmental or neurobehavioral disorders: Attention Deficit Disorder (ADD) or Attention Deficit-Hyperactivity Disorder (ADHD)**	**14.05% [7.54%-24.67%]**

© 2026 Ne’eman A. *JAMA Health Forum*.

5-21	No Cognitive Disability	Mental or emotional disorders: Anxiety or obsessive-compulsive disorders	11.44% [5.02%-23.99%]
5-21	No Cognitive Disability	Sensory or speech disorders: Blindness or vision problems	11.27% [5.79%-20.80%]
5-21	No Cognitive Disability	Neurodevelopmental or neurobehavioral disorders: Autism Spectrum Disorder and Asperger Syndrome	10.46% [4.95%-20.75%]

© 2026 Ne'eman A. *JAMA Health Forum*.

**5-21**	**No Cognitive Disability**	**Sensory or speech disorders: Deafness or hearing difficulty**	**9.68% [4.62%-19.18%]**
**22-64**	**Overall**	**Mental or emotional disorders: Anxiety or obsessive-compulsive disorders**	**18.27% [16.82%-19.81%]**
**22-64**	**Overall**	**Mental or emotional disorders: Depression**	**17.82% [16.36%-19.39%]**
**22-64**	**Overall**	**Neurodevelopmental or neurobehavioral disorders: Attention Deficit Disorder (ADD) or Attention Deficit-Hyperactivity Disorder (ADHD)**	**13.88% [12.54%-15.34%]**
**22-64**	**Overall**	**Musculoskeletal issues: Any other or unspecified**	**13.02% [11.81%-14.33%]**
**22-64**	**Overall**	**Musculoskeletal issues: Back or spine problems**	**11.55% [10.40%-12.82%]**
**22-64**	**Cognitive Disability**	**Mental or emotional disorders: Depression**	**26.99% [24.69%-29.41%]**
**22-64**	**Cognitive Disability**	**Mental or emotional disorders: Anxiety or obsessive-compulsive disorders**	**26.60% [24.35%-28.98%]**

© 2026 Ne’eman A. *JAMA Health Forum*.

22-64	Cognitive Disability	Neurodevelopmental or neurobehavioral disorders: Attention Deficit Disorder (ADD) or Attention Deficit-Hyperactivity Disorder (ADHD)	22.58% [20.39%-24.92%]
22-64	Cognitive Disability	Neurological disorders: Any other or unspecified	11.32% [9.80%-13.04%]
22-64	Cognitive Disability	Musculoskeletal issues: Back or spine problems	9.82% [8.37%-11.49%]
22-64	No Cognitive Disability	Musculoskeletal issues: Any other or unspecified	18.45% [16.42%-20.67%]
22-64	No Cognitive Disability	Musculoskeletal issues: Back or spine problems	13.74% [11.95%-15.75%]
22-64	No Cognitive Disability	Neurological disorders: Any other or unspecified	10.77% [9.14%-12.65%]
22-64	No Cognitive Disability	Musculoskeletal issues: Arthritis (other or unspecified)	10.10% [8.51%-11.96%]
22-64	No Cognitive Disability	Sensory or speech disorders: Deafness or hearing difficulty	8.42% [7.07%-10.00%]
65+	Overall	Musculoskeletal issues: Any other or unspecified	19.45% [18.11%-20.88%]
65+	Overall	Musculoskeletal issues: Arthritis (other or unspecified)	17.27% [16.01%-18.60%]
65+	Overall	Musculoskeletal issues: Back or spine problems	12.31% [11.23%-13.47%]

© 2026 Ne’eman A. *JAMA Health Forum*.

**65+**	**Overall**	**Cardiovascular system disorders: Any other or unspecified**	**11.73% [10.67%-12.88%]**
**65+**	**Overall**	**Sensory or speech disorders: Deafness or hearing difficulty**	**11.48% [10.42%-12.63%]**
**65+**	**Cognitive Disability**	**Musculoskeletal issues: Arthritis (other or unspecified)**	**17.44% [15.19%-19.94%]**
**65+**	**Cognitive Disability**	**Musculoskeletal issues: Any other or unspecified**	**16.63% [14.30%-19.25%]**
**65+**	**Cognitive Disability**	**Neurological disorders: Any other or unspecified**	**14.24% [12.22%-16.53%]**
**65+**	**Cognitive Disability**	**Neurological disorders: Dementia or Alzheimer's disease**	**12.87% [10.81%-15.26%]**
**65+**	**Cognitive Disability**	**Cardiovascular system disorders: Any other or unspecified**	**12.55% [10.62%-14.76%]**
**65+**	**No Cognitive Disability**	**Musculoskeletal issues: Any other or unspecified**	**20.62% [19.00%-22.34%]**
**65+**	**No Cognitive Disability**	**Musculoskeletal issues: Arthritis (other or unspecified)**	**17.20% [15.71%-18.80%]**
**65+**	**No Cognitive Disability**	**Sensory or speech disorders: Deafness or hearing difficulty**	**13.81% [12.46%-15.28%]**
**65+**	**No Cognitive Disability**	**Musculoskeletal issues: Back or spine problems**	**12.27% [11.01%-13.66%]**

© 2026 Ne'eman A. *JAMA Health Forum*.

**65+**	**No Cognitive Disability**	**Cardiovascular system disorders: Any other or unspecified**	**11.39% [10.15%-12.77%]**

© 2026 Ne’eman A. *JAMA Health Forum*.

Appendix Table S12: Prevalence of Diagnosis Groups, Overall and by ACS-6 Question (2024 Data Only)

Diagnosis	Overall	Cognitive	Self-Care	Mobility	Independent Living	Hearing	Vision
**Musculoskeletal issues: Any other or unspecified**	14.41% [13.57%-15.29%]	9.25% [8.22%-10.38%]	18.88% [16.58%-21.42%]	24.25% [22.77%-25.79%]	16.32% [14.71%-18.07%]	14.49% [12.96%-16.16%]	13.34% [11.61%-15.28%]
**Mental or emotional disorders: Anxiety or obsessive-compulsive disorders**	12.50% [11.63%-13.42%]	20.82% [19.26%-22.47%]	8.67% [7.08%-10.58%]	7.04% [6.17%-8.02%]	12.48% [10.94%-14.19%]	6.67% [5.54%-8.00%]	8.11% [6.59%-9.93%]
**Mental or emotional disorders: Depression**	12.16% [11.29%-13.09%]	20.21% [18.65%-21.87%]	10.40% [8.58%-12.56%]	8.91% [7.92%-10.01%]	13.15% [11.52%-14.97%]	7.29% [6.16%-8.60%]	7.34% [5.90%-9.09%]
**Neurodevelopmental or neurobehavioral disorders: Attention Deficit Disorder (ADD) or Attention Deficit-Hyperactivity Disorder (ADHD)**	11.95% [11.08%-12.89%]	22.88% [21.24%-24.62%]	5.33% [4.05%-6.98%]	3.27% [2.63%-4.06%]	5.60% [4.52%-6.92%]	2.96% [2.28%-3.85%]	4.60% [3.47%-6.08%]
**Musculoskeletal issues: Back or spine problems**	10.83% [10.08%-11.62%]	8.86% [7.85%-9.99%]	16.39% [14.13%-18.93%]	17.67% [16.35%-19.07%]	13.10% [11.60%-14.77%]	11.11% [9.75%-12.63%]	9.09% [7.64%-10.79%]
**Musculoskeletal issues: Arthritis (other or unspecified)**	10.63% [9.92%-11.39%]	7.42% [6.56%-8.38%]	15.18% [13.16%-17.46%]	17.71% [16.43%-19.06%]	12.09% [10.73%-13.60%]	11.71% [10.39%-13.18%]	10.16% [8.70%-11.83%]
**Neurological disorders: Any other or unspecified**	9.88% [9.16%-10.65%]	10.44% [9.37%-11.62%]	17.39% [15.21%-19.82%]	14.51% [13.31%-15.80%]	14.29% [12.78%-15.95%]	8.62% [7.43%-9.98%]	8.43% [7.05%-10.04%]
**Sensory or speech disorders: Deafness or hearing difficulty**	7.14% [6.56%-7.77%]	2.63% [2.09%-3.31%]	2.81% [1.89%-4.16%]	3.35% [2.76%-4.06%]	3.57% [2.80%-4.53%]	23.75% [21.92%-25.69%]	4.36% [3.43%-5.53%]

© 2026 Ne’eman A. *JAMA Health Forum*.

**Cardiovascular system disorders: Any other or unspecified**	6.92% [6.34%-7.53%]	4.95% [4.25%-5.76%]	10.15% [8.53%-12.02%]	10.89% [9.87%-12.00%]	10.13% [8.87%-11.54%]	8.53% [7.36%-9.86%]	8.28% [6.93%-9.86%]
**Other: Any other conditions, including those not sufficiently specific to classify**	5.94% [5.36%-6.59%]	6.04% [5.19%-7.01%]	7.88% [6.37%-9.71%]	8.33% [7.35%-9.42%]	7.47% [6.29%-8.84%]	3.90% [3.15%-4.81%]	6.42% [5.10%-8.04%]
**Endocrine disorders: Diabetes**	5.43% [4.90%-6.01%]	4.82% [4.07%-5.71%]	7.82% [6.41%-9.52%]	7.82% [6.96%-8.78%]	7.28% [6.19%-8.54%]	5.61% [4.70%-6.70%]	6.91% [5.67%-8.39%]
**Sensory or speech disorders: Blindness or vision problems**	4.73% [4.23%-5.28%]	2.32% [1.84%-2.92%]	2.96% [2.15%-4.07%]	3.28% [2.74%-3.91%]	4.95% [4.10%-5.95%]	4.18% [3.43%-5.09%]	20.20% [18.02%-22.58%]
**Cardiovascular system disorders: High blood pressure**	4.27% [3.80%-4.80%]	3.32% [2.75%-4.00%]	5.71% [4.43%-7.33%]	5.94% [5.13%-6.86%]	5.11% [4.27%-6.11%]	4.32% [3.56%-5.24%]	5.49% [4.28%-7.03%]
**Neurodevelopmental or neurobehavioral disorders: Autism Spectrum Disorder and Asperger Syndrome**	4.11% [3.58%-4.72%]	7.71% [6.69%-8.89%]	5.50% [4.16%-7.24%]	1.20% [0.86%-1.67%]	5.11% [4.07%-6.40%]	0.77% [0.45%-1.32%]	1.67% [1.05%-2.66%]
**Mental or emotional disorders: Any other or unspecified**	3.50% [3.03%-4.04%]	6.20% [5.31%-7.22%]	3.31% [2.24%-4.86%]	2.12% [1.64%-2.73%]	5.06% [4.02%-6.36%]	1.77% [1.23%-2.54%]	2.70% [1.93%-3.78%]
**Mental or emotional disorders: Trauma and stressor-related disorders**	2.99% [2.55%-3.50%]	5.29% [4.45%-6.28%]	3.07% [2.12%-4.43%]	2.49% [1.97%-3.15%]	3.75% [2.86%-4.90%]	2.82% [2.09%-3.79%]	1.26% [0.77%-2.05%]
**Mental or emotional disorders: Bipolar disorder**	2.97% [2.53%-3.48%]	5.24% [4.41%-6.22%]	3.23% [2.21%-4.71%]	2.31% [1.78%-3.01%]	3.69% [2.81%-4.82%]	1.74% [1.21%-2.51%]	2.34% [1.54%-3.54%]
**Other: Pain (unspecified)**	2.80% [2.40%-3.27%]	3.35% [2.75%-4.09%]	4.88% [3.66%-6.48%]	4.29% [3.58%-5.15%]	4.18% [3.32%-5.26%]	2.20% [1.62%-2.97%]	2.83% [2.01%-3.97%]

© 2026 Ne’eman A. *JAMA Health Forum*.

**Neurodevelopmental or neurobehavioral disorders: Learning disability**	2.20% [1.83%-2.64%]	3.14% [2.53%-3.91%]	0.90% [0.50%-1.61%]	0.92% [0.60%-1.41%]	1.03% [0.67%-1.60%]	1.22% [0.78%-1.91%]	1.88% [1.16%-3.03%]
**Respiratory disorders: Chronic Obstructive Pulmonary Disease (COPD)**	2.08% [1.78%-2.43%]	1.57% [1.21%-2.03%]	3.59% [2.65%-4.84%]	3.43% [2.88%-4.09%]	3.01% [2.37%-3.82%]	2.55% [2.00%-3.25%]	2.61% [1.85%-3.65%]
**Other: Aging**	1.98% [1.69%-2.31%]	1.48% [1.14%-1.92%]	2.79% [1.99%-3.91%]	2.74% [2.25%-3.33%]	2.88% [2.28%-3.64%]	3.24% [2.58%-4.06%]	2.49% [1.76%-3.52%]
**Cancer, tumor, cyst, or growth**	1.96% [1.67%-2.30%]	1.83% [1.44%-2.32%]	3.04% [2.19%-4.20%]	2.82% [2.32%-3.41%]	3.07% [2.42%-3.88%]	2.07% [1.57%-2.72%]	2.32% [1.64%-3.26%]
**Neurological disorders: Stroke or Brain Aneurysm**	1.96% [1.67%-2.30%]	2.31% [1.86%-2.87%]	4.57% [3.54%-5.88%]	3.02% [2.51%-3.63%]	3.92% [3.18%-4.82%]	2.14% [1.61%-2.84%]	2.89% [2.10%-3.96%]
**Respiratory disorders: Asthma**	1.70% [1.38%-2.10%]	1.91% [1.42%-2.56%]	2.20% [1.44%-3.35%]	2.08% [1.60%-2.69%]	1.83% [1.27%-2.63%]	0.84% [0.53%-1.33%]	2.24% [1.48%-3.39%]
**Sensory or speech disorders: Any other or unspecified (including speech disorders, vestibular problems)**	1.63% [1.35%-1.97%]	1.37% [1.01%-1.86%]	2.10% [1.45%-3.03%]	2.03% [1.62%-2.54%]	2.54% [1.90%-3.39%]	1.93% [1.41%-2.62%]	2.52% [1.72%-3.67%]
**Neurological disorders: Dementia or Alzheimer's disease**	1.60% [1.34%-1.91%]	3.20% [2.67%-3.82%]	5.06% [4.05%-6.31%]	2.30% [1.87%-2.84%]	4.52% [3.72%-5.48%]	2.48% [1.90%-3.25%]	1.68% [1.16%-2.42%]
**Neurodevelopmental or neurobehavioral disorders: Any other and unspecified**	1.56% [1.25%-1.96%]	2.76% [2.16%-3.52%]	2.66% [1.76%-4.00%]	1.09% [0.75%-1.59%]	2.22% [1.52%-3.21%]	0.67% [0.37%-1.22%]	1.83% [1.13%-2.96%]
**Genitourinary disorders**	1.56% [1.26%-1.92%]	1.18% [0.86%-1.61%]	3.11% [2.07%-4.65%]	2.19% [1.69%-2.83%]	2.44% [1.82%-3.27%]	1.84% [1.34%-2.52%]	3.03% [2.02%-4.51%]

© 2026 Ne'eman A. *JAMA Health Forum*.

**Musculoskeletal issues: Rheumatoid arthritis**	1.53% [1.27%-1.84%]	1.24% [0.92%-1.68%]	2.74% [1.96%-3.83%]	2.79% [2.28%-3.40%]	2.46% [1.90%-3.18%]	1.01% [0.68%-1.50%]	1.78% [1.21%-2.60%]
**Respiratory disorders: Any other or unspecified**	1.46% [1.20%-1.76%]	1.32% [0.99%-1.77%]	2.41% [1.64%-3.52%]	2.31% [1.86%-2.87%]	1.72% [1.25%-2.38%]	1.69% [1.21%-2.35%]	0.89% [0.56%-1.42%]
**Digestive system disorders (including liver conditions, stomach problems)**	1.44% [1.18%-1.75%]	1.37% [1.03%-1.82%]	1.88% [1.25%-2.82%]	1.75% [1.35%-2.25%]	1.82% [1.35%-2.45%]	1.23% [0.81%-1.84%]	1.56% [1.03%-2.33%]
**Immune system disorders**	1.40% [1.14%-1.72%]	1.50% [1.10%-2.03%]	2.84% [1.96%-4.11%]	1.90% [1.46%-2.47%]	2.24% [1.61%-3.09%]	1.03% [0.67%-1.57%]	1.48% [0.99%-2.22%]
**Neurodevelopmental or neurobehavioral disorders: Intellectual disability (due to a congenital disorder, such as Down Syndrome, or some other cause)**	1.17% [0.90%-1.53%]	2.33% [1.77%-3.05%]	3.38% [2.20%-5.16%]	0.86% [0.52%-1.41%]	2.78% [1.98%-3.90%]	0.65% [0.32%-1.31%]	1.24% [0.67%-2.30%]
**Neurological disorders: Epilepsy or seizures**	1.13% [0.88%-1.44%]	1.84% [1.39%-2.44%]	1.95% [1.21%-3.14%]	1.07% [0.74%-1.54%]	2.12% [1.48%-3.02%]	0.91% [0.56%-1.48%]	0.75% [0.40%-1.39%]
**Endocrine disorders: Any other or unspecified**	0.97% [0.75%-1.25%]	1.10% [0.75%-1.61%]	1.66% [0.97%-2.80%]	1.24% [0.89%-1.72%]	1.25% [0.80%-1.93%]	1.12% [0.75%-1.66%]	0.70% [0.39%-1.24%]
**Other: COVID-19 or coronavirus**	0.13% [0.07%-0.27%]	0.09% [0.03%-0.23%]	0.46% [0.16%-1.30%]	0.17% [0.07%-0.43%]	0.23% [0.08%-0.70%]	0.07% [0.01%-0.30%]	0.03% [0.00%-0.18%]

© 2026 Ne’eman A. *JAMA Health Forum*.

Appendix Table S13: Prevalence of Diagnoses, Overall and by ACS-6 Question - Age 5-21 Only

Diagnosis	Overall	Cognitive	Self-Care	Mobility	Independent Living	Hearing	Vision
**Neurodevelopmental or neurobehavioral disorders: Attention Deficit Disorder (ADD) or Attention Deficit-Hyperactivity Disorder (ADHD)**	40.14% [37.05%-43.30%]	46.64% [43.10%-50.22%]	22.09% [15.28%-30.83%]	21.96% [14.00%-32.73%]	32.87% [24.59%-42.37%]	18.85% [12.32%-27.73%]	15.25% [10.16%-22.27%]
**Mental or emotional disorders: Anxiety or obsessive-compulsive disorders**	22.48% [19.90%-25.30%]	25.40% [22.39%-28.65%]	16.19% [10.17%-24.79%]	12.71% [6.79%-22.54%]	30.40% [22.50%-39.65%]	3.88% [1.41%-10.22%]	14.80% [9.39%-22.56%]
**Neurodevelopmental or neurobehavioral disorders: Autism Spectrum Disorder and Asperger Syndrome**	17.24% [14.93%-19.82%]	19.30% [16.62%-22.29%]	44.23% [34.88%-54.01%]	25.46% [16.56%-37.02%]	31.19% [23.09%-40.63%]	13.80% [8.01%-22.72%]	11.55% [6.78%-19.00%]
**Mental or emotional disorders: Depression**	11.62% [9.74%-13.81%]	13.39% [11.16%-15.98%]	4.65% [2.19%-9.60%]	4.31% [1.72%-10.42%]	18.41% [12.29%-26.65%]	3.82% [1.43%-9.83%]	7.67% [4.52%-12.74%]
**Neurodevelopmental or neurobehavioral disorders: Learning disability**	6.46% [4.97%-8.35%]	7.53% [5.76%-9.80%]	3.48% [1.52%-7.79%]	4.45% [1.81%-10.53%]	3.79% [1.68%-8.35%]	2.68% [0.80%-8.61%]	4.37% [1.59%-11.43%]

© 2026 Ne'eman A. *JAMA Health Forum*.

**Neurodevelopmental or neurobehavioral disorders: Any other and unspecified**	6.13% [4.81%-7.79%]	7.00% [5.46%-8.93%]	14.75% [8.96%-23.32%]	13.21% [6.91%-23.81%]	10.15% [5.41%-18.26%]	6.95% [2.99%-15.33%]	7.83% [4.26%-13.96%]
**Other: Any other conditions, including those not sufficiently specific to classify**	5.02% [3.86%-6.50%]	4.91% [3.64%-6.60%]	11.89% [7.10%-19.26%]	18.05% [11.24%-27.70%]	5.98% [3.18%-10.95%]	7.90% [3.57%-16.57%]	5.07% [2.69%-9.34%]
**Mental or emotional disorders: Bipolar disorder**	3.47% [2.39%-5.01%]	4.25% [2.93%-6.12%]	7.67% [3.47%-16.07%]	6.90% [2.78%-16.09%]	5.46% [2.51%-11.46%]	2.34% [0.57%-9.11%]	3.45% [1.02%-11.08%]
**Sensory or speech disorders: Blindness or vision problems**	3.27% [2.31%-4.60%]	1.20% [0.63%-2.26%]	2.43% [0.86%-6.70%]	1.02% [0.14%-7.14%]	0.66% [0.09%-4.62%]	2.42% [0.76%-7.41%]	19.75% [13.74%-27.57%]
**Neurodevelopmental or neurobehavioral disorders: Intellectual disability (due to a congenital disorder, such as Down Syndrome, or some other cause)**	3.04% [2.15%-4.28%]	3.40% [2.37%-4.85%]	11.26% [6.24%-19.48%]	7.33% [2.76%-18.08%]	8.49% [4.44%-15.63%]	2.77% [1.00%-7.45%]	1.32% [0.40%-4.29%]
**Respiratory disorders: Asthma**	3.03% [2.05%-4.47%]	3.07% [1.96%-4.79%]	3.57% [0.98%-12.19%]	1.18% [0.28%-4.74%]	1.66% [0.51%-5.30%]	1.58% [0.38%-6.37%]	2.75% [1.05%-6.98%]
**Mental or emotional disorders: Any other or unspecified**	2.96% [2.08%-4.19%]	3.62% [2.54%-5.13%]	3.50% [1.38%-8.59%]	3.69% [1.27%-10.29%]	6.22% [3.18%-11.82%]	1.12% [0.26%-4.76%]	0.78% [0.18%-3.31%]

© 2026 Ne’eman A. *JAMA Health Forum*.

**Sensory or speech disorders: Deafness or hearing difficulty**	2.56% [1.76%-3.72%]	0.69% [0.29%-1.68%]	0.59% [0.12%-2.82%]	0.79% [0.16%-3.79%]	1.66% [0.38%-6.99%]	25.12% [17.20%-35.13%]	3.07% [1.17%-7.77%]
**Neurological disorders: Any other or unspecified**	2.09% [1.29%-3.39%]	1.93% [1.08%-3.43%]	5.21% [1.88%-13.60%]	7.18% [2.76%-17.43%]	4.70% [1.80%-11.69%]	1.02% [0.14%-7.08%]	3.02% [1.06%-8.25%]
**Sensory or speech disorders: Any other or unspecified (including speech disorders, vestibular problems)**	1.89% [1.13%-3.14%]	1.80% [0.97%-3.31%]	2.47% [0.94%-6.32%]	0.20% [0.03%-1.44%]	1.40% [0.41%-4.71%]	2.82% [0.90%-8.45%]	1.40% [0.32%-6.02%]
**Neurological disorders: Epilepsy or seizures**	1.77% [1.11%-2.80%]	1.74% [1.03%-2.90%]	6.01% [2.87%-12.14%]	6.14% [2.47%-14.45%]	7.21% [3.49%-14.33%]	4.36% [1.72%-10.61%]	3.74% [1.54%-8.81%]
**Musculoskeletal issues: Any other or unspecified**	1.61% [1.03%-2.52%]	0.80% [0.41%-1.56%]	4.40% [1.98%-9.49%]	9.81% [5.07%-18.14%]	1.91% [0.58%-6.07%]	4.89% [1.96%-11.67%]	2.40% [0.92%-6.11%]
**Immune system disorders**	1.38% [0.84%-2.26%]	1.32% [0.75%-2.34%]	1.45% [0.33%-6.14%]	5.67% [2.00%-15.05%]	2.04% [0.40%-9.66%]	1.26% [0.38%-4.08%]	0.87% [0.12%-6.01%]
**Mental or emotional disorders: Trauma and stressor-related disorders**	0.88% [0.43%-1.77%]	1.08% [0.53%-2.16%]	0.36% [0.05%-2.55%]	0.00% [0.00%-0.00%]	0.00% [0.00%-0.00%]	0.00% [0.00%-0.00%]	0.23% [0.03%-1.62%]
**Musculoskeletal issues: Back or spine problems**	0.83% [0.43%-1.62%]	0.81% [0.36%-1.79%]	1.84% [0.48%-6.79%]	4.32% [1.72%-10.42%]	1.45% [0.49%-4.21%]	2.24% [0.76%-6.40%]	0.12% [0.02%-0.86%]

© 2026 Ne’eman A. *JAMA Health Forum*.

**Digestive system disorders (including liver conditions, stomach problems)**	0.67% [0.33%-1.35%]	0.62% [0.27%-1.45%]	1.74% [0.61%-4.83%]	1.71% [0.50%-5.69%]	1.07% [0.32%-3.54%]	0.59% [0.08%-4.22%]	0.77% [0.19%-3.09%]
**Musculoskeletal issues: Arthritis (other or unspecified)**	0.41% [0.15%-1.09%]	0.25% [0.06%-0.98%]	0.00% [0.00%-0.00%]	1.12% [0.15%-7.75%]	0.00% [0.00%-0.00%]	2.27% [0.55%-8.85%]	0.00% [0.00%-0.00%]
**Cardiovascular system disorders: Any other or unspecified**	0.41% [0.15%-1.12%]	0.46% [0.16%-1.36%]	0.00% [0.00%-0.00%]	1.75% [0.34%-8.39%]	0.88% [0.12%-6.11%]	0.00% [0.00%-0.00%]	0.00% [0.00%-0.00%]
**Endocrine disorders: Diabetes**	0.38% [0.11%-1.30%]	0.19% [0.07%-0.55%]	0.00% [0.00%-0.00%]	0.00% [0.00%-0.00%]	0.35% [0.05%-2.54%]	2.40% [0.33%-15.36%]	1.84% [0.31%-10.31%]
**Musculoskeletal issues: Rheumatoid arthritis**	0.28% [0.08%-0.92%]	0.20% [0.04%-0.91%]	0.00% [0.00%-0.00%]	2.89% [0.71%-11.06%]	0.00% [0.00%-0.00%]	1.22% [0.17%-8.34%]	0.00% [0.00%-0.00%]
**Respiratory disorders: Any other or unspecified**	0.22% [0.07%-0.70%]	0.17% [0.03%-0.89%]	0.64% [0.14%-2.89%]	1.29% [0.38%-4.29%]	0.00% [0.00%-0.00%]	0.00% [0.00%-0.00%]	0.00% [0.00%-0.00%]
**Respiratory disorders: Chronic Obstructive Pulmonary Disease (COPD)**	0.21% [0.04%-1.08%]	0.26% [0.05%-1.32%]	1.98% [0.39%-9.54%]	2.65% [0.51%-12.60%]	0.34% [0.05%-2.40%]	0.00% [0.00%-0.00%]	0.00% [0.00%-0.00%]
**Other: Pain (unspecified)**	0.15% [0.04%-0.49%]	0.14% [0.03%-0.59%]	0.00% [0.00%-0.00%]	0.43% [0.06%-3.08%]	0.00% [0.00%-0.00%]	0.00% [0.00%-0.00%]	0.00% [0.00%-0.00%]

© 2026 Ne’eman A. *JAMA Health Forum*.

**Genitourinary disorders**	0.14% [0.04%-0.57%]	0.09% [0.01%-0.65%]	0.69% [0.09%-4.84%]	0.00% [0.00%-0.00%]	0.00% [0.00%-0.00%]	0.00% [0.00%-0.00%]	0.51% [0.07%-3.58%]
**Cancer, tumor, cyst, or growth**	0.12% [0.03%-0.40%]	0.14% [0.04%-0.48%]	0.63% [0.13%-3.07%]	0.85% [0.17%-4.13%]	0.55% [0.11%-2.65%]	0.00% [0.00%-0.00%]	0.00% [0.00%-0.00%]
**Other: COVID-19 or coronavirus**	0.06% [0.01%-0.41%]	0.07% [0.01%-0.50%]	0.00% [0.00%-0.00%]	0.00% [0.00%-0.00%]	0.00% [0.00%-0.00%]	0.00% [0.00%-0.00%]	0.00% [0.00%-0.00%]
**Endocrine disorders: Any other or unspecified**	0.05% [0.01%-0.37%]	0.06% [0.01%-0.46%]	0.49% [0.07%-3.46%]	0.65% [0.09%-4.66%]	0.42% [0.06%-2.99%]	0.00% [0.00%-0.00%]	0.00% [0.00%-0.00%]
**Cardiovascular system disorders: High blood pressure**	0.04% [0.01%-0.30%]	0.05% [0.01%-0.37%]	0.00% [0.00%-0.00%]	0.00% [0.00%-0.00%]	0.00% [0.00%-0.00%]	0.00% [0.00%-0.00%]	0.00% [0.00%-0.00%]
**Other: Aging**	0.00% [0.00%-0.00%]	0.00% [0.00%-0.00%]	0.00% [0.00%-0.00%]	0.00% [0.00%-0.00%]	0.00% [0.00%-0.00%]	0.00% [0.00%-0.00%]	0.00% [0.00%-0.00%]
**Neurological disorders: Stroke or Brain Aneurysm**	0.00% [0.00%-0.00%]	0.00% [0.00%-0.00%]	0.00% [0.00%-0.00%]	0.00% [0.00%-0.00%]	0.00% [0.00%-0.00%]	0.00% [0.00%-0.00%]	0.00% [0.00%-0.00%]
**Neurological disorders: Dementia or Alzheimer’s disease**	0.00% [0.00%-0.00%]	0.00% [0.00%-0.00%]	0.00% [0.00%-0.00%]	0.00% [0.00%-0.00%]	0.00% [0.00%-0.00%]	0.00% [0.00%-0.00%]	0.00% [0.00%-0.00%]

All cells column percentages

© 2026 Ne’eman A. *JAMA Health Forum*.

Appendix Table S14: Prevalence of Diagnoses, Overall and by ACS-6 Question - Age 22-64 Only

Diagnosis	Overall	Cognitive	Self-Care	Mobility	Independent Living	Hearing	Vision
**Mental or emotional disorders: Anxiety or obsessive-compulsive disorders**	15.64% [14.50%-16.85%]	23.73% [21.88%-25.69%]	11.53% [9.39%-14.08%]	10.49% [9.14%-12.01%]	19.25% [17.04%-21.67%]	11.26% [9.22%-13.68%]	11.06% [9.10%-13.39%]
**Mental or emotional disorders: Depression**	15.26% [14.12%-16.47%]	24.21% [22.32%-26.21%]	13.12% [10.76%-15.90%]	11.96% [10.54%-13.55%]	16.43% [14.35%-18.76%]	10.56% [8.60%-12.91%]	11.84% [9.69%-14.40%]
**Musculoskeletal issues: Any other or unspecified**	13.48% [12.45%-14.59%]	8.34% [7.21%-9.62%]	16.39% [13.64%-19.56%]	23.49% [21.59%-25.49%]	13.46% [11.61%-15.55%]	11.46% [9.43%-13.85%]	11.52% [9.45%-13.97%]
**Musculoskeletal issues: Back or spine problems**	11.57% [10.62%-12.59%]	9.46% [8.28%-10.78%]	18.43% [15.56%-21.69%]	19.29% [17.54%-21.16%]	13.22% [11.40%-15.29%]	12.53% [10.38%-15.05%]	8.33% [6.74%-10.26%]
**Neurological disorders: Any other or unspecified**	10.78% [9.84%-11.79%]	12.11% [10.75%-13.62%]	21.57% [18.38%-25.15%]	15.92% [14.25%-17.74%]	15.96% [13.90%-18.28%]	8.81% [7.07%-10.92%]	10.23% [8.23%-12.67%]
**Neurodevelopmental or neurobehavioral disorders: Attention Deficit Disorder (ADD) or Attention Deficit-Hyperactivity Disorder (ADHD)**	9.82% [8.91%-10.82%]	16.73% [15.12%-18.47%]	5.04% [3.70%-6.83%]	3.66% [2.92%-4.57%]	5.93% [4.83%-7.27%]	5.06% [3.83%-6.67%]	4.69% [3.51%-6.25%]
**Musculoskeletal issues: Arthritis (other or unspecified)**	7.75% [6.97%-8.61%]	5.32% [4.45%-6.36%]	9.94% [7.96%-12.35%]	13.82% [12.33%-15.46%]	8.28% [6.86%-9.96%]	6.14% [4.84%-7.77%]	5.72% [4.39%-7.42%]

© 2026 Ne’eman A. *JAMA Health Forum*.

**Other: Any other conditions, including those not sufficiently specific to classify**	6.29% [5.55%-7.12%]	7.39% [6.28%-8.68%]	8.11% [6.23%-10.49%]	8.01% [6.82%-9.39%]	7.58% [6.19%-9.25%]	3.65% [2.59%-5.13%]	4.99% [3.73%-6.63%]
**Cardiovascular system disorders: Any other or unspecified**	5.59% [4.89%-6.37%]	4.74% [3.91%-5.74%]	7.19% [5.41%-9.49%]	8.86% [7.57%-10.34%]	7.28% [5.87%-9.01%]	5.84% [4.44%-7.65%]	4.86% [3.67%-6.41%]
**Endocrine disorders: Diabetes**	5.52% [4.87%-6.25%]	4.57% [3.76%-5.54%]	6.96% [5.26%-9.15%]	8.22% [7.07%-9.54%]	7.12% [5.78%-8.75%]	4.06% [2.98%-5.51%]	8.03% [6.44%-9.98%]
**Sensory or speech disorders: Deafness or hearing difficulty**	5.50% [4.77%-6.33%]	1.78% [1.26%-2.50%]	2.07% [1.02%-4.15%]	1.35% [0.89%-2.06%]	1.39% [0.85%-2.27%]	24.42% [21.39%-27.72%]	1.35% [0.70%-2.59%]
**Mental or emotional disorders: Any other or unspecified**	5.30% [4.61%-6.09%]	8.98% [7.76%-10.37%]	5.22% [3.62%-7.48%]	3.73% [2.90%-4.77%]	7.43% [6.01%-9.15%]	4.19% [2.95%-5.92%]	5.03% [3.60%-6.99%]
**Mental or emotional disorders: Trauma and stressor-related disorders**	5.15% [4.45%-5.94%]	7.97% [6.79%-9.33%]	6.57% [4.64%-9.21%]	4.61% [3.70%-5.72%]	5.39% [4.27%-6.78%]	5.18% [3.84%-6.94%]	3.42% [2.30%-5.06%]
**Mental or emotional disorders: Bipolar disorder**	5.00% [4.35%-5.75%]	8.30% [7.15%-9.61%]	5.73% [3.93%-8.26%]	4.08% [3.19%-5.21%]	7.06% [5.63%-8.83%]	4.31% [3.13%-5.90%]	3.92% [2.59%-5.89%]
**Sensory or speech disorders: Blindness or vision problems**	4.99% [4.30%-5.79%]	2.83% [2.13%-3.75%]	4.64% [3.20%-6.67%]	2.96% [2.29%-3.82%]	4.56% [3.49%-5.94%]	2.05% [1.31%-3.18%]	20.39% [17.53%-23.57%]
**Other: Pain (unspecified)**	4.04% [3.46%-4.72%]	4.56% [3.76%-5.52%]	7.39% [5.48%-9.90%]	6.49% [5.39%-7.81%]	5.88% [4.63%-7.43%]	2.70% [1.76%-4.12%]	3.31% [2.31%-4.73%]

© 2026 Ne’eman A. *JAMA Health Forum*.

**Cardiovascular system disorders: High blood pressure**	3.91% [3.36%-4.53%]	2.89% [2.30%-3.62%]	3.31% [2.29%-4.78%]	4.85% [3.95%-5.94%]	2.97% [2.22%-3.97%]	3.48% [2.49%-4.84%]	4.62% [3.48%-6.10%]
**Neurodevelopmental or neurobehavioral disorders: Autism Spectrum Disorder and Asperger Syndrome**	3.17% [2.61%-3.83%]	5.32% [4.35%-6.49%]	3.26% [2.19%-4.83%]	1.37% [0.92%-2.03%]	5.93% [4.62%-7.58%]	1.60% [0.91%-2.82%]	1.55% [0.92%-2.63%]
**Digestive system disorders (including liver conditions, stomach problems)**	2.22% [1.77%-2.79%]	2.63% [2.00%-3.44%]	1.97% [1.15%-3.34%]	2.68% [2.05%-3.49%]	2.49% [1.73%-3.57%]	2.05% [1.33%-3.15%]	2.52% [1.42%-4.42%]
**Immune system disorders**	2.15% [1.71%-2.69%]	2.49% [1.84%-3.37%]	3.60% [2.31%-5.58%]	2.79% [2.10%-3.71%]	3.16% [2.22%-4.47%]	1.14% [0.59%-2.17%]	2.64% [1.64%-4.23%]
**Neurodevelopmental or neurobehavioral disorders: Intellectual disability (due to a congenital disorder, such as Down Syndrome, or some other cause)**	2.12% [1.68%-2.68%]	3.53% [2.75%-4.52%]	6.48% [4.50%-9.24%]	2.06% [1.45%-2.94%]	5.67% [4.35%-7.36%]	0.89% [0.48%-1.65%]	1.72% [0.98%-3.01%]
**Cancer, tumor, cyst, or growth**	2.03% [1.64%-2.51%]	1.82% [1.34%-2.47%]	3.67% [2.43%-5.49%]	2.69% [2.06%-3.49%]	2.96% [2.10%-4.14%]	1.90% [1.19%-3.02%]	2.41% [1.52%-3.78%]
**Neurodevelopmental or neurobehavioral disorders: Learning disability**	1.92% [1.51%-2.43%]	2.64% [1.99%-3.50%]	1.19% [0.47%-2.97%]	1.38% [0.91%-2.09%]	1.86% [1.23%-2.82%]	1.35% [0.81%-2.24%]	1.34% [0.76%-2.37%]

© 2026 Ne’eman A. *JAMA Health Forum*.

**Respiratory disorders: Chronic Obstructive Pulmonary Disease (COPD)**	1.87% [1.47%-2.38%]	1.66% [1.19%-2.29%]	3.23% [2.18%-4.76%]	3.09% [2.42%-3.93%]	2.95% [2.14%-4.05%]	2.14% [1.40%-3.25%]	2.52% [1.43%-4.39%]
**Musculoskeletal issues: Rheumatoid arthritis**	1.76% [1.37%-2.24%]	1.29% [0.84%-1.99%]	3.64% [2.44%-5.39%]	3.13% [2.43%-4.02%]	2.04% [1.40%-2.96%]	0.82% [0.39%-1.73%]	1.65% [0.99%-2.74%]
**Neurological disorders: Stroke or Brain Aneurysm**	1.69% [1.34%-2.14%]	2.24% [1.68%-2.98%]	3.75% [2.54%-5.50%]	2.66% [2.03%-3.47%]	2.49% [1.78%-3.47%]	1.26% [0.73%-2.16%]	2.05% [1.34%-3.13%]
**Respiratory disorders: Asthma**	1.60% [1.20%-2.12%]	1.18% [0.81%-1.71%]	2.34% [1.36%-4.02%]	1.89% [1.35%-2.64%]	1.63% [1.04%-2.57%]	1.49% [0.78%-2.82%]	2.18% [1.10%-4.29%]
**Neurological disorders: Epilepsy or seizures**	1.56% [1.23%-1.99%]	2.36% [1.79%-3.11%]	2.85% [1.80%-4.49%]	1.53% [1.08%-2.18%]	3.57% [2.64%-4.81%]	1.11% [0.64%-1.92%]	1.31% [0.78%-2.18%]
**Genitourinary disorders**	1.46% [1.12%-1.91%]	1.32% [0.89%-1.95%]	2.61% [1.58%-4.29%]	1.94% [1.38%-2.70%]	2.30% [1.51%-3.49%]	1.34% [0.77%-2.31%]	2.51% [1.65%-3.80%]
**Neurodevelopmental or neurobehavioral disorders: Any other and unspecified**	1.45% [1.11%-1.90%]	1.97% [1.42%-2.74%]	4.83% [3.29%-7.06%]	2.07% [1.47%-2.93%]	3.39% [2.43%-4.70%]	0.64% [0.27%-1.51%]	1.53% [0.82%-2.83%]
**Respiratory disorders: Any other or unspecified**	1.42% [1.11%-1.82%]	1.34% [0.94%-1.90%]	1.90% [1.09%-3.28%]	2.26% [1.70%-3.01%]	2.12% [1.46%-3.06%]	1.35% [0.77%-2.35%]	1.03% [0.59%-1.81%]

© 2026 Ne’eman A. *JAMA Health Forum*.

**Endocrine disorders: Any other or unspecified**	1.12% [0.83%-1.52%]	0.96% [0.61%-1.50%]	1.57% [0.84%-2.92%]	1.38% [0.92%-2.08%]	1.01% [0.56%-1.84%]	0.80% [0.33%-1.95%]	0.70% [0.33%-1.46%]
**Sensory or speech disorders: Any other or unspecified (including speech disorders, vestibular problems)**	1.00% [0.72%-1.39%]	0.75% [0.44%-1.27%]	0.68% [0.29%-1.56%]	1.32% [0.88%-2.00%]	1.71% [1.06%-2.73%]	1.21% [0.66%-2.21%]	0.49% [0.20%-1.15%]
**Other: COVID-19 or coronavirus**	0.52% [0.32%-0.85%]	0.60% [0.33%-1.09%]	0.95% [0.39%-2.32%]	0.84% [0.45%-1.58%]	0.83% [0.38%-1.77%]	0.24% [0.06%-0.90%]	0.18% [0.04%-0.90%]
**Other: Aging**	0.25% [0.12%-0.50%]	0.22% [0.08%-0.57%]	0.14% [0.03%-0.64%]	0.24% [0.07%-0.79%]	0.02% [0.00%-0.17%]	0.37% [0.10%-1.33%]	0.08% [0.01%-0.59%]
**Neurological disorders: Dementia or Alzheimer's disease**	0.20% [0.11%-0.36%]	0.30% [0.15%-0.59%]	0.51% [0.20%-1.32%]	0.30% [0.14%-0.64%]	0.37% [0.17%-0.80%]	0.37% [0.16%-0.82%]	0.16% [0.04%-0.63%]

All cells column percentages

© 2026 Ne'eman A. *JAMA Health Forum*.

Appendix Table S15: Prevalence of Diagnoses, Overall and by ACS-6 Question - Age 65+ Only

Diagnosis	Overall	Cognitive	Self-Care	Mobility	Independent Living	Hearing	Vision
**Musculoskeletal issues: Any other or unspecified**	18.78% [17.67%-19.95%]	16.64% [14.78%-18.67%]	22.55% [19.76%-25.62%]	24.17% [22.65%-25.75%]	18.62% [16.72%-20.68%]	14.80% [13.32%-16.42%]	15.61% [13.48%-18.00%]
**Musculoskeletal issues: Arthritis (other or unspecified)**	17.79% [16.74%-18.88%]	16.88% [15.09%-18.84%]	18.79% [16.42%-21.42%]	22.00% [20.60%-23.47%]	17.73% [15.95%-19.67%]	14.90% [13.48%-16.45%]	17.02% [14.89%-19.38%]
**Musculoskeletal issues: Back or spine problems**	12.51% [11.59%-13.50%]	11.02% [9.49%-12.76%]	15.29% [12.91%-18.00%]	15.40% [14.16%-16.73%]	12.72% [11.14%-14.49%]	10.10% [8.90%-11.45%]	10.85% [9.12%-12.86%]
**Cardiovascular system disorders: Any other or unspecified**	11.10% [10.27%-12.00%]	12.29% [10.75%-14.01%]	15.17% [12.89%-17.78%]	12.59% [11.49%-13.78%]	14.11% [12.45%-15.96%]	11.23% [9.98%-12.62%]	12.82% [10.97%-14.93%]
**Sensory or speech disorders: Deafness or hearing difficulty**	9.72% [8.93%-10.57%]	5.34% [4.26%-6.68%]	4.48% [3.28%-6.08%]	3.84% [3.24%-4.56%]	4.73% [3.80%-5.87%]	21.82% [20.12%-23.61%]	5.99% [4.76%-7.51%]
**Neurological disorders: Any other or unspecified**	9.69% [8.86%-10.59%]	14.81% [13.06%-16.75%]	12.03% [10.00%-14.42%]	11.09% [10.01%-12.27%]	12.01% [10.47%-13.75%]	8.26% [7.11%-9.57%]	8.64% [6.99%-10.63%]
**Endocrine disorders: Diabetes**	7.26% [6.56%-8.03%]	9.03% [7.68%-10.59%]	11.20% [9.16%-13.61%]	8.70% [7.75%-9.76%]	9.73% [8.34%-11.32%]	5.84% [4.94%-6.90%]	8.03% [6.55%-9.82%]
**Cardiovascular system disorders: High blood pressure**	6.56% [5.88%-7.31%]	7.87% [6.63%-9.33%]	8.92% [7.15%-11.09%]	7.12% [6.26%-8.09%]	8.35% [7.04%-9.87%]	5.17% [4.33%-6.17%]	8.01% [6.53%-9.80%]

© 2026 Ne’eman A. *JAMA Health Forum*.

**Sensory or speech disorders: Blindness or vision problems**	5.89% [5.27%-6.58%]	4.67% [3.70%-5.87%]	5.09% [3.85%-6.71%]	4.65% [3.96%-5.45%]	7.55% [6.36%-8.94%]	5.24% [4.41%-6.22%]	21.41% [19.08%-23.95%]
**Other: Any other conditions, including those not sufficiently specific to classify**	4.67% [4.12%-5.29%]	5.31% [4.35%-6.47%]	5.12% [3.93%-6.64%]	5.51% [4.79%-6.34%]	6.23% [5.12%-7.56%]	4.00% [3.27%-4.89%]	4.08% [3.09%-5.37%]
**Mental or emotional disorders: Depression**	4.64% [4.08%-5.26%]	9.44% [8.06%-11.03%]	6.03% [4.60%-7.85%]	4.74% [4.06%-5.54%]	5.43% [4.40%-6.68%]	4.01% [3.27%-4.91%]	4.78% [3.70%-6.15%]
**Respiratory disorders: Chronic Obstructive Pulmonary Disease (COPD)**	3.72% [3.21%-4.30%]	3.63% [2.81%-4.69%]	4.70% [3.54%-6.22%]	4.56% [3.89%-5.34%]	5.46% [4.43%-6.71%]	3.50% [2.82%-4.32%]	2.99% [2.20%-4.06%]
**Other: Aging**	3.66% [3.17%-4.22%]	4.23% [3.36%-5.32%]	5.23% [3.93%-6.93%]	3.89% [3.26%-4.63%]	5.26% [4.31%-6.42%]	4.39% [3.61%-5.33%]	3.93% [2.95%-5.22%]
**Neurological disorders: Stroke or Brain Aneurysm**	3.56% [3.06%-4.13%]	6.50% [5.30%-7.94%]	6.42% [4.99%-8.22%]	4.15% [3.48%-4.93%]	6.27% [5.15%-7.61%]	3.48% [2.74%-4.41%]	3.46% [2.50%-4.77%]
**Mental or emotional disorders: Anxiety or obsessive-compulsive disorders**	3.50% [3.02%-4.06%]	6.49% [5.30%-7.92%]	4.52% [3.36%-6.07%]	3.49% [2.91%-4.19%]	4.84% [3.89%-6.00%]	3.17% [2.50%-4.02%]	4.10% [3.05%-5.50%]
**Neurological disorders: Dementia or Alzheimer's disease**	3.40% [2.94%-3.93%]	10.87% [9.41%-12.52%]	10.06% [8.32%-12.11%]	3.76% [3.16%-4.47%]	9.02% [7.75%-10.48%]	3.12% [2.48%-3.92%]	3.99% [3.03%-5.22%]

© 2026 Ne'eman A. *JAMA Health Forum*.

**Cancer, tumor, cyst, or growth**	2.66% [2.27%-3.12%]	3.20% [2.44%-4.19%]	3.19% [2.28%-4.44%]	2.88% [2.37%-3.49%]	4.15% [3.31%-5.20%]	2.75% [2.18%-3.47%]	2.40% [1.68%-3.43%]
**Sensory or speech disorders: Any other or unspecified (including speech disorders, vestibular problems)**	2.37% [1.99%-2.82%]	2.57% [1.87%-3.51%]	3.65% [2.59%-5.14%]	3.05% [2.52%-3.69%]	3.23% [2.48%-4.19%]	2.55% [1.99%-3.27%]	2.85% [1.99%-4.05%]
**Other: Pain (unspecified)**	2.08% [1.72%-2.52%]	3.33% [2.49%-4.42%]	2.99% [2.11%-4.23%]	2.41% [1.95%-2.98%]	2.83% [2.15%-3.71%]	1.86% [1.37%-2.52%]	3.30% [2.36%-4.61%]
**Respiratory disorders: Any other or unspecified**	2.00% [1.64%-2.45%]	1.88% [1.28%-2.78%]	3.58% [2.47%-5.17%]	2.46% [1.95%-3.09%]	2.67% [1.95%-3.65%]	1.51% [1.07%-2.13%]	1.61% [1.01%-2.55%]
**Musculoskeletal issues: Rheumatoid arthritis**	1.86% [1.53%-2.27%]	1.78% [1.24%-2.54%]	2.87% [1.99%-4.11%]	2.30% [1.85%-2.85%]	2.64% [1.99%-3.49%]	1.35% [0.95%-1.90%]	2.38% [1.56%-3.62%]
**Genitourinary disorders**	1.72% [1.41%-2.09%]	1.93% [1.39%-2.67%]	3.02% [2.17%-4.19%]	1.93% [1.54%-2.42%]	2.35% [1.74%-3.16%]	1.42% [1.02%-1.98%]	1.84% [1.24%-2.73%]
**Respiratory disorders: Asthma**	1.37% [1.07%-1.76%]	1.14% [0.67%-1.92%]	1.69% [1.03%-2.75%]	1.75% [1.32%-2.30%]	1.63% [1.12%-2.35%]	0.90% [0.59%-1.39%]	1.08% [0.67%-1.76%]
**Immune system disorders**	1.27% [0.97%-1.66%]	1.54% [0.96%-2.47%]	1.68% [0.98%-2.86%]	1.45% [1.07%-1.96%]	1.53% [1.00%-2.32%]	1.07% [0.70%-1.63%]	1.48% [0.88%-2.49%]
**Digestive system disorders (including liver conditions,**	1.25% [0.97%-1.60%]	1.76% [1.24%-2.51%]	1.96% [1.25%-3.05%]	1.49% [1.12%-1.98%]	1.44% [0.99%-2.08%]	1.09% [0.72%-1.64%]	1.33% [0.83%-2.12%]

© 2026 Ne’eman A. *JAMA Health Forum*.

**stomach problems)**							
**Mental or emotional disorders: Any other or unspecified**	1.23% [0.93%-1.63%]	2.72% [1.97%-3.75%]	1.47% [0.76%-2.83%]	1.07% [0.72%-1.59%]	1.55% [1.04%-2.30%]	0.74% [0.45%-1.23%]	1.08% [0.63%-1.84%]
**Mental or emotional disorders: Trauma and stressor-related disorders**	1.04% [0.79%-1.37%]	1.75% [1.21%-2.54%]	1.19% [0.63%-2.25%]	1.00% [0.72%-1.40%]	0.91% [0.54%-1.52%]	1.26% [0.85%-1.87%]	0.89% [0.44%-1.81%]
**Mental or emotional disorders: Bipolar disorder**	0.79% [0.55%-1.14%]	1.89% [1.27%-2.80%]	1.15% [0.56%-2.33%]	0.79% [0.50%-1.26%]	0.85% [0.47%-1.52%]	0.77% [0.45%-1.31%]	0.84% [0.40%-1.74%]
**Endocrine disorders: Any other or unspecified**	0.75% [0.55%-1.01%]	0.44% [0.20%-0.95%]	0.74% [0.37%-1.47%]	0.94% [0.66%-1.32%]	0.65% [0.37%-1.13%]	0.86% [0.55%-1.32%]	0.70% [0.36%-1.35%]
**Neurodevelopmental or neurobehavioral disorders: Attention Deficit Disorder (ADD) or Attention Deficit-Hyperactivity Disorder (ADHD)**	0.54% [0.38%-0.79%]	1.38% [0.89%-2.13%]	0.54% [0.20%-1.41%]	0.40% [0.23%-0.70%]	0.36% [0.15%-0.89%]	0.26% [0.12%-0.57%]	0.44% [0.16%-1.20%]
**Neurodevelopmental or neurobehavioral disorders: Learning disability**	0.41% [0.26%-0.66%]	0.73% [0.37%-1.43%]	0.26% [0.08%-0.84%]	0.40% [0.22%-0.74%]	0.40% [0.18%-0.89%]	0.34% [0.15%-0.75%]	0.22% [0.07%-0.74%]

© 2026 Ne'eman A. *JAMA Health Forum*.

**Neurological disorders: Epilepsy or seizures**	0.26% [0.16%-0.43%]	0.53% [0.28%-0.99%]	0.19% [0.05%-0.73%]	0.29% [0.15%-0.53%]	0.45% [0.23%-0.86%]	0.12% [0.05%-0.30%]	0.04% [0.01%-0.27%]
**Neurodevelopmental or neurobehavioral disorders: Intellectual disability (due to a congenital disorder, such as Down Syndrome, or some other cause)**	0.22% [0.11%-0.43%]	0.45% [0.21%-0.97%]	0.52% [0.19%-1.40%]	0.29% [0.14%-0.62%]	0.38% [0.16%-0.87%]	0.20% [0.08%-0.51%]	0.13% [0.03%-0.51%]
**Other: COVID-19 or coronavirus**	0.22% [0.11%-0.43%]	0.57% [0.26%-1.28%]	0.88% [0.38%-2.01%]	0.29% [0.14%-0.62%]	0.55% [0.25%-1.19%]	0.14% [0.05%-0.37%]	0.31% [0.07%-1.44%]
**Neurodevelopmental or neurobehavioral disorders: Any other and unspecified**	0.11% [0.04%-0.26%]	0.28% [0.10%-0.81%]	0.45% [0.16%-1.28%]	0.17% [0.07%-0.40%]	0.30% [0.12%-0.77%]	0.11% [0.02%-0.55%]	0.32% [0.10%-1.04%]
**Neurodevelopmental or neurobehavioral disorders: Autism Spectrum Disorder and Asperger Syndrome**	0.04% [0.01%-0.15%]	0.12% [0.03%-0.51%]	0.00% [0.00%-0.00%]	0.02% [0.00%-0.16%]	0.04% [0.01%-0.32%]	0.03% [0.00%-0.24%]	0.07% [0.01%-0.48%]

All cells column percentages

© 2026 Ne’eman A. *JAMA Health Forum*.

Appendix Table S16: Prevalence of Diagnoses, Overall and by ACS-6 Question - White Only

Diagnosis	Overall	Cognitive	Self-Care	Mobility	Independent Living	Hearing	Vision
**Musculoskeletal issues: Any other or unspecified**	14.21% [13.37%-15.09%]	9.02% [8.02%-10.13%]	17.82% [15.54%-20.34%]	23.51% [22.06%-25.02%]	14.69% [13.14%-16.39%]	13.16% [11.81%-14.64%]	11.86% [10.22%-13.74%]
**Mental or emotional disorders: Anxiety or obsessive-compulsive disorders**	12.56% [11.67%-13.50%]	21.38% [19.76%-23.10%]	8.98% [7.33%-10.94%]	7.20% [6.32%-8.19%]	14.91% [13.19%-16.81%]	6.50% [5.46%-7.72%]	9.03% [7.41%-10.96%]
**Musculoskeletal issues: Back or spine problems**	11.23% [10.48%-12.03%]	8.85% [7.84%-9.98%]	17.73% [15.32%-20.43%]	18.02% [16.70%-19.42%]	13.42% [11.88%-15.12%]	10.84% [9.61%-12.20%]	9.57% [8.10%-11.28%]
**Mental or emotional disorders: Depression**	11.18% [10.36%-12.05%]	19.33% [17.81%-20.96%]	10.03% [8.24%-12.17%]	8.14% [7.21%-9.17%]	11.89% [10.37%-13.60%]	6.62% [5.60%-7.82%]	8.75% [7.19%-10.60%]
**Neurodevelopmental or neurobehavioral disorders: Attention Deficit Disorder (ADD) or Attention Deficit-Hyperactivity Disorder (ADHD)**	10.85% [10.02%-11.73%]	21.51% [19.89%-23.22%]	4.92% [3.65%-6.58%]	2.54% [2.02%-3.19%]	5.74% [4.64%-7.08%]	3.12% [2.40%-4.05%]	4.86% [3.71%-6.34%]
**Musculoskeletal issues: Arthritis (other or unspecified)**	10.74% [10.05%-11.47%]	7.00% [6.20%-7.90%]	13.83% [11.96%-15.94%]	17.56% [16.35%-18.85%]	11.90% [10.58%-13.35%]	11.15% [10.00%-12.42%]	9.77% [8.39%-11.34%]

© 2026 Ne’eman A. *JAMA Health Forum*.

**Neurological disorders: Any other or unspecified**	9.88% [9.15%-10.66%]	11.46% [10.29%-12.74%]	17.84% [15.34%-20.66%]	14.19% [12.95%-15.52%]	15.50% [13.79%-17.39%]	7.87% [6.82%-9.07%]	9.95% [8.27%-11.92%]
**Sensory or speech disorders: Deafness or hearing difficulty**	7.79% [7.18%-8.44%]	2.44% [1.96%-3.04%]	3.34% [2.38%-4.67%]	3.11% [2.59%-3.72%]	3.41% [2.69%-4.30%]	23.52% [21.80%-25.33%]	3.95% [3.05%-5.10%]
**Cardiovascular system disorders: Any other or unspecified**	7.10% [6.54%-7.71%]	5.66% [4.91%-6.51%]	10.37% [8.65%-12.38%]	10.55% [9.57%-11.61%]	10.05% [8.81%-11.45%]	9.14% [8.06%-10.35%]	8.61% [7.32%-10.11%]
**Other: Any other conditions, including those not sufficiently specific to classify**	5.57% [5.02%-6.18%]	6.56% [5.67%-7.56%]	7.06% [5.59%-8.89%]	7.29% [6.40%-8.30%]	7.42% [6.24%-8.80%]	3.57% [2.93%-4.34%]	4.86% [3.80%-6.18%]
**Sensory or speech disorders: Blindness or vision problems**	4.60% [4.11%-5.15%]	2.47% [1.97%-3.10%]	4.03% [3.00%-5.38%]	3.18% [2.67%-3.78%]	5.02% [4.16%-6.06%]	3.58% [2.96%-4.33%]	21.08% [18.82%-23.53%]
**Endocrine disorders: Diabetes**	4.43% [3.95%-4.96%]	3.92% [3.27%-4.69%]	6.69% [5.25%-8.49%]	6.65% [5.85%-7.55%]	6.12% [5.12%-7.30%]	4.28% [3.51%-5.21%]	6.14% [4.87%-7.70%]
**Mental or emotional disorders: Bipolar disorder**	3.36% [2.89%-3.89%]	6.38% [5.45%-7.46%]	3.95% [2.73%-5.69%]	2.28% [1.76%-2.96%]	4.43% [3.47%-5.65%]	2.39% [1.76%-3.24%]	3.01% [1.96%-4.61%]
**Mental or emotional disorders: Trauma and stressor-related disorders**	3.30% [2.84%-3.82%]	5.22% [4.39%-6.21%]	4.01% [2.87%-5.59%]	3.09% [2.49%-3.84%]	3.47% [2.68%-4.49%]	2.95% [2.25%-3.85%]	2.38% [1.61%-3.52%]
**Neurodevelopmental or neurobehavioral disorders: Autism Spectrum Disorder and Asperger**	3.30% [2.82%-3.85%]	6.46% [5.49%-7.58%]	4.03% [2.90%-5.59%]	1.05% [0.71%-1.54%]	4.39% [3.39%-5.66%]	1.14% [0.71%-1.84%]	1.62% [0.95%-2.76%]

© 2026 Ne’eman A. *JAMA Health Forum*.

Syndrome							
**Cardiovascular system disorders: High blood pressure**	3.17% [2.77%-3.64%]	2.22% [1.79%-2.76%]	3.53% [2.56%-4.86%]	4.09% [3.42%-4.87%]	3.15% [2.46%-4.02%]	3.73% [3.05%-4.56%]	3.37% [2.60%-4.37%]
**Other: Pain (unspecified)**	2.97% [2.54%-3.46%]	3.65% [3.00%-4.42%]	5.53% [4.09%-7.44%]	4.61% [3.85%-5.51%]	4.34% [3.42%-5.50%]	2.07% [1.53%-2.79%]	2.87% [2.11%-3.89%]
**Respiratory disorders: Chronic Obstructive Pulmonary Disease (COPD)**	2.81% [2.46%-3.22%]	2.18% [1.73%-2.76%]	4.52% [3.44%-5.92%]	4.66% [4.02%-5.40%]	4.51% [3.70%-5.49%]	3.16% [2.57%-3.89%]	2.70% [2.02%-3.60%]
**Mental or emotional disorders: Any other or unspecified**	2.81% [2.39%-3.29%]	5.34% [4.50%-6.32%]	3.26% [2.18%-4.86%]	2.21% [1.69%-2.89%]	4.12% [3.21%-5.26%]	1.64% [1.14%-2.34%]	2.83% [1.91%-4.17%]
**Cancer, tumor, cyst, or growth**	2.29% [1.97%-2.68%]	2.07% [1.62%-2.66%]	3.37% [2.42%-4.67%]	2.95% [2.45%-3.55%]	3.67% [2.92%-4.62%]	2.58% [2.03%-3.27%]	2.58% [1.79%-3.72%]
**Neurological disorders: Stroke or Brain Aneurysm**	2.13% [1.81%-2.49%]	2.73% [2.22%-3.37%]	4.11% [3.05%-5.51%]	3.28% [2.73%-3.94%]	3.77% [3.03%-4.69%]	2.30% [1.77%-2.97%]	2.87% [2.08%-3.96%]
**Neurodevelopmental or neurobehavioral disorders: Learning disability**	1.94% [1.58%-2.38%]	3.24% [2.56%-4.07%]	0.86% [0.40%-1.81%]	0.96% [0.65%-1.39%]	1.55% [1.03%-2.33%]	0.85% [0.54%-1.33%]	1.28% [0.67%-2.41%]
**Other: Aging**	1.86% [1.59%-2.19%]	1.40% [1.07%-1.84%]	2.97% [2.14%-4.11%]	2.62% [2.15%-3.20%]	2.81% [2.23%-3.54%]	3.22% [2.60%-3.98%]	2.14% [1.54%-2.97%]

© 2026 Ne’eman A. *JAMA Health Forum*.

**Immune system disorders**	1.79% [1.47%-2.18%]	2.12% [1.61%-2.80%]	2.95% [1.98%-4.39%]	2.48% [1.95%-3.15%]	2.73% [1.98%-3.76%]	1.04% [0.69%-1.57%]	1.92% [1.26%-2.89%]
**Digestive system disorders (including liver conditions, stomach problems)**	1.70% [1.39%-2.07%]	2.12% [1.62%-2.78%]	1.79% [1.17%-2.71%]	2.02% [1.57%-2.59%]	2.00% [1.42%-2.80%]	1.45% [1.03%-2.04%]	1.77% [1.17%-2.65%]
**Sensory or speech disorders: Any other or unspecified (including speech disorders, vestibular problems)**	1.65% [1.37%-1.99%]	1.47% [1.06%-2.03%]	2.40% [1.68%-3.42%]	2.25% [1.82%-2.78%]	2.44% [1.83%-3.26%]	2.17% [1.68%-2.79%]	1.80% [1.25%-2.60%]
**Musculoskeletal issues: Rheumatoid arthritis**	1.57% [1.29%-1.90%]	0.97% [0.69%-1.36%]	3.27% [2.30%-4.63%]	2.63% [2.12%-3.25%]	2.13% [1.58%-2.87%]	1.13% [0.76%-1.66%]	1.85% [1.22%-2.80%]
**Neurodevelopmental or neurobehavioral disorders: Intellectual disability (due to a congenital disorder, such as Down Syndrome, or some other cause)**	1.50% [1.20%-1.87%]	2.81% [2.22%-3.55%]	4.59% [3.26%-6.42%]	1.39% [0.97%-1.98%]	3.62% [2.73%-4.77%]	0.66% [0.40%-1.09%]	1.22% [0.73%-2.02%]
**Respiratory disorders: Any other or unspecified**	1.49% [1.24%-1.79%]	1.20% [0.88%-1.64%]	2.37% [1.61%-3.48%]	2.20% [1.77%-2.73%]	2.29% [1.73%-3.02%]	1.42% [1.00%-2.02%]	1.22% [0.79%-1.89%]

© 2026 Ne'eman A. *JAMA Health Forum*.

**Neurological disorders: Dementia or Alzheimer's disease**	1.42% [1.20%-1.69%]	2.94% [2.47%-3.50%]	4.79% [3.79%-6.04%]	2.01% [1.63%-2.48%]	4.29% [3.57%-5.16%]	1.97% [1.53%-2.54%]	2.00% [1.47%-2.72%]
**Neurodevelopmental or neurobehavioral disorders: Any other and unspecified**	1.35% [1.06%-1.70%]	2.35% [1.80%-3.05%]	3.53% [2.40%-5.15%]	1.15% [0.80%-1.67%]	2.38% [1.69%-3.35%]	0.32% [0.13%-0.83%]	1.37% [0.80%-2.34%]
**Respiratory disorders: Asthma**	1.34% [1.06%-1.68%]	1.40% [0.98%-1.99%]	1.42% [0.88%-2.30%]	1.26% [0.95%-1.68%]	1.28% [0.89%-1.85%]	1.01% [0.62%-1.64%]	1.71% [1.05%-2.78%]
**Neurological disorders: Epilepsy or seizures**	1.22% [0.96%-1.55%]	2.09% [1.59%-2.74%]	1.97% [1.21%-3.19%]	0.95% [0.66%-1.35%]	2.81% [2.07%-3.81%]	0.76% [0.48%-1.21%]	1.27% [0.74%-2.20%]
**Genitourinary disorders**	1.19% [0.95%-1.48%]	1.32% [0.95%-1.83%]	2.43% [1.69%-3.48%]	1.59% [1.23%-2.04%]	1.98% [1.40%-2.80%]	1.00% [0.70%-1.41%]	1.52% [0.99%-2.32%]
**Endocrine disorders: Any other or unspecified**	0.88% [0.67%-1.17%]	0.80% [0.51%-1.26%]	1.33% [0.73%-2.42%]	1.11% [0.78%-1.56%]	0.95% [0.58%-1.56%]	0.84% [0.50%-1.39%]	0.58% [0.29%-1.16%]
**Other: COVID-19 or coronavirus**	0.34% [0.21%-0.54%]	0.49% [0.27%-0.88%]	0.76% [0.33%-1.75%]	0.48% [0.25%-0.89%]	0.70% [0.34%-1.43%]	0.15% [0.06%-0.34%]	0.21% [0.06%-0.77%]

All cells column percentages

© 2026 Ne'eman A. *JAMA Health Forum*.

Appendix Table S17: Prevalence of Diagnoses, Overall and by ACS-6 Question - Black Only

Diagnosis	Overall	Cognitive	Self-Care	Mobility	Independent Living	Hearing	Vision
**Musculoskeletal issues: Any other or unspecified**	13.77% [11.75%-16.07%]	8.54% [6.38%-11.32%]	18.50% [13.19%-25.32%]	22.78% [19.38%-26.56%]	16.29% [12.43%-21.07%]	14.33% [9.60%-20.86%]	15.08% [10.75%-20.75%]
**Musculoskeletal issues: Arthritis (other or unspecified)**	12.14% [10.37%-14.16%]	6.10% [4.54%-8.15%]	12.92% [9.13%-17.96%]	20.72% [17.61%-24.21%]	12.67% [9.50%-16.72%]	12.59% [8.67%-17.93%]	11.80% [8.42%-16.28%]
**Musculoskeletal issues: Back or spine problems**	9.15% [7.58%-11.02%]	5.17% [3.61%-7.36%]	10.84% [7.48%-15.46%]	13.93% [11.47%-16.82%]	10.21% [7.44%-13.86%]	10.40% [6.23%-16.87%]	6.60% [4.28%-10.06%]
**Neurological disorders: Any other or unspecified**	8.38% [6.79%-10.31%]	7.82% [5.72%-10.60%]	13.19% [9.22%-18.53%]	11.83% [9.29%-14.95%]	9.79% [6.85%-13.80%]	7.44% [4.07%-13.24%]	5.48% [3.35%-8.85%]
**Mental or emotional disorders: Anxiety or obsessive-compulsive disorders**	8.20% [6.51%-10.29%]	12.79% [9.82%-16.49%]	6.61% [3.68%-11.58%]	5.79% [3.86%-8.59%]	9.41% [6.29%-13.86%]	5.21% [2.54%-10.41%]	6.13% [3.74%-9.89%]
**Mental or emotional disorders: Depression**	8.07% [6.32%-10.24%]	13.37% [10.21%-17.32%]	5.03% [2.85%-8.72%]	6.12% [4.15%-8.94%]	8.95% [5.80%-13.55%]	2.58% [0.90%-7.14%]	8.27% [4.85%-13.74%]
**Neurodevelopmental or neurobehavioral disorders: Attention Deficit Disorder (ADD) or Attention Deficit-**	7.66% [5.81%-10.03%]	14.33% [10.86%-18.67%]	2.88% [1.24%-6.55%]	0.52% [0.19%-1.47%]	2.41% [1.21%-4.75%]	0.98% [0.30%-3.16%]	2.24% [0.93%-5.31%]

© 2026 Ne’eman A. *JAMA Health Forum*.

**Hyperactivity Disorder (ADHD)**							
**Cardiovascular system disorders: Any other or unspecified**	7.37% [5.82%-9.28%]	5.62% [3.88%-8.07%]	9.73% [6.47%-14.38%]	11.51% [8.91%-14.75%]	11.55% [8.19%-16.06%]	6.44% [3.62%-11.21%]	6.66% [4.28%-10.23%]
**Sensory or speech disorders: Blindness or vision problems**	7.22% [5.69%-9.12%]	4.15% [2.45%-6.96%]	4.40% [2.57%-7.44%]	6.08% [4.47%-8.23%]	6.86% [4.73%-9.85%]	4.54% [2.45%-8.24%]	24.05% [18.67%-30.39%]
**Endocrine disorders: Diabetes**	6.72% [5.43%-8.28%]	6.02% [4.29%-8.38%]	9.05% [5.89%-13.68%]	9.58% [7.51%-12.14%]	9.38% [6.59%-13.17%]	4.67% [2.67%-8.03%]	7.49% [5.13%-10.82%]
**Cardiovascular system disorders: High blood pressure**	6.59% [5.42%-7.99%]	5.46% [4.00%-7.41%]	7.35% [4.88%-10.94%]	8.01% [6.31%-10.10%]	7.22% [5.20%-9.94%]	6.13% [3.82%-9.71%]	9.55% [6.85%-13.17%]
**Mental or emotional disorders: Any other or unspecified**	5.54% [4.17%-7.33%]	9.38% [6.92%-12.58%]	4.34% [2.02%-9.08%]	3.44% [2.13%-5.50%]	6.43% [4.08%-9.99%]	5.76% [2.77%-11.58%]	4.62% [2.39%-8.75%]
**Neurodevelopmental or neurobehavioral disorders: Autism Spectrum Disorder and Asperger Syndrome**	5.38% [3.78%-7.61%]	9.20% [6.32%-13.20%]	7.94% [4.24%-14.36%]	1.72% [0.73%-4.00%]	6.96% [4.03%-11.77%]	1.56% [0.37%-6.31%]	2.10% [0.74%-5.83%]
**Other: Any other conditions, including those not sufficiently specific to classify**	3.99% [2.94%-5.38%]	4.16% [2.65%-6.48%]	4.40% [2.52%-7.57%]	4.14% [2.87%-5.95%]	4.32% [2.80%-6.60%]	5.22% [2.58%-10.29%]	2.13% [1.07%-4.18%]

© 2026 Ne’eman A. *JAMA Health Forum*.

**Neurological disorders: Stroke or Brain Aneurysm**	3.52% [2.57%-4.81%]	4.67% [3.11%-6.98%]	7.31% [4.61%-11.40%]	5.31% [3.79%-7.40%]	6.32% [4.23%-9.35%]	4.91% [2.39%-9.79%]	3.25% [1.88%-5.55%]
**Respiratory disorders: Asthma**	3.25% [2.09%-5.02%]	2.34% [1.20%-4.53%]	4.38% [1.83%-10.10%]	2.98% [1.79%-4.90%]	2.15% [0.99%-4.63%]	1.03% [0.36%-2.91%]	4.60% [1.82%-11.14%]
**Sensory or speech disorders: Deafness or hearing difficulty**	3.04% [1.81%-5.06%]	1.16% [0.44%-3.07%]	1.33% [0.55%-3.18%]	0.90% [0.47%-1.72%]	0.66% [0.25%-1.74%]	18.38% [11.25%-28.58%]	1.57% [0.77%-3.19%]
**Neurodevelopmental or neurobehavioral disorders: Intellectual disability (due to a congenital disorder, such as Down Syndrome, or some other cause)**	2.74% [1.72%-4.34%]	5.17% [3.21%-8.21%]	6.60% [2.85%-14.53%]	1.57% [0.65%-3.73%]	5.44% [2.86%-10.11%]	0.75% [0.15%-3.57%]	1.18% [0.20%-6.67%]
**Respiratory disorders: Chronic Obstructive Pulmonary Disease (COPD)**	2.59% [1.55%-4.30%]	1.84% [0.90%-3.73%]	3.04% [1.40%-6.47%]	3.29% [2.01%-5.35%]	3.76% [1.97%-7.05%]	2.98% [1.14%-7.54%]	4.54% [1.78%-11.10%]
**Mental or emotional disorders: Bipolar disorder**	2.55% [1.57%-4.10%]	4.44% [2.67%-7.31%]	3.58% [1.04%-11.58%]	1.92% [0.80%-4.51%]	2.95% [1.23%-6.94%]	0.44% [0.07%-2.58%]	2.68% [0.89%-7.78%]
**Mental or emotional disorders: Trauma and stressor-related disorders**	2.26% [1.29%-3.93%]	4.23% [2.37%-7.46%]	4.59% [1.48%-13.39%]	1.51% [0.72%-3.13%]	1.53% [0.66%-3.50%]	0.37% [0.09%-1.53%]	1.67% [0.62%-4.45%]
**Neurodevelopmental or neurobehavioral disorders:**	1.85% [1.01%-3.37%]	3.26% [1.68%-6.21%]	1.99% [0.45%-8.32%]	1.24% [0.45%-3.37%]	0.59% [0.16%-2.16%]	0.68% [0.10%-4.76%]	0.46% [0.10%-2.21%]

© 2026 Ne’eman A. *JAMA Health Forum*.

**Learning disability**							
**Genitourinary disorders**	1.81% [1.20%-2.73%]	1.21% [0.58%-2.53%]	2.78% [1.06%-7.07%]	2.43% [1.49%-3.93%]	2.49% [1.24%-4.93%]	1.21% [0.42%-3.48%]	2.65% [1.45%-4.81%]
**Other: Pain (unspecified)**	1.76% [1.11%-2.78%]	1.61% [0.80%-3.22%]	1.81% [0.85%-3.83%]	2.60% [1.54%-4.34%]	2.15% [1.03%-4.43%]	3.60% [1.51%-8.32%]	1.79% [0.62%-5.04%]
**Musculoskeletal issues: Rheumatoid arthritis**	1.74% [1.20%-2.50%]	0.75% [0.38%-1.48%]	2.29% [1.15%-4.49%]	3.43% [2.37%-4.95%]	2.68% [1.61%-4.44%]	1.92% [0.81%-4.47%]	2.31% [1.15%-4.55%]
**Cancer, tumor, cyst, or growth**	1.66% [1.09%-2.54%]	1.20% [0.63%-2.26%]	3.74% [1.69%-8.10%]	2.59% [1.56%-4.25%]	2.61% [1.29%-5.21%]	0.85% [0.28%-2.53%]	1.85% [0.82%-4.13%]
**Neurodevelopmental or neurobehavioral disorders: Any other and unspecified**	1.41% [0.76%-2.60%]	2.22% [1.08%-4.52%]	3.30% [1.13%-9.27%]	1.50% [0.57%-3.87%]	2.35% [0.89%-6.01%]	1.91% [0.54%-6.57%]	1.93% [0.56%-6.43%]
**Immune system disorders**	1.40% [0.81%-2.41%]	1.70% [0.82%-3.50%]	0.73% [0.16%-3.23%]	0.96% [0.44%-2.08%]	1.25% [0.47%-3.24%]	1.85% [0.53%-6.30%]	1.16% [0.37%-3.58%]
**Neurological disorders: Dementia or Alzheimer's disease**	1.34% [0.84%-2.13%]	2.57% [1.60%-4.11%]	4.51% [2.59%-7.73%]	2.43% [1.47%-3.99%]	3.53% [2.21%-5.61%]	1.77% [0.71%-4.34%]	1.33% [0.58%-3.04%]

© 2026 Ne'eman A. *JAMA Health Forum*.

**Digestive system disorders (including liver conditions, stomach problems)**	1.31% [0.59%-2.85%]	1.00% [0.47%-2.10%]	0.55% [0.08%-3.82%]	1.43% [0.79%-2.57%]	0.58% [0.16%-2.10%]	0.94% [0.28%-3.12%]	3.22% [0.93%-10.54%]
**Sensory or speech disorders: Any other or unspecified (including speech disorders, vestibular problems)**	1.29% [0.68%-2.43%]	1.25% [0.43%-3.59%]	0.73% [0.17%-3.10%]	1.26% [0.67%-2.38%]	1.04% [0.46%-2.33%]	1.56% [0.48%-4.96%]	0.33% [0.07%-1.64%]
**Respiratory disorders: Any other or unspecified**	1.24% [0.73%-2.12%]	0.82% [0.36%-1.84%]	2.05% [0.72%-5.67%]	2.05% [1.14%-3.68%]	2.39% [1.11%-5.08%]	0.71% [0.17%-2.84%]	0.41% [0.13%-1.33%]
**Neurological disorders: Epilepsy or seizures**	1.03% [0.56%-1.86%]	1.45% [0.73%-2.86%]	2.73% [1.11%-6.58%]	1.24% [0.55%-2.79%]	1.95% [0.86%-4.36%]	0.00% [0.00%-0.00%]	0.93% [0.34%-2.51%]
**Other: Aging**	0.76% [0.40%-1.43%]	0.61% [0.27%-1.38%]	1.51% [0.63%-3.55%]	1.27% [0.62%-2.61%]	1.01% [0.48%-2.14%]	1.21% [0.43%-3.33%]	0.92% [0.35%-2.36%]
**Endocrine disorders: Any other or unspecified**	0.41% [0.17%-1.00%]	0.05% [0.01%-0.35%]	0.26% [0.06%-1.04%]	0.63% [0.22%-1.81%]	0.15% [0.04%-0.62%]	0.13% [0.02%-0.93%]	0.68% [0.17%-2.72%]
**Other: COVID-19 or coronavirus**	0.30% [0.09%-1.05%]	0.59% [0.17%-2.06%]	1.62% [0.46%-5.56%]	0.60% [0.17%-2.10%]	0.96% [0.27%-3.33%]	0.75% [0.10%-5.20%]	0.62% [0.09%-4.28%]

All cells column percentages

© 2026 Ne’eman A. *JAMA Health Forum*.

Appendix Table S18: Prevalence of Diagnoses, Overall and by ACS-6 Question - Hispanic Only

Diagnosis	Overall	Cognitive	Self-Care	Mobility	Independent Living	Hearing	Vision
**Musculoskeletal issues: Any other or unspecified**	12.90% [11.30%-14.69%]	6.77% [5.17%-8.84%]	18.61% [14.02%-24.30%]	23.96% [20.88%-27.35%]	14.66% [11.60%-18.36%]	13.14% [9.89%-17.26%]	10.38% [7.60%-14.01%]
**Mental or emotional disorders: Anxiety or obsessive-compulsive disorders**	11.66% [9.99%-13.57%]	18.76% [15.86%-22.06%]	8.87% [6.05%-12.81%]	6.68% [4.97%-8.93%]	10.70% [8.05%-14.10%]	6.28% [3.88%-9.99%]	9.51% [6.64%-13.45%]
**Mental or emotional disorders: Depression**	11.47% [9.79%-13.40%]	19.34% [16.37%-22.70%]	10.70% [7.42%-15.19%]	8.76% [6.82%-11.19%]	13.11% [9.99%-17.03%]	7.79% [5.18%-11.56%]	8.52% [6.04%-11.89%]
**Neurodevelopmental or neurobehavioral disorders: Attention Deficit Disorder (ADD) or Attention Deficit-Hyperactivity Disorder (ADHD)**	10.17% [8.61%-11.97%]	18.25% [15.42%-21.46%]	2.62% [1.26%-5.38%]	2.58% [1.51%-4.38%]	2.82% [1.67%-4.75%]	2.83% [1.59%-4.98%]	2.26% [1.08%-4.70%]
**Musculoskeletal issues: Back or spine problems**	9.08% [7.68%-10.70%]	7.02% [5.33%-9.20%]	14.94% [11.00%-19.97%]	15.90% [13.23%-19.00%]	11.52% [8.80%-14.95%]	9.24% [6.36%-13.24%]	7.46% [5.23%-10.53%]
**Musculoskeletal issues: Arthritis (other or unspecified)**	8.65% [7.38%-10.11%]	6.77% [5.29%-8.62%]	12.40% [9.05%-16.76%]	17.41% [14.72%-20.47%]	12.62% [9.79%-16.12%]	9.77% [7.35%-12.89%]	8.91% [6.42%-12.22%]
**Endocrine disorders: Diabetes**	8.16% [6.99%-9.50%]	6.01% [4.68%-7.70%]	11.33% [8.40%-15.13%]	12.66% [10.50%-15.18%]	13.06% [10.33%-16.38%]	8.24% [6.01%-11.19%]	10.13% [7.58%-13.41%]

© 2026 Ne’eman A. *JAMA Health Forum*.

**Neurological disorders: Any other or unspecified**	7.36% [6.09%-8.86%]	10.19% [8.09%-12.78%]	13.72% [9.67%-19.11%]	10.84% [8.71%-13.41%]	10.11% [7.48%-13.52%]	9.45% [6.64%-13.28%]	7.80% [5.13%-11.71%]
**Cardiovascular system disorders: High blood pressure**	7.03% [5.91%-8.34%]	5.56% [4.27%-7.22%]	10.29% [7.37%-14.18%]	11.58% [9.44%-14.12%]	10.27% [7.90%-13.25%]	5.52% [3.81%-7.92%]	8.29% [5.92%-11.49%]
**Cardiovascular system disorders: Any other or unspecified**	5.88% [4.81%-7.16%]	4.61% [3.45%-6.13%]	7.56% [4.79%-11.73%]	10.30% [8.13%-12.97%]	7.62% [5.38%-10.70%]	6.98% [4.87%-9.92%]	4.71% [3.06%-7.17%]
**Sensory or speech disorders: Deafness or hearing difficulty**	5.79% [4.63%-7.22%]	3.03% [1.84%-4.94%]	4.18% [1.76%-9.59%]	2.04% [1.10%-3.75%]	2.25% [1.12%-4.46%]	23.82% [19.35%-28.96%]	2.69% [1.26%-5.62%]
**Sensory or speech disorders: Blindness or vision problems**	5.54% [4.37%-6.99%]	3.08% [1.97%-4.78%]	6.29% [3.53%-10.95%]	3.88% [2.58%-5.81%]	6.15% [4.07%-9.19%]	5.29% [3.44%-8.05%]	18.54% [14.43%-23.50%]
**Other: Any other conditions, including those not sufficiently specific to classify**	5.40% [4.34%-6.69%]	5.69% [4.18%-7.72%]	8.04% [5.35%-11.92%]	6.88% [5.23%-9.00%]	6.60% [4.63%-9.32%]	4.42% [2.51%-7.66%]	5.22% [3.38%-8.00%]
**Mental or emotional disorders: Any other or unspecified**	4.88% [3.78%-6.28%]	8.26% [6.29%-10.77%]	4.34% [2.32%-7.99%]	2.51% [1.51%-4.16%]	6.84% [4.67%-9.91%]	2.70% [1.28%-5.58%]	3.08% [1.59%-5.90%]
**Neurodevelopmental or neurobehavioral disorders: Autism Spectrum Disorder and Asperger Syndrome**	4.66% [3.66%-5.93%]	8.23% [6.40%-10.54%]	6.81% [4.17%-10.95%]	1.76% [0.94%-3.27%]	5.18% [3.25%-8.17%]	2.00% [0.85%-4.64%]	2.73% [1.38%-5.31%]

© 2026 Ne’eman A. *JAMA Health Forum*.

**Mental or emotional disorders: Bipolar disorder**	2.75% [1.98%-3.81%]	4.65% [3.26%-6.58%]	2.72% [1.28%-5.69%]	3.46% [2.19%-5.41%]	3.71% [2.15%-6.32%]	2.34% [1.22%-4.44%]	2.30% [1.10%-4.77%]
**Other: Pain (unspecified)**	2.59% [1.92%-3.50%]	3.07% [2.09%-4.49%]	4.25% [2.38%-7.47%]	3.74% [2.58%-5.41%]	4.25% [2.69%-6.65%]	1.73% [0.84%-3.51%]	4.79% [2.93%-7.75%]
**Mental or emotional disorders: Trauma and stressor-related disorders**	2.34% [1.61%-3.40%]	3.85% [2.57%-5.73%]	1.75% [0.78%-3.88%]	1.28% [0.63%-2.61%]	2.38% [1.28%-4.40%]	2.88% [1.47%-5.57%]	1.45% [0.38%-5.36%]
**Neurodevelopmental or neurobehavioral disorders: Any other and unspecified**	2.17% [1.47%-3.20%]	3.55% [2.33%-5.38%]	4.82% [2.52%-9.05%]	1.95% [1.02%-3.71%]	3.16% [1.58%-6.23%]	1.80% [0.73%-4.33%]	1.96% [0.86%-4.44%]
**Neurodevelopmental or neurobehavioral disorders: Learning disability**	2.13% [1.41%-3.21%]	3.82% [2.47%-5.87%]	1.04% [0.37%-2.90%]	0.68% [0.30%-1.51%]	1.31% [0.61%-2.81%]	0.97% [0.30%-3.13%]	1.09% [0.32%-3.62%]
**Immune system disorders**	1.85% [1.18%-2.90%]	2.22% [1.19%-4.12%]	3.56% [1.57%-7.88%]	2.41% [1.35%-4.27%]	2.59% [1.22%-5.43%]	0.92% [0.36%-2.36%]	2.87% [1.19%-6.74%]
**Sensory or speech disorders: Any other or unspecified (including speech disorders, vestibular problems)**	1.77% [1.20%-2.60%]	1.04% [0.52%-2.07%]	2.16% [0.99%-4.63%]	2.87% [1.81%-4.53%]	3.40% [1.93%-5.93%]	1.91% [0.86%-4.20%]	1.98% [0.91%-4.25%]
**Genitourinary disorders**	1.76% [1.20%-2.57%]	0.67% [0.34%-1.31%]	2.95% [1.44%-5.93%]	2.85% [1.80%-4.50%]	2.48% [1.32%-4.59%]	2.84% [1.52%-5.23%]	2.76% [1.46%-5.15%]

© 2026 Ne'eman A. *JAMA Health Forum*.

**Digestive system disorders (including liver conditions, stomach problems)**	1.70% [1.15%-2.49%]	2.33% [1.42%-3.80%]	3.07% [1.69%-5.53%]	2.25% [1.44%-3.48%]	2.24% [1.29%-3.87%]	1.77% [0.78%-3.95%]	1.30% [0.57%-2.94%]
**Respiratory disorders: Asthma**	1.57% [1.04%-2.35%]	1.66% [0.95%-2.88%]	2.70% [1.25%-5.75%]	2.01% [1.18%-3.41%]	2.10% [1.07%-4.08%]	1.67% [0.77%-3.58%]	0.23% [0.06%-0.93%]
**Neurological disorders: Dementia or Alzheimer's disease**	1.47% [1.03%-2.10%]	2.65% [1.82%-3.84%]	4.48% [2.72%-7.27%]	2.47% [1.65%-3.70%]	4.59% [3.11%-6.72%]	2.30% [1.20%-4.37%]	1.64% [0.79%-3.39%]
**Respiratory disorders: Any other or unspecified**	1.39% [0.89%-2.16%]	1.44% [0.74%-2.78%]	2.85% [1.43%-5.58%]	2.26% [1.38%-3.67%]	1.82% [0.93%-3.54%]	1.39% [0.64%-2.99%]	1.14% [0.53%-2.44%]
**Musculoskeletal issues: Rheumatoid arthritis**	1.35% [0.89%-2.06%]	1.55% [0.89%-2.70%]	2.95% [1.45%-5.91%]	2.58% [1.59%-4.16%]	2.25% [1.24%-4.06%]	0.72% [0.33%-1.56%]	1.41% [0.54%-3.60%]
**Cancer, tumor, cyst, or growth**	1.33% [0.88%-2.01%]	1.18% [0.66%-2.08%]	2.13% [0.98%-4.59%]	2.04% [1.23%-3.37%]	2.83% [1.55%-5.09%]	1.03% [0.44%-2.39%]	1.18% [0.47%-2.95%]
**Neurodevelopmental or neurobehavioral disorders: Intellectual disability (due to a congenital disorder, such as Down Syndrome, or some other cause)**	1.16% [0.72%-1.88%]	1.65% [0.95%-2.88%]	2.68% [1.20%-5.86%]	1.01% [0.44%-2.33%]	2.57% [1.44%-4.53%]	0.26% [0.06%-1.04%]	0.79% [0.23%-2.65%]

© 2026 Ne'eman A. *JAMA Health Forum*.

**Neurological disorders: Stroke or Brain Aneurysm**	1.12% [0.75%-1.66%]	1.22% [0.73%-2.05%]	3.54% [2.08%-5.95%]	1.61% [0.97%-2.67%]	2.67% [1.65%-4.29%]	1.81% [1.01%-3.23%]	0.34% [0.11%-1.08%]
**Endocrine disorders: Any other or unspecified**	1.03% [0.63%-1.70%]	0.72% [0.32%-1.59%]	1.27% [0.53%-2.97%]	1.88% [1.08%-3.25%]	1.20% [0.46%-3.10%]	0.94% [0.35%-2.54%]	0.57% [0.17%-1.89%]
**Other: Aging**	0.76% [0.49%-1.17%]	0.63% [0.33%-1.23%]	1.25% [0.52%-3.00%]	0.96% [0.53%-1.76%]	1.30% [0.67%-2.51%]	1.16% [0.54%-2.46%]	1.12% [0.51%-2.44%]
**Neurological disorders: Epilepsy or seizures**	0.60% [0.33%-1.11%]	0.92% [0.44%-1.89%]	1.18% [0.43%-3.21%]	0.61% [0.26%-1.43%]	1.07% [0.46%-2.44%]	0.76% [0.19%-3.07%]	0.54% [0.16%-1.83%]
**Other: COVID-19 or coronavirus**	0.38% [0.12%-1.23%]	0.25% [0.06%-1.05%]	0.08% [0.01%-0.60%]	0.70% [0.17%-2.82%]	0.05% [0.01%-0.37%]	0.00% [0.00%-0.00%]	0.00% [0.00%-0.00%]
**Respiratory disorders: Chronic Obstructive Pulmonary Disease (COPD)**	0.36% [0.18%-0.72%]	0.28% [0.10%-0.73%]	1.21% [0.45%-3.22%]	0.64% [0.28%-1.43%]	0.94% [0.40%-2.17%]	0.35% [0.09%-1.42%]	0.41% [0.12%-1.34%]

All cells column percentages

© 2026 Ne’eman A. *JAMA Health Forum*.

Appendix Table S19: Prevalence of Diagnoses, Overall and by ACS-6 Question - Asian Only

Diagnosis	Overall	Cognitive	Self-Care	Mobility	Independent Living	Hearing	Vision
**Musculoskeletal issues: Any other or unspecified**	14.10% [10.74%-18.31%]	8.90% [5.45%-14.21%]	17.29% [10.05%-28.13%]	25.01% [18.97%-32.20%]	16.47% [10.84%-24.23%]	7.75% [3.92%-14.76%]	15.98% [8.73%-27.43%]
**Mental or emotional disorders: Anxiety or obsessive-compulsive disorders**	10.73% [7.52%-15.07%]	22.00% [15.34%-30.53%]	11.14% [4.76%-23.92%]	4.10% [1.73%-9.39%]	9.02% [4.58%-16.98%]	1.23% [0.30%-4.95%]	6.55% [2.77%-14.72%]
**Musculoskeletal issues: Arthritis (other or unspecified)**	9.46% [6.91%-12.84%]	7.41% [4.38%-12.27%]	5.95% [2.64%-12.85%]	14.51% [10.15%-20.34%]	8.28% [4.70%-14.20%]	7.97% [3.93%-15.50%]	8.90% [4.40%-17.17%]
**Musculoskeletal issues: Back or spine problems**	8.55% [6.08%-11.88%]	5.32% [2.77%-9.97%]	5.48% [2.13%-13.42%]	11.61% [7.71%-17.11%]	6.89% [3.59%-12.81%]	9.02% [4.56%-17.06%]	7.21% [3.39%-14.68%]
**Neurological disorders: Any other or unspecified**	8.37% [5.77%-11.99%]	9.15% [5.69%-14.39%]	13.50% [7.01%-24.40%]	10.51% [6.61%-16.29%]	11.97% [7.31%-18.99%]	7.44% [3.58%-14.82%]	10.24% [4.50%-21.65%]
**Endocrine disorders: Diabetes**	8.12% [5.38%-12.07%]	7.46% [4.30%-12.65%]	16.53% [7.67%-32.08%]	13.68% [8.48%-21.33%]	12.83% [6.87%-22.70%]	7.04% [3.46%-13.81%]	8.00% [3.58%-16.89%]
**Neurodevelopmental or neurobehavioral disorders: Attention Deficit Disorder (ADD) or Attention Deficit-Hyperactivity**	7.75% [5.28%-11.24%]	15.10% [9.95%-22.26%]	2.95% [0.69%-11.73%]	3.66% [1.41%-9.18%]	7.03% [3.09%-15.22%]	2.92% [1.06%-7.78%]	1.43% [0.34%-5.88%]

© 2026 Ne’eman A. *JAMA Health Forum*.

**Disorder (ADHD)**							
**Cardiovascular system disorders: High blood pressure**	7.74% [5.28%-11.22%]	9.09% [5.22%-15.37%]	12.44% [5.80%-24.70%]	8.49% [5.20%-13.57%]	11.12% [6.13%-19.35%]	6.60% [2.61%-15.71%]	7.78% [3.34%-17.06%]
**Cardiovascular system disorders: Any other or unspecified**	7.61% [5.18%-11.03%]	8.74% [5.02%-14.79%]	15.13% [7.61%-27.85%]	9.45% [5.88%-14.85%]	11.76% [6.62%-20.03%]	7.90% [3.78%-15.77%]	7.02% [2.74%-16.83%]
**Mental or emotional disorders: Depression**	7.10% [4.76%-10.46%]	12.76% [8.12%-19.48%]	5.02% [1.82%-13.10%]	6.82% [3.95%-11.52%]	8.43% [4.64%-14.83%]	4.39% [1.72%-10.73%]	4.51% [1.48%-12.94%]
**Sensory or speech disorders: Deafness or hearing difficulty**	6.31% [4.13%-9.51%]	3.06% [1.35%-6.81%]	1.58% [0.43%-5.64%]	3.31% [1.47%-7.26%]	4.79% [2.30%-9.70%]	20.14% [12.97%-29.91%]	2.99% [1.02%-8.45%]
**Other: Any other conditions, including those not sufficiently specific to classify**	6.16% [4.05%-9.25%]	5.68% [2.90%-10.86%]	9.66% [4.07%-21.25%]	10.07% [6.26%-15.79%]	5.85% [2.74%-12.05%]	3.19% [0.97%-9.98%]	6.53% [2.56%-15.65%]
**Sensory or speech disorders: Blindness or vision problems**	4.89% [2.98%-7.95%]	4.41% [2.29%-8.33%]	5.75% [2.22%-14.11%]	4.30% [2.03%-8.88%]	8.05% [3.95%-15.73%]	1.71% [0.59%-4.88%]	13.89% [7.28%-24.89%]
**Other: Pain (unspecified)**	2.34% [1.16%-4.68%]	2.46% [0.77%-7.54%]	3.62% [0.78%-15.25%]	3.39% [1.41%-7.92%]	2.94% [0.86%-9.56%]	0.00% [0.00%-0.00%]	0.49% [0.07%-3.58%]
**Genitourinary disorders**	2.30% [1.12%-4.67%]	1.31% [0.31%-5.34%]	5.55% [2.01%-14.46%]	1.67% [0.53%-5.11%]	5.96% [2.52%-13.47%]	1.40% [0.26%-7.18%]	3.67% [1.07%-11.85%]

© 2026 Ne’eman A. *JAMA Health Forum*.

**Neurodevelopmental or neurobehavioral disorders: Autism Spectrum Disorder and Asperger Syndrome**	2.20% [1.16%-4.13%]	5.01% [2.64%-9.31%]	2.34% [0.69%-7.63%]	0.36% [0.05%-2.56%]	0.84% [0.19%-3.63%]	0.00% [0.00%-0.00%]	0.67% [0.09%-4.81%]
**Other: Aging**	2.03% [0.98%-4.15%]	1.25% [0.39%-3.96%]	2.21% [0.65%-7.26%]	2.85% [1.14%-6.92%]	5.02% [2.33%-10.48%]	1.76% [0.50%-6.05%]	0.98% [0.13%-6.90%]
**Neurological disorders: Stroke or Brain Aneurysm**	1.94% [0.96%-3.88%]	1.57% [0.43%-5.53%]	1.74% [0.41%-7.08%]	2.80% [1.22%-6.30%]	1.47% [0.43%-4.88%]	3.53% [1.23%-9.71%]	1.87% [0.40%-8.35%]
**Mental or emotional disorders: Bipolar disorder**	1.82% [0.82%-4.00%]	3.62% [1.52%-8.38%]	2.79% [0.38%-17.84%]	0.50% [0.07%-3.52%]	2.13% [0.65%-6.74%]	0.89% [0.12%-6.20%]	0.00% [0.00%-0.00%]
**Cancer, tumor, cyst, or growth**	1.81% [0.94%-3.44%]	1.62% [0.60%-4.31%]	4.89% [1.95%-11.74%]	2.33% [1.02%-5.24%]	4.45% [2.16%-8.93%]	3.54% [1.45%-8.43%]	2.14% [0.51%-8.54%]
**Respiratory disorders: Chronic Obstructive Pulmonary Disease (COPD)**	1.67% [0.77%-3.57%]	1.77% [0.48%-6.39%]	1.06% [0.14%-7.47%]	2.28% [1.01%-5.08%]	3.65% [1.60%-8.08%]	2.21% [0.70%-6.79%]	0.59% [0.08%-4.25%]
**Respiratory disorders: Asthma**	1.52% [0.56%-4.10%]	0.21% [0.03%-1.48%]	0.00% [0.00%-0.00%]	1.88% [0.56%-6.12%]	0.32% [0.04%-2.27%]	2.18% [0.30%-14.15%]	0.00% [0.00%-0.00%]
**Digestive system disorders (including liver conditions, stomach problems)**	1.46% [0.71%-2.96%]	1.44% [0.44%-4.61%]	2.80% [0.87%-8.68%]	2.13% [0.94%-4.73%]	2.85% [1.16%-6.81%]	1.28% [0.31%-5.16%]	1.23% [0.30%-4.98%]
**Respiratory disorders: Any other or**	1.42% [0.66%-3.02%]	0.50% [0.07%-3.54%]	0.00% [0.00%-0.00%]	3.08% [1.43%-6.50%]	1.42% [0.35%-5.65%]	0.72% [0.10%-5.09%]	0.00% [0.00%-0.00%]

© 2026 Ne’eman A. *JAMA Health Forum*.

**unspecified**							
**Sensory or speech disorders: Any other or unspecified (including speech disorders, vestibular problems)**	1.41% [0.61%-3.20%]	1.91% [0.62%-5.69%]	3.00% [0.60%-13.68%]	1.62% [0.50%-5.10%]	2.70% [0.85%-8.19%]	2.20% [0.63%-7.40%]	0.00% [0.00%-0.00%]
**Neurodevelopmental or neurobehavioral disorders: Any other and unspecified**	1.41% [0.61%-3.20%]	3.20% [1.39%-7.21%]	2.50% [0.73%-8.17%]	0.91% [0.27%-3.00%]	0.90% [0.20%-3.97%]	0.28% [0.04%-2.06%]	0.67% [0.09%-4.81%]
**Neurological disorders: Dementia or Alzheimer’s disease**	1.40% [0.72%-2.67%]	3.17% [1.64%-6.07%]	6.90% [3.23%-14.13%]	2.17% [0.98%-4.74%]	4.85% [2.49%-9.21%]	1.75% [0.61%-4.89%]	0.43% [0.06%-3.16%]
**Neurological disorders: Epilepsy or seizures**	1.31% [0.41%-4.07%]	2.97% [0.93%-9.03%]	2.72% [0.62%-11.14%]	2.83% [0.89%-8.64%]	3.35% [0.76%-13.63%]	0.67% [0.09%-4.78%]	0.67% [0.09%-4.81%]
**Mental or emotional disorders: Trauma and stressor-related disorders**	1.26% [0.51%-3.10%]	2.45% [0.89%-6.58%]	1.76% [0.24%-11.91%]	1.05% [0.25%-4.31%]	1.69% [0.40%-6.83%]	0.72% [0.10%-5.09%]	1.21% [0.16%-8.38%]
**Neurodevelopmental or neurobehavioral disorders: Learning disability**	1.08% [0.42%-2.75%]	2.45% [0.94%-6.20%]	0.00% [0.00%-0.00%]	0.50% [0.07%-3.51%]	0.00% [0.00%-0.00%]	0.00% [0.00%-0.00%]	0.93% [0.13%-6.56%]

© 2026 Ne’eman A. *JAMA Health Forum*.

**Immune system disorders**	0.75% [0.18%-3.03%]	0.72% [0.10%-5.02%]	0.00% [0.00%-0.00%]	0.00% [0.00%-0.00%]	0.00% [0.00%-0.00%]	1.22% [0.17%-8.36%]	3.05% [0.73%-11.86%]
**Musculoskeletal issues: Rheumatoid arthritis**	0.65% [0.14%-2.96%]	0.00% [0.00%-0.00%]	0.00% [0.00%-0.00%]	1.42% [0.30%-6.32%]	0.00% [0.00%-0.00%]	0.00% [0.00%-0.00%]	0.00% [0.00%-0.00%]
**Endocrine disorders: Any other or unspecified**	0.58% [0.21%-1.56%]	0.37% [0.05%-2.62%]	0.99% [0.13%-7.04%]	0.00% [0.00%-0.00%]	0.00% [0.00%-0.00%]	0.00% [0.00%-0.00%]	1.01% [0.24%-4.11%]
**Mental or emotional disorders: Any other or unspecified**	0.42% [0.15%-1.17%]	0.95% [0.34%-2.66%]	0.00% [0.00%-0.00%]	0.18% [0.03%-1.31%]	0.29% [0.04%-2.10%]	0.38% [0.05%-2.75%]	0.00% [0.00%-0.00%]
**Other: COVID-19 or coronavirus**	0.39% [0.05%-2.75%]	0.89% [0.12%-6.14%]	2.31% [0.31%-15.17%]	0.85% [0.12%-5.87%]	1.36% [0.19%-9.20%]	0.00% [0.00%-0.00%]	0.00% [0.00%-0.00%]
**Neurodevelopmental or neurobehavioral disorders: Intellectual disability (due to a congenital disorder, such as Down Syndrome, or some other cause)**	0.30% [0.07%-1.28%]	0.69% [0.16%-2.92%]	1.15% [0.15%-8.09%]	0.00% [0.00%-0.00%]	0.68% [0.09%-4.78%]	0.00% [0.00%-0.00%]	0.00% [0.00%-0.00%]

All cells column percentages

© 2026 Ne’eman A. *JAMA Health Forum*.

Appendix Table S20: Prevalence of Diagnoses, Overall and by ACS-6 Question - Other Only

Diagnosis	Overall	Cognitive	Self-Care	Mobility	Independent Living	Hearing	Vision
**Mental or emotional disorders: Anxiety or obsessive-compulsive disorders**	18.48% [14.72%-22.94%]	26.86% [21.26%-33.32%]	14.33% [8.03%-24.27%]	10.18% [6.72%-15.14%]	24.32% [17.27%-33.09%]	6.85% [3.40%-13.32%]	8.83% [4.57%-16.37%]
**Neurodevelopmental or neurobehavioral disorders: Attention Deficit Disorder (ADD) or Attention Deficit-Hyperactivity Disorder (ADHD)**	16.52% [13.05%-20.68%]	24.81% [19.54%-30.95%]	14.54% [8.39%-23.99%]	5.17% [2.85%-9.18%]	11.57% [6.59%-19.55%]	2.65% [0.95%-7.18%]	8.19% [3.65%-17.37%]
**Mental or emotional disorders: Depression**	14.01% [10.78%-18.00%]	18.17% [13.53%-23.96%]	12.06% [6.95%-20.13%]	12.43% [8.59%-17.66%]	17.86% [11.99%-25.76%]	7.97% [4.14%-14.80%]	13.44% [7.99%-21.71%]
**Musculoskeletal issues: Any other or unspecified**	12.33% [9.59%-15.72%]	9.12% [6.25%-13.11%]	16.39% [10.31%-25.07%]	23.33% [17.90%-29.81%]	15.47% [10.52%-22.16%]	14.54% [9.05%-22.53%]	15.56% [9.19%-25.12%]
**Musculoskeletal issues: Arthritis (other or unspecified)**	10.33% [7.12%-14.75%]	7.74% [3.94%-14.64%]	6.72% [3.05%-14.17%]	14.38% [10.03%-20.19%]	9.20% [5.24%-15.63%]	14.28% [8.22%-23.66%]	7.44% [3.36%-15.69%]
**Musculoskeletal issues: Back or spine problems**	9.84% [7.37%-13.02%]	7.19% [4.65%-10.94%]	13.11% [7.68%-21.48%]	17.85% [13.14%-23.79%]	10.14% [6.30%-15.92%]	14.72% [8.87%-23.43%]	9.63% [5.06%-17.60%]

© 2026 Ne’eman A. *JAMA Health Forum*.

**Other: Any other conditions, including those not sufficiently specific to classify**	9.69% [6.38%-14.45%]	12.52% [7.70%-19.71%]	12.03% [5.67%-23.70%]	8.80% [5.24%-14.41%]	9.77% [5.49%-16.79%]	12.88% [6.70%-23.34%]	6.04% [2.15%-15.85%]
**Neurological disorders: Any other or unspecified**	9.02% [6.67%-12.09%]	6.08% [3.97%-9.21%]	11.70% [6.84%-19.31%]	13.05% [9.21%-18.17%]	11.61% [7.48%-17.58%]	11.46% [6.69%-18.95%]	12.06% [5.95%-22.92%]
**Neurodevelopmental or neurobehavioral disorders: Autism Spectrum Disorder and Asperger Syndrome**	7.35% [5.06%-10.56%]	10.70% [7.22%-15.57%]	14.84% [8.44%-24.79%]	1.49% [0.58%-3.79%]	7.70% [4.44%-13.04%]	1.43% [0.35%-5.66%]	1.08% [0.15%-7.55%]
**Cardiovascular system disorders: Any other or unspecified**	7.14% [5.04%-10.03%]	4.99% [2.80%-8.74%]	9.88% [4.92%-18.84%]	11.12% [7.52%-16.14%]	7.81% [4.14%-14.28%]	12.24% [6.94%-20.68%]	14.01% [7.17%-25.60%]
**Mental or emotional disorders: Bipolar disorder**	6.50% [4.13%-10.10%]	9.15% [5.78%-14.19%]	10.10% [4.29%-21.97%]	5.39% [2.42%-11.55%]	10.36% [5.37%-19.06%]	1.36% [0.33%-5.43%]	0.80% [0.11%-5.66%]
**Mental or emotional disorders: Trauma and stressor-related disorders**	5.81% [3.82%-8.73%]	8.77% [5.68%-13.30%]	5.62% [2.28%-13.20%]	5.79% [3.19%-10.27%]	7.35% [3.89%-13.46%]	2.48% [0.87%-6.82%]	4.41% [1.36%-13.41%]
**Endocrine disorders: Diabetes**	5.30% [3.47%-8.03%]	2.76% [1.25%-5.97%]	6.62% [3.08%-13.66%]	10.20% [6.51%-15.61%]	5.42% [2.82%-10.16%]	7.97% [3.71%-16.30%]	14.95% [7.95%-26.35%]
**Sensory or speech disorders: Blindness or vision problems**	4.69% [2.91%-7.46%]	3.05% [1.39%-6.57%]	6.53% [2.45%-16.27%]	5.43% [2.70%-10.62%]	6.55% [3.28%-12.68%]	7.58% [3.39%-16.07%]	21.69% [13.19%-33.56%]

© 2026 Ne'eman A. *JAMA Health Forum*.

**Sensory or speech disorders: Deafness or hearing difficulty**	4.44% [2.75%-7.08%]	2.02% [0.84%-4.78%]	0.00% [0.00%-0.00%]	2.08% [0.77%-5.55%]	2.40% [0.98%-5.79%]	18.89% [11.67%-29.11%]	6.75% [2.70%-15.89%]
**Other: Pain (unspecified)**	4.38% [2.82%-6.75%]	4.23% [2.36%-7.45%]	8.52% [4.22%-16.45%]	6.87% [4.18%-11.10%]	9.98% [6.00%-16.16%]	2.64% [0.73%-9.08%]	3.99% [1.45%-10.53%]
**Mental or emotional disorders: Any other or unspecified**	4.31% [2.60%-7.06%]	6.26% [3.66%-10.50%]	2.98% [0.60%-13.60%]	2.19% [0.87%-5.44%]	6.37% [2.97%-13.12%]	1.84% [0.42%-7.61%]	3.08% [1.10%-8.32%]
**Neurodevelopmental or neurobehavioral disorders: Any other and unspecified**	3.83% [2.24%-6.49%]	5.74% [3.29%-9.84%]	7.10% [2.79%-16.94%]	2.29% [0.83%-6.13%]	2.61% [0.92%-7.17%]	0.00% [0.00%-0.00%]	3.44% [0.84%-13.03%]
**Respiratory disorders: Asthma**	3.61% [2.03%-6.35%]	2.80% [1.17%-6.57%]	4.87% [1.39%-15.67%]	6.46% [3.33%-12.18%]	4.53% [1.72%-11.37%]	1.66% [0.41%-6.56%]	1.62% [0.37%-6.70%]
**Musculoskeletal issues: Rheumatoid arthritis**	3.60% [1.52%-8.29%]	4.20% [1.38%-12.06%]	3.88% [1.30%-11.02%]	2.83% [1.31%-6.04%]	2.77% [0.97%-7.69%]	2.91% [0.63%-12.37%]	3.13% [0.60%-14.88%]
**Cardiovascular system disorders: High blood pressure**	3.03% [1.87%-4.88%]	1.50% [0.59%-3.74%]	1.31% [0.32%-5.26%]	5.73% [3.35%-9.63%]	3.75% [1.69%-8.11%]	6.19% [2.90%-12.71%]	7.35% [3.26%-15.72%]
**Neurodevelopmental or neurobehavioral disorders: Learning disability**	2.93% [1.67%-5.07%]	3.10% [1.58%-5.99%]	0.40% [0.05%-2.88%]	1.17% [0.28%-4.79%]	2.10% [0.59%-7.13%]	0.77% [0.19%-3.13%]	3.12% [1.06%-8.85%]
**Neurological disorders: Stroke or Brain Aneurysm**	2.83% [1.53%-5.20%]	3.43% [1.62%-7.12%]	7.72% [3.24%-17.27%]	5.30% [2.64%-10.36%]	5.98% [2.77%-12.43%]	3.20% [0.99%-9.83%]	4.26% [1.42%-12.07%]

© 2026 Ne’eman A. *JAMA Health Forum*.

**Respiratory disorders: Any other or unspecified**	2.54% [1.20%-5.28%]	1.92% [0.66%-5.48%]	6.42% [2.44%-15.85%]	5.80% [2.77%-11.76%]	2.97% [0.87%-9.72%]	2.87% [0.90%-8.74%]	5.69% [1.67%-17.66%]
**Respiratory disorders: Chronic Obstructive Pulmonary Disease (COPD)**	2.45% [1.42%-4.18%]	2.27% [1.15%-4.41%]	6.99% [3.35%-14.01%]	5.36% [3.06%-9.21%]	5.68% [3.01%-10.46%]	5.91% [2.94%-11.53%]	3.91% [1.45%-10.08%]
**Sensory or speech disorders: Any other or unspecified (including speech disorders, vestibular problems)**	2.10% [1.04%-4.19%]	2.21% [0.91%-5.25%]	1.74% [0.41%-7.13%]	2.47% [1.00%-5.99%]	1.10% [0.26%-4.50%]	1.30% [0.18%-8.87%]	1.59% [0.22%-10.75%]
**Digestive system disorders (including liver conditions, stomach problems)**	1.95% [0.85%-4.41%]	1.71% [0.79%-3.67%]	3.59% [0.61%-18.40%]	3.99% [1.63%-9.44%]	3.97% [1.28%-11.62%]	0.71% [0.10%-5.04%]	1.87% [0.44%-7.65%]
**Immune system disorders**	1.80% [0.93%-3.45%]	1.37% [0.54%-3.42%]	2.20% [0.65%-7.17%]	2.02% [0.77%-5.20%]	2.21% [0.74%-6.41%]	1.43% [0.35%-5.67%]	1.11% [0.27%-4.46%]
**Cancer, tumor, cyst, or growth**	1.32% [0.60%-2.90%]	1.46% [0.52%-4.03%]	1.65% [0.48%-5.50%]	2.72% [1.15%-6.27%]	1.46% [0.52%-4.07%]	3.51% [1.03%-11.22%]	2.83% [0.85%-8.96%]
**Neurological disorders: Epilepsy or seizures**	1.26% [0.46%-3.40%]	1.22% [0.30%-4.80%]	2.37% [0.33%-15.29%]	0.72% [0.16%-3.22%]	2.62% [0.70%-9.30%]	0.00% [0.00%-0.00%]	0.00% [0.00%-0.00%]
**Endocrine disorders: Any other or unspecified**	0.89% [0.36%-2.18%]	0.00% [0.00%-0.00%]	0.64% [0.09%-4.60%]	1.75% [0.64%-4.68%]	0.42% [0.06%-2.96%]	1.63% [0.38%-6.78%]	1.28% [0.17%-8.83%]

© 2026 Ne’eman A. *JAMA Health Forum*.

**Neurodevelopmental or neurobehavioral disorders: Intellectual disability (due to a congenital disorder, such as Down Syndrome, or some other cause)**	0.75% [0.18%-3.03%]	0.96% [0.17%-5.05%]	2.79% [0.50%-13.99%]	0.21% [0.03%-1.47%]	2.26% [0.55%-8.82%]	0.00% [0.00%-0.00%]	0.00% [0.00%-0.00%]
**Genitourinary disorders**	0.70% [0.16%-2.96%]	1.11% [0.26%-4.68%]	1.08% [0.15%-7.51%]	1.59% [0.37%-6.63%]	0.00% [0.00%-0.00%]	3.24% [0.74%-13.00%]	2.64% [0.36%-16.81%]
**Other: COVID-19 or coronavirus**	0.43% [0.12%-1.48%]	0.43% [0.09%-2.14%]	0.99% [0.21%-4.62%]	0.49% [0.10%-2.26%]	0.64% [0.13%-2.97%]	0.26% [0.04%-1.88%]	0.00% [0.00%-0.00%]
**Other: Aging**	0.23% [0.03%-1.61%]	0.36% [0.05%-2.56%]	0.00% [0.00%-0.00%]	0.00% [0.00%-0.00%]	0.00% [0.00%-0.00%]	1.06% [0.14%-7.34%]	0.00% [0.00%-0.00%]
**Neurological disorders: Dementia or Alzheimer's disease**	0.11% [0.02%-0.80%]	0.18% [0.02%-1.29%]	0.00% [0.00%-0.00%]	0.26% [0.04%-1.84%]	0.34% [0.05%-2.43%]	0.53% [0.07%-3.76%]	0.00% [0.00%-0.00%]

All cells column percentages

© 2026 Ne'eman A. *JAMA Health Forum*.

Appendix Table S21: Prevalence of Diagnoses, Overall and by ACS-6 Question - Male Only

Diagnosis	Overall	Cognitive	Self-Care	Mobility	Independent Living	Hearing	Vision
**Neurodevelopmental or neurobehavioral disorders: Attention Deficit Disorder (ADD) or Attention Deficit-Hyperactivity Disorder (ADHD)**	12.49% [11.46%-13.59%]	23.53% [21.61%-25.57%]	5.40% [3.87%-7.47%]	2.14% [1.57%-2.90%]	6.71% [5.27%-8.52%]	3.18% [2.39%-4.23%]	5.55% [4.12%-7.44%]
**Musculoskeletal issues: Any other or unspecified**	12.32% [11.40%-13.31%]	7.02% [6.04%-8.15%]	16.34% [13.82%-19.21%]	22.93% [21.11%-24.87%]	12.69% [10.89%-14.74%]	11.89% [10.40%-13.55%]	11.25% [9.25%-13.63%]
**Musculoskeletal issues: Back or spine problems**	10.23% [9.36%-11.16%]	7.58% [6.50%-8.83%]	17.46% [14.64%-20.68%]	18.83% [17.08%-20.72%]	11.55% [9.74%-13.65%]	10.87% [9.41%-12.52%]	9.19% [7.45%-11.28%]
**Mental or emotional disorders: Anxiety or obsessive-compulsive disorders**	9.28% [8.40%-10.25%]	16.09% [14.46%-17.86%]	7.74% [5.93%-10.04%]	5.50% [4.51%-6.68%]	11.61% [9.72%-13.81%]	5.00% [3.96%-6.29%]	5.93% [4.55%-7.70%]
**Mental or emotional disorders: Depression**	8.92% [8.03%-9.90%]	15.36% [13.72%-17.16%]	7.95% [6.08%-10.33%]	6.66% [5.59%-7.91%]	9.97% [8.24%-12.01%]	5.11% [4.09%-6.37%]	7.42% [5.60%-9.75%]
**Neurological disorders: Any other or unspecified**	8.36% [7.59%-9.20%]	9.40% [8.22%-10.72%]	15.16% [12.67%-18.04%]	14.08% [12.60%-15.71%]	13.74% [11.83%-15.90%]	8.33% [7.07%-9.80%]	7.61% [6.01%-9.59%]

© 2026 Ne’eman A. *JAMA Health Forum*.

**Sensory or speech disorders: Deafness or hearing difficulty**	8.34% [7.55%-9.21%]	2.43% [1.84%-3.21%]	2.73% [1.68%-4.40%]	3.16% [2.47%-4.03%]	2.87% [2.05%-4.00%]	24.32% [22.18%-26.60%]	3.72% [2.67%-5.16%]
**Cardiovascular system disorders: Any other or unspecified**	7.76% [7.03%-8.56%]	5.64% [4.78%-6.64%]	11.52% [9.26%-14.24%]	13.11% [11.66%-14.70%]	11.11% [9.34%-13.16%]	9.80% [8.48%-11.31%]	9.11% [7.42%-11.14%]
**Musculoskeletal issues: Arthritis (other or unspecified)**	7.13% [6.44%-7.89%]	4.42% [3.71%-5.27%]	10.49% [8.47%-12.91%]	13.30% [11.86%-14.88%]	8.10% [6.66%-9.81%]	9.00% [7.79%-10.37%]	6.22% [4.86%-7.92%]
**Neurodevelopmental or neurobehavioral disorders: Autism Spectrum Disorder and Asperger Syndrome**	6.09% [5.30%-6.99%]	11.24% [9.75%-12.93%]	8.27% [6.14%-11.06%]	2.11% [1.44%-3.08%]	9.38% [7.51%-11.66%]	1.19% [0.73%-1.95%]	2.72% [1.71%-4.28%]
**Sensory or speech disorders: Blindness or vision problems**	5.32% [4.63%-6.11%]	2.92% [2.19%-3.89%]	4.21% [2.90%-6.09%]	3.56% [2.85%-4.44%]	5.38% [4.21%-6.85%]	3.93% [3.14%-4.90%]	22.72% [19.71%-26.03%]
**Endocrine disorders: Diabetes**	5.15% [4.56%-5.83%]	4.28% [3.52%-5.18%]	7.76% [5.93%-10.10%]	8.25% [7.11%-9.56%]	6.86% [5.51%-8.50%]	4.91% [3.99%-6.01%]	7.38% [5.80%-9.36%]
**Other: Any other conditions, including those not sufficiently specific to classify**	4.98% [4.35%-5.69%]	5.39% [4.46%-6.51%]	6.51% [4.78%-8.81%]	6.55% [5.48%-7.81%]	5.75% [4.47%-7.37%]	3.59% [2.76%-4.67%]	4.91% [3.59%-6.68%]
**Cardiovascular system disorders: High blood pressure**	3.93% [3.42%-4.51%]	2.85% [2.27%-3.59%]	3.62% [2.61%-5.01%]	5.03% [4.18%-6.03%]	3.69% [2.84%-4.77%]	4.52% [3.64%-5.59%]	5.28% [4.05%-6.85%]

© 2026 Ne'eman A. *JAMA Health Forum*.

**Mental or emotional disorders: Any other or unspecified**	3.68% [3.14%-4.32%]	6.58% [5.54%-7.80%]	3.72% [2.50%-5.48%]	2.86% [2.18%-3.74%]	6.03% [4.75%-7.64%]	1.79% [1.21%-2.62%]	3.00% [2.09%-4.28%]
**Mental or emotional disorders: Trauma and stressor-related disorders**	3.10% [2.58%-3.72%]	4.53% [3.63%-5.64%]	4.29% [2.63%-6.92%]	3.37% [2.61%-4.35%]	3.39% [2.48%-4.62%]	3.59% [2.74%-4.70%]	2.20% [1.40%-3.43%]
**Neurodevelopmental or neurobehavioral disorders: Learning disability**	2.45% [1.96%-3.05%]	4.25% [3.34%-5.41%]	1.09% [0.49%-2.39%]	1.08% [0.67%-1.72%]	1.58% [0.94%-2.66%]	0.67% [0.38%-1.19%]	0.79% [0.40%-1.55%]
**Neurological disorders: Stroke or Brain Aneurysm**	2.42% [2.03%-2.87%]	3.02% [2.41%-3.79%]	5.47% [4.06%-7.34%]	4.62% [3.79%-5.62%]	5.19% [4.09%-6.56%]	2.67% [2.01%-3.53%]	2.30% [1.54%-3.43%]
**Respiratory disorders: Chronic Obstructive Pulmonary Disease (COPD)**	2.37% [1.94%-2.89%]	1.54% [1.12%-2.12%]	3.71% [2.55%-5.39%]	4.31% [3.53%-5.24%]	4.17% [3.17%-5.47%]	2.82% [2.16%-3.66%]	2.64% [1.47%-4.70%]
**Mental or emotional disorders: Bipolar disorder**	2.36% [1.93%-2.89%]	4.32% [3.48%-5.36%]	3.08% [1.91%-4.93%]	1.61% [1.11%-2.33%]	3.18% [2.28%-4.43%]	1.65% [1.10%-2.49%]	1.08% [0.57%-2.04%]
**Cancer, tumor, cyst, or growth**	2.04% [1.68%-2.46%]	1.76% [1.31%-2.37%]	3.09% [2.10%-4.53%]	3.00% [2.40%-3.75%]	3.05% [2.27%-4.09%]	2.24% [1.70%-2.95%]	2.13% [1.38%-3.26%]
**Neurodevelopmental or neurobehavioral disorders: Any other and unspecified**	1.86% [1.48%-2.34%]	3.18% [2.48%-4.06%]	4.33% [2.94%-6.33%]	1.44% [0.96%-2.16%]	3.41% [2.40%-4.83%]	0.71% [0.33%-1.54%]	2.33% [1.41%-3.83%]

© 2026 Ne’eman A. *JAMA Health Forum*.

**Neurodevelopmental or neurobehavioral disorders: Intellectual disability (due to a congenital disorder, such as Down Syndrome, or some other cause)**	1.76% [1.37%-2.26%]	3.08% [2.34%-4.03%]	3.98% [2.43%-6.44%]	1.07% [0.68%-1.68%]	4.12% [2.93%-5.76%]	0.46% [0.22%-0.93%]	0.95% [0.50%-1.82%]
**Genitourinary disorders**	1.66% [1.32%-2.07%]	1.34% [0.94%-1.90%]	3.27% [2.16%-4.91%]	2.45% [1.85%-3.24%]	2.43% [1.66%-3.54%]	1.63% [1.14%-2.32%]	2.00% [1.30%-3.08%]
**Respiratory disorders: Any other or unspecified**	1.61% [1.30%-2.00%]	1.63% [1.16%-2.27%]	2.78% [1.84%-4.19%]	2.54% [1.96%-3.27%]	2.45% [1.74%-3.43%]	1.66% [1.15%-2.38%]	1.62% [0.99%-2.64%]
**Sensory or speech disorders: Any other or unspecified (including speech disorders, vestibular problems)**	1.54% [1.19%-1.99%]	1.43% [0.90%-2.25%]	1.73% [1.04%-2.87%]	1.92% [1.42%-2.58%]	1.93% [1.21%-3.06%]	1.64% [1.17%-2.29%]	1.46% [0.88%-2.39%]
**Respiratory disorders: Asthma**	1.43% [1.03%-1.98%]	1.54% [1.01%-2.35%]	1.51% [0.67%-3.41%]	0.89% [0.53%-1.47%]	0.91% [0.46%-1.77%]	0.85% [0.45%-1.58%]	1.24% [0.41%-3.73%]
**Digestive system disorders (including liver conditions, stomach problems)**	1.35% [0.99%-1.84%]	1.61% [1.10%-2.34%]	1.42% [0.84%-2.41%]	1.56% [1.12%-2.16%]	1.75% [1.07%-2.83%]	1.01% [0.63%-1.61%]	1.77% [0.80%-3.89%]

© 2026 Ne'eman A. *JAMA Health Forum*.

**Neurological disorders: Epilepsy or seizures**	1.22% [0.92%-1.63%]	2.13% [1.55%-2.91%]	2.68% [1.58%-4.51%]	1.36% [0.91%-2.03%]	3.29% [2.29%-4.71%]	0.74% [0.44%-1.25%]	1.37% [0.77%-2.44%]
**Other: Aging**	1.13% [0.90%-1.40%]	0.77% [0.54%-1.10%]	1.44% [0.93%-2.23%]	1.72% [1.30%-2.28%]	1.41% [0.97%-2.03%]	1.96% [1.45%-2.65%]	1.54% [0.98%-2.42%]
**Neurological disorders: Dementia or Alzheimer's disease**	1.10% [0.88%-1.38%]	2.15% [1.71%-2.70%]	3.11% [2.23%-4.33%]	1.77% [1.33%-2.36%]	3.46% [2.69%-4.44%]	1.69% [1.23%-2.32%]	1.42% [0.90%-2.23%]
**Other: Pain (unspecified)**	1.10% [0.84%-1.43%]	1.14% [0.79%-1.64%]	1.99% [1.18%-3.33%]	1.64% [1.17%-2.29%]	1.84% [1.23%-2.76%]	1.29% [0.85%-1.97%]	1.74% [1.07%-2.84%]
**Immune system disorders**	0.97% [0.71%-1.33%]	1.18% [0.77%-1.82%]	0.96% [0.41%-2.23%]	1.17% [0.77%-1.77%]	1.44% [0.80%-2.56%]	0.48% [0.26%-0.86%]	1.42% [0.75%-2.70%]
**Musculoskeletal issues: Rheumatoid arthritis**	0.88% [0.65%-1.18%]	0.57% [0.35%-0.92%]	1.68% [0.99%-2.84%]	1.61% [1.14%-2.25%]	1.14% [0.71%-1.82%]	0.97% [0.59%-1.59%]	1.54% [0.86%-2.74%]
**Endocrine disorders: Any other or unspecified**	0.32% [0.18%-0.56%]	0.30% [0.14%-0.63%]	0.12% [0.02%-0.85%]	0.16% [0.06%-0.42%]	0.21% [0.07%-0.65%]	0.48% [0.19%-1.17%]	0.28% [0.10%-0.78%]
**Other: COVID-19 or coronavirus**	0.17% [0.08%-0.37%]	0.29% [0.12%-0.71%]	0.87% [0.35%-2.17%]	0.39% [0.17%-0.89%]	0.54% [0.21%-1.34%]	0.07% [0.02%-0.25%]	0.25% [0.05%-1.32%]

All cells column percentages

© 2026 Ne'eman A. *JAMA Health Forum*.

Appendix Table S22: Prevalence of Diagnoses, Overall and by ACS-6 Question - Female Only

Diagnosis	Overall	Cognitive	Self-Care	Mobility	Independent Living	Hearing	Vision
**Musculoskeletal issues: Any other or unspecified**	15.29% [14.32%-16.32%]	10.03% [8.86%-11.34%]	19.28% [16.66%-22.19%]	23.92% [22.37%-25.55%]	16.68% [14.93%-18.59%]	14.69% [12.82%-16.79%]	13.25% [11.35%-15.40%]
**Mental or emotional disorders: Anxiety or obsessive-compulsive disorders**	14.50% [13.45%-15.62%]	23.78% [21.91%-25.76%]	9.93% [8.11%-12.10%]	7.91% [6.92%-9.03%]	15.18% [13.38%-17.17%]	7.80% [6.33%-9.59%]	10.69% [8.83%-12.89%]
**Musculoskeletal issues: Arthritis (other or unspecified)**	13.64% [12.76%-14.56%]	9.21% [8.14%-10.40%]	14.75% [12.76%-16.99%]	20.81% [19.42%-22.27%]	14.60% [13.07%-16.28%]	13.77% [12.11%-15.61%]	12.68% [10.97%-14.61%]
**Mental or emotional disorders: Depression**	12.49% [11.54%-13.51%]	20.99% [19.26%-22.84%]	10.46% [8.62%-12.64%]	9.01% [7.98%-10.17%]	13.12% [11.42%-15.02%]	8.18% [6.70%-9.94%]	9.54% [7.91%-11.46%]
**Musculoskeletal issues: Back or spine problems**	10.71% [9.90%-11.57%]	8.11% [7.07%-9.27%]	14.12% [11.95%-16.60%]	15.63% [14.37%-16.97%]	12.86% [11.35%-14.54%]	10.36% [8.76%-12.20%]	8.15% [6.81%-9.72%]
**Neurological disorders: Any other or unspecified**	9.97% [9.14%-10.87%]	11.39% [10.09%-12.84%]	16.76% [14.14%-19.75%]	12.63% [11.36%-14.01%]	13.48% [11.77%-15.39%]	7.87% [6.48%-9.53%]	10.00% [8.17%-12.18%]
**Neurodevelopmental or neurobehavioral disorders: Attention Deficit Disorder (ADD) or Attention Deficit-Hyperactivity**	8.54% [7.70%-9.46%]	16.55% [14.92%-18.33%]	4.00% [2.88%-5.53%]	2.58% [2.02%-3.30%]	3.97% [3.07%-5.11%]	2.53% [1.75%-3.65%]	2.60% [1.80%-3.73%]

© 2026 Ne’eman A. *JAMA Health Forum*.

**Disorder (ADHD)**							
**Cardiovascular system disorders: Any other or unspecified**	6.23% [5.62%-6.90%]	5.45% [4.63%-6.40%]	8.70% [7.15%-10.55%]	8.92% [7.94%-10.01%]	8.95% [7.70%-10.38%]	7.28% [6.10%-8.66%]	6.50% [5.37%-7.85%]
**Other: Any other conditions, including those not sufficiently specific to classify**	6.00% [5.35%-6.71%]	7.23% [6.17%-8.44%]	7.70% [6.18%-9.56%]	7.23% [6.32%-8.25%]	7.73% [6.54%-9.12%]	4.65% [3.67%-5.88%]	4.40% [3.45%-5.59%]
**Endocrine disorders: Diabetes**	5.78% [5.19%-6.43%]	4.94% [4.16%-5.87%]	8.47% [6.87%-10.41%]	8.30% [7.36%-9.34%]	8.56% [7.32%-10.00%]	5.17% [4.11%-6.48%]	7.55% [6.18%-9.19%]
**Sensory or speech disorders: Deafness or hearing difficulty**	5.18% [4.60%-5.82%]	2.31% [1.76%-3.01%]	3.17% [2.11%-4.73%]	2.26% [1.82%-2.82%]	2.84% [2.19%-3.66%]	21.12% [18.89%-23.54%]	3.13% [2.30%-4.25%]
**Sensory or speech disorders: Blindness or vision problems**	4.89% [4.34%-5.50%]	2.86% [2.27%-3.61%]	4.98% [3.75%-6.59%]	3.95% [3.31%-4.70%]	5.80% [4.80%-6.99%]	3.92% [3.08%-4.97%]	19.16% [16.95%-21.59%]
**Cardiovascular system disorders: High blood pressure**	4.80% [4.26%-5.40%]	3.94% [3.30%-4.71%]	6.87% [5.42%-8.67%]	6.52% [5.67%-7.49%]	6.16% [5.13%-7.37%]	4.03% [3.17%-5.10%]	5.82% [4.67%-7.23%]
**Other: Pain (unspecified)**	4.32% [3.76%-4.96%]	5.26% [4.40%-6.27%]	7.18% [5.50%-9.32%]	6.03% [5.13%-7.07%]	5.92% [4.80%-7.28%]	3.13% [2.25%-4.32%]	4.03% [3.01%-5.37%]
**Mental or emotional disorders: Bipolar disorder**	4.00% [3.42%-4.67%]	7.32% [6.19%-8.62%]	4.64% [3.10%-6.88%]	3.02% [2.33%-3.90%]	5.08% [3.92%-6.55%]	2.82% [1.97%-4.02%]	3.87% [2.57%-5.79%]

© 2026 Ne’eman A. *JAMA Health Forum*.

**Mental or emotional disorders: Any other or unspecified**	3.25% [2.72%-3.88%]	6.00% [4.98%-7.22%]	3.26% [2.06%-5.13%]	2.00% [1.46%-2.74%]	3.94% [2.97%-5.20%]	2.38% [1.54%-3.66%]	3.07% [1.98%-4.74%]
**Mental or emotional disorders: Trauma and stressor-related disorders**	2.97% [2.47%-3.56%]	5.31% [4.36%-6.47%]	3.26% [2.23%-4.74%]	2.13% [1.61%-2.81%]	2.95% [2.20%-3.94%]	1.44% [0.85%-2.43%]	2.03% [1.21%-3.39%]
**Immune system disorders**	2.39% [1.97%-2.91%]	2.77% [2.10%-3.66%]	3.92% [2.70%-5.65%]	2.85% [2.24%-3.62%]	3.07% [2.25%-4.19%]	1.92% [1.26%-2.93%]	2.48% [1.61%-3.82%]
**Respiratory disorders: Chronic Obstructive Pulmonary Disease (COPD)**	2.31% [1.95%-2.74%]	2.05% [1.55%-2.70%]	3.78% [2.80%-5.08%]	3.51% [2.92%-4.23%]	3.67% [2.90%-4.63%]	2.84% [2.13%-3.78%]	2.36% [1.71%-3.24%]
**Musculoskeletal issues: Rheumatoid arthritis**	2.25% [1.86%-2.72%]	1.71% [1.20%-2.44%]	4.04% [2.92%-5.58%]	3.44% [2.83%-4.18%]	2.93% [2.25%-3.81%]	1.37% [0.90%-2.11%]	2.00% [1.35%-2.96%]
**Cancer, tumor, cyst, or growth**	1.98% [1.65%-2.39%]	1.76% [1.32%-2.34%]	3.27% [2.25%-4.74%]	2.56% [2.04%-3.20%]	3.53% [2.72%-4.58%]	2.39% [1.70%-3.36%]	2.24% [1.48%-3.37%]
**Respiratory disorders: Asthma**	1.96% [1.61%-2.39%]	1.66% [1.20%-2.28%]	2.70% [1.80%-4.03%]	2.43% [1.92%-3.06%]	2.16% [1.57%-2.96%]	1.55% [0.98%-2.47%]	2.22% [1.43%-3.43%]
**Neurological disorders: Stroke or Brain Aneurysm**	1.93% [1.59%-2.34%]	2.46% [1.89%-3.19%]	3.82% [2.78%-5.22%]	2.51% [2.01%-3.13%]	3.10% [2.41%-3.97%]	2.27% [1.60%-3.20%]	2.55% [1.81%-3.58%]
**Digestive system disorders (including liver conditions, stomach problems)**	1.92% [1.57%-2.34%]	2.30% [1.77%-2.99%]	2.37% [1.55%-3.60%]	2.37% [1.86%-3.02%]	2.10% [1.53%-2.86%]	1.97% [1.34%-2.89%]	1.96% [1.30%-2.96%]

© 2026 Ne'eman A. *JAMA Health Forum*.

**Neurodevelopmental or neurobehavioral disorders: Autism Spectrum Disorder and Asperger Syndrome**	1.87% [1.50%-2.33%]	3.56% [2.83%-4.47%]	3.26% [2.31%-4.59%]	0.63% [0.39%-1.01%]	1.63% [1.08%-2.47%]	1.33% [0.71%-2.47%]	1.16% [0.59%-2.26%]
**Other: Aging**	1.83% [1.52%-2.21%]	1.43% [1.06%-1.93%]	3.01% [2.11%-4.27%]	2.39% [1.90%-3.01%]	2.93% [2.30%-3.72%]	3.65% [2.80%-4.74%]	1.72% [1.19%-2.49%]
**Sensory or speech disorders: Any other or unspecified (including speech disorders, vestibular problems)**	1.72% [1.41%-2.09%]	1.40% [1.04%-1.88%]	2.41% [1.65%-3.52%]	2.38% [1.90%-2.98%]	2.65% [2.02%-3.47%]	2.63% [1.90%-3.61%]	1.59% [1.03%-2.45%]
**Neurological disorders: Dementia or Alzheimer's disease**	1.62% [1.34%-1.95%]	3.26% [2.69%-3.93%]	5.71% [4.52%-7.19%]	2.30% [1.86%-2.84%]	4.54% [3.75%-5.50%]	2.29% [1.67%-3.13%]	1.93% [1.38%-2.69%]
**Neurodevelopmental or neurobehavioral disorders: Learning disability**	1.52% [1.18%-1.97%]	2.42% [1.78%-3.26%]	0.92% [0.37%-2.28%]	0.86% [0.54%-1.34%]	1.18% [0.76%-1.81%]	1.02% [0.58%-1.80%]	1.45% [0.77%-2.73%]
**Respiratory disorders: Any other or unspecified**	1.36% [1.08%-1.70%]	0.80% [0.53%-1.19%]	2.28% [1.45%-3.57%]	2.21% [1.72%-2.82%]	2.08% [1.48%-2.91%]	1.02% [0.61%-1.72%]	0.81% [0.49%-1.34%]
**Neurodevelopmental or neurobehavioral disorders:**	1.33% [1.01%-1.75%]	2.49% [1.86%-3.34%]	4.66% [3.18%-6.77%]	1.41% [0.94%-2.11%]	3.14% [2.25%-4.37%]	0.72% [0.39%-1.32%]	1.10% [0.54%-2.24%]

© 2026 Ne'eman A. *JAMA Health Forum*.

**Intellectual disability (due to a congenital disorder, such as Down Syndrome, or some other cause)**							
**Neurodevelopmental or neurobehavioral disorders: Any other and unspecified**	1.31% [0.99%-1.75%]	2.29% [1.66%-3.15%]	3.44% [2.18%-5.37%]	1.28% [0.85%-1.94%]	1.76% [1.09%-2.82%]	0.50% [0.24%-1.08%]	1.02% [0.50%-2.08%]
**Endocrine disorders: Any other or unspecified**	1.31% [1.02%-1.67%]	0.95% [0.60%-1.49%]	1.93% [1.19%-3.12%]	1.81% [1.36%-2.39%]	1.26% [0.80%-1.99%]	1.21% [0.76%-1.93%]	0.93% [0.52%-1.64%]
**Genitourinary disorders**	1.13% [0.88%-1.45%]	1.04% [0.69%-1.57%]	2.08% [1.37%-3.17%]	1.50% [1.13%-1.98%]	2.01% [1.39%-2.90%]	0.93% [0.56%-1.54%]	2.10% [1.40%-3.15%]
**Neurological disorders: Epilepsy or seizures**	0.99% [0.75%-1.31%]	1.47% [1.04%-2.06%]	1.44% [0.86%-2.40%]	0.74% [0.48%-1.15%]	1.82% [1.24%-2.66%]	0.61% [0.28%-1.31%]	0.72% [0.36%-1.45%]
**Other: COVID-19 or coronavirus**	0.50% [0.32%-0.80%]	0.65% [0.37%-1.12%]	0.81% [0.35%-1.85%]	0.64% [0.34%-1.19%]	0.75% [0.37%-1.52%]	0.30% [0.11%-0.83%]	0.19% [0.04%-0.83%]

All cells column percentages

© 2026 Ne’eman A. *JAMA Health Forum*.

Appendix Table S23: Prevalence of Diagnoses, Overall and by ACS-6 Question - No HS Diploma Only

Diagnosis	Overall	Cognitive	Self-Care	Mobility	Independent Living	Hearing	Vision
**Musculoskeletal issues: Arthritis (other or unspecified)**	16.39% [14.51%-18.47%]	13.85% [11.41%-16.71%]	18.76% [14.75%-23.57%]	22.08% [19.42%-25.00%]	17.79% [14.79%-21.23%]	16.79% [13.53%-20.64%]	14.21% [11.09%-18.04%]
**Musculoskeletal issues: Any other or unspecified**	15.80% [13.86%-17.96%]	13.07% [10.45%-16.23%]	18.54% [14.14%-23.93%]	21.29% [18.56%-24.31%]	16.78% [13.66%-20.45%]	14.10% [10.98%-17.93%]	14.12% [10.57%-18.62%]
**Musculoskeletal issues: Back or spine problems**	13.28% [11.48%-15.31%]	10.94% [8.72%-13.65%]	15.50% [11.82%-20.06%]	16.46% [14.08%-19.16%]	11.34% [8.90%-14.36%]	12.27% [9.08%-16.38%]	8.51% [6.13%-11.71%]
**Cardiovascular system disorders: Any other or unspecified**	10.98% [9.47%-12.69%]	11.35% [9.19%-13.93%]	10.91% [8.12%-14.51%]	13.01% [11.00%-15.33%]	12.54% [10.03%-15.57%]	13.76% [10.69%-17.54%]	10.32% [7.86%-13.44%]
**Endocrine disorders: Diabetes**	10.42% [8.93%-12.11%]	9.03% [7.09%-11.44%]	11.34% [8.35%-15.22%]	12.01% [10.05%-14.29%]	10.89% [8.63%-13.66%]	9.50% [7.11%-12.58%]	10.48% [7.74%-14.04%]
**Cardiovascular system disorders: High blood pressure**	9.81% [8.37%-11.46%]	9.16% [7.28%-11.47%]	9.11% [6.47%-12.68%]	10.55% [8.79%-12.62%]	9.92% [7.81%-12.53%]	10.13% [7.51%-13.53%]	10.41% [7.81%-13.75%]
**Neurological disorders: Any other or unspecified**	9.53% [7.89%-11.47%]	13.24% [10.36%-16.77%]	13.16% [9.12%-18.61%]	10.24% [8.16%-12.78%]	11.58% [8.69%-15.26%]	8.35% [5.81%-11.85%]	8.21% [5.25%-12.59%]
**Mental or emotional disorders: Depression**	8.45% [6.93%-10.26%]	14.55% [11.71%-17.93%]	8.38% [5.45%-12.68%]	8.69% [6.85%-10.98%]	8.42% [6.19%-11.36%]	7.12% [4.72%-10.62%]	8.78% [6.11%-12.46%]

© 2026 Ne’eman A. *JAMA Health Forum*.

**Mental or emotional disorders: Anxiety or obsessive-compulsive disorders**	7.35% [5.93%-9.07%]	11.40% [8.85%-14.57%]	5.16% [3.13%-8.41%]	5.64% [4.17%-7.58%]	7.55% [5.62%-10.07%]	4.79% [2.76%-8.19%]	5.95% [3.89%-8.98%]
**Sensory or speech disorders: Blindness or vision problems**	5.95% [4.63%-7.63%]	4.29% [2.90%-6.31%]	5.31% [3.36%-8.29%]	4.25% [3.06%-5.87%]	6.23% [4.51%-8.54%]	3.74% [2.42%-5.74%]	17.17% [13.08%-22.22%]
**Sensory or speech disorders: Deafness or hearing difficulty**	5.41% [4.29%-6.82%]	4.39% [2.86%-6.68%]	4.05% [2.38%-6.82%]	3.21% [2.28%-4.50%]	3.87% [2.59%-5.75%]	16.77% [13.21%-21.06%]	5.36% [3.53%-8.05%]
**Other: Any other conditions, including those not sufficiently specific to classify**	4.86% [3.75%-6.27%]	4.74% [3.26%-6.85%]	6.03% [3.81%-9.42%]	4.68% [3.47%-6.28%]	5.79% [4.04%-8.24%]	5.33% [3.38%-8.32%]	4.72% [2.72%-8.08%]
**Respiratory disorders: Chronic Obstructive Pulmonary Disease (COPD)**	4.32% [3.30%-5.63%]	3.87% [2.57%-5.78%]	5.25% [3.23%-8.43%]	5.62% [4.21%-7.48%]	5.94% [4.15%-8.41%]	4.75% [3.00%-7.45%]	3.17% [1.71%-5.79%]
**Mental or emotional disorders: Any other or unspecified**	4.08% [2.93%-5.67%]	7.14% [4.90%-10.29%]	4.66% [2.07%-10.15%]	3.60% [2.24%-5.76%]	4.84% [2.91%-7.93%]	3.83% [2.27%-6.41%]	6.24% [3.44%-11.03%]
**Mental or emotional disorders: Bipolar disorder**	3.34% [2.22%-5.01%]	6.87% [4.50%-10.37%]	5.41% [2.30%-12.23%]	3.20% [1.74%-5.79%]	4.47% [2.37%-8.29%]	2.36% [1.32%-4.19%]	5.53% [2.67%-11.11%]
**Respiratory disorders: Any other or unspecified**	3.25% [2.39%-4.40%]	3.45% [2.18%-5.41%]	5.02% [3.08%-8.08%]	3.48% [2.44%-4.94%]	3.66% [2.42%-5.50%]	2.46% [1.34%-4.47%]	1.63% [0.81%-3.26%]

© 2026 Ne'eman A. *JAMA Health Forum*.

**Other: Pain (unspecified)**	3.15% [2.32%-4.26%]	2.78% [1.77%-4.34%]	2.43% [1.38%-4.26%]	3.15% [2.20%-4.49%]	2.51% [1.57%-4.00%]	2.34% [1.30%-4.17%]	4.23% [2.49%-7.11%]
**Neurological disorders: Stroke or Brain Aneurysm**	3.04% [2.23%-4.13%]	3.42% [2.17%-5.35%]	4.96% [3.20%-7.63%]	4.01% [2.86%-5.58%]	5.08% [3.49%-7.33%]	4.17% [2.39%-7.20%]	2.46% [1.42%-4.24%]
**Neurological disorders: Dementia or Alzheimer's disease**	2.43% [1.77%-3.32%]	5.20% [3.78%-7.11%]	6.29% [4.27%-9.18%]	3.00% [2.09%-4.29%]	5.20% [3.81%-7.07%]	2.88% [1.74%-4.72%]	1.61% [0.81%-3.17%]
**Neurodevelopmental or neurobehavioral disorders: Attention Deficit Disorder (ADD) or Attention Deficit-Hyperactivity Disorder (ADHD)**	2.39% [1.62%-3.51%]	5.00% [3.37%-7.35%]	1.41% [0.42%-4.59%]	1.29% [0.57%-2.87%]	1.99% [1.00%-3.91%]	1.17% [0.37%-3.66%]	1.69% [0.58%-4.79%]
**Other: Aging**	2.32% [1.66%-3.23%]	1.94% [1.17%-3.21%]	4.39% [2.63%-7.24%]	2.32% [1.53%-3.51%]	3.64% [2.42%-5.45%]	4.11% [2.58%-6.49%]	2.86% [1.67%-4.87%]
**Cancer, tumor, cyst, or growth**	2.20% [1.53%-3.16%]	1.89% [1.20%-2.96%]	2.08% [1.12%-3.84%]	2.37% [1.60%-3.49%]	2.75% [1.73%-4.34%]	1.79% [0.99%-3.21%]	1.68% [0.61%-4.56%]
**Genitourinary disorders**	2.17% [1.48%-3.18%]	1.71% [1.02%-2.87%]	3.37% [1.75%-6.38%]	2.37% [1.48%-3.76%]	2.68% [1.55%-4.59%]	2.19% [1.12%-4.25%]	3.64% [2.04%-6.41%]
**Sensory or speech disorders: Any other or unspecified (including speech disorders, vestibular problems)**	1.95% [1.30%-2.92%]	2.15% [1.22%-3.78%]	3.61% [1.96%-6.53%]	2.78% [1.80%-4.26%]	3.35% [2.05%-5.43%]	2.46% [1.40%-4.29%]	3.47% [1.94%-6.14%]

© 2026 Ne'eman A. *JAMA Health Forum*.

**Neurodevelopmental or neurobehavioral disorders: Intellectual disability (due to a congenital disorder, such as Down Syndrome, or some other cause)**	1.66% [0.98%-2.79%]	3.09% [1.75%-5.41%]	2.96% [1.16%-7.35%]	1.16% [0.49%-2.73%]	2.82% [1.50%-5.25%]	0.30% [0.09%-1.04%]	2.22% [0.79%-6.05%]
**Mental or emotional disorders: Trauma and stressor-related disorders**	1.66% [1.03%-2.66%]	3.05% [1.78%-5.17%]	0.88% [0.27%-2.84%]	1.53% [0.84%-2.77%]	1.04% [0.45%-2.41%]	1.15% [0.51%-2.59%]	0.74% [0.26%-2.10%]
**Musculoskeletal issues: Rheumatoid arthritis**	1.65% [1.12%-2.44%]	1.25% [0.68%-2.28%]	2.47% [1.32%-4.55%]	2.26% [1.47%-3.47%]	1.91% [1.12%-3.25%]	1.11% [0.48%-2.54%]	1.34% [0.54%-3.28%]
**Respiratory disorders: Asthma**	1.64% [1.06%-2.53%]	1.21% [0.64%-2.27%]	2.78% [1.52%-5.05%]	2.04% [1.28%-3.22%]	1.99% [1.15%-3.41%]	1.67% [0.67%-4.10%]	0.54% [0.18%-1.66%]
**Digestive system disorders (including liver conditions, stomach problems)**	1.59% [1.06%-2.36%]	1.85% [1.07%-3.20%]	2.37% [1.25%-4.45%]	2.08% [1.34%-3.23%]	1.65% [0.94%-2.88%]	1.72% [0.87%-3.40%]	1.57% [0.67%-3.64%]
**Neurodevelopmental or neurobehavioral disorders: Learning disability**	1.37% [0.80%-2.31%]	1.87% [0.91%-3.78%]	0.30% [0.07%-1.22%]	1.12% [0.56%-2.25%]	1.18% [0.52%-2.64%]	1.18% [0.44%-3.13%]	0.75% [0.11%-5.14%]
**Neurodevelopmental or neurobehavioral disorders: Autism**	1.34% [0.77%-2.32%]	2.93% [1.69%-5.04%]	1.68% [0.62%-4.43%]	0.57% [0.21%-1.52%]	3.08% [1.73%-5.42%]	0.49% [0.07%-3.43%]	0.74% [0.17%-3.18%]

© 2026 Ne’eman A. *JAMA Health Forum*.

**Spectrum Disorder and Asperger Syndrome**							
**Neurological disorders: Epilepsy or seizures**	1.03% [0.55%-1.91%]	2.11% [1.10%-4.03%]	2.09% [0.83%-5.15%]	0.95% [0.44%-2.04%]	2.12% [1.05%-4.25%]	0.53% [0.16%-1.73%]	0.88% [0.31%-2.47%]
**Neurodevelopmental or neurobehavioral disorders: Any other and unspecified**	0.93% [0.45%-1.90%]	1.81% [0.81%-3.96%]	2.92% [1.17%-7.08%]	0.94% [0.34%-2.56%]	1.98% [0.88%-4.35%]	0.20% [0.05%-0.82%]	1.26% [0.27%-5.78%]
**Endocrine disorders: Any other or unspecified**	0.91% [0.47%-1.73%]	0.66% [0.23%-1.83%]	0.30% [0.07%-1.24%]	1.03% [0.48%-2.23%]	0.66% [0.17%-2.60%]	0.55% [0.17%-1.76%]	0.62% [0.17%-2.18%]
**Immune system disorders**	0.89% [0.47%-1.69%]	1.05% [0.42%-2.59%]	1.42% [0.50%-3.96%]	0.86% [0.41%-1.83%]	1.17% [0.50%-2.69%]	0.57% [0.14%-2.38%]	0.34% [0.08%-1.42%]
**Other: COVID-19 or coronavirus**	0.22% [0.06%-0.74%]	0.39% [0.09%-1.61%]	0.00% [0.00%-0.00%]	0.17% [0.04%-0.69%]	0.00% [0.00%-0.00%]	0.00% [0.00%-0.00%]	0.00% [0.00%-0.00%]

All cells column percentages

© 2026 Ne’eman A. *JAMA Health Forum*.

Appendix Table S24: Prevalence of Diagnoses, Overall and by ACS-6 Question - HS Diploma

Diagnosis	Overall	Cognitive	Self-Care	Mobility	Independent Living	Hearing	Vision
**Musculoskeletal issues: Any other or unspecified**	16.05% [14.71%-17.48%]	11.19% [9.51%-13.13%]	17.44% [14.19%-21.25%]	24.33% [22.22%-26.57%]	15.07% [12.81%-17.65%]	13.23% [11.20%-15.56%]	13.36% [10.81%-16.40%]
**Musculoskeletal issues: Arthritis (other or unspecified)**	11.71% [10.61%-12.91%]	7.69% [6.42%-9.18%]	13.92% [11.27%-17.08%]	17.50% [15.71%-19.45%]	10.13% [8.39%-12.18%]	11.91% [10.11%-13.97%]	9.99% [8.03%-12.37%]
**Musculoskeletal issues: Back or spine problems**	11.52% [10.39%-12.76%]	9.96% [8.34%-11.86%]	14.78% [11.76%-18.41%]	16.23% [14.45%-18.18%]	12.18% [10.17%-14.54%]	10.80% [8.92%-13.02%]	11.00% [8.75%-13.74%]
**Mental or emotional disorders: Depression**	10.91% [9.69%-12.26%]	18.72% [16.43%-21.25%]	9.39% [7.13%-12.28%]	8.21% [6.96%-9.68%]	9.98% [8.18%-12.13%]	6.31% [4.86%-8.17%]	9.31% [6.77%-12.67%]
**Neurological disorders: Any other or unspecified**	10.75% [9.62%-12.00%]	12.61% [10.78%-14.71%]	18.17% [14.72%-22.21%]	14.21% [12.49%-16.12%]	13.73% [11.57%-16.22%]	8.63% [6.87%-10.78%]	10.99% [8.53%-14.06%]
**Mental or emotional disorders: Anxiety or obsessive-compulsive disorders**	10.58% [9.41%-11.87%]	16.48% [14.43%-18.75%]	8.56% [6.46%-11.26%]	7.34% [6.11%-8.79%]	11.88% [9.89%-14.21%]	7.08% [5.46%-9.14%]	9.70% [7.43%-12.59%]
**Cardiovascular system disorders: Any other or unspecified**	8.34% [7.36%-9.44%]	7.57% [6.21%-9.19%]	12.41% [9.72%-15.71%]	11.48% [9.90%-13.27%]	10.54% [8.67%-12.75%]	9.75% [8.06%-11.75%]	9.67% [7.65%-12.15%]
**Sensory or speech disorders: Deafness or hearing difficulty**	6.79% [5.90%-7.81%]	2.63% [1.84%-3.74%]	3.98% [2.36%-6.63%]	2.71% [2.05%-3.57%]	2.95% [2.09%-4.14%]	21.31% [18.65%-24.24%]	3.56% [2.31%-5.44%]

© 2026 Ne’eman A. *JAMA Health Forum*.

**Endocrine disorders: Diabetes**	6.63% [5.77%-7.61%]	6.78% [5.52%-8.31%]	10.70% [8.16%-13.92%]	8.96% [7.61%-10.51%]	9.13% [7.41%-11.20%]	5.10% [3.92%-6.60%]	7.95% [6.05%-10.38%]
**Sensory or speech disorders: Blindness or vision problems**	5.84% [4.96%-6.87%]	3.96% [2.81%-5.57%]	5.78% [3.77%-8.76%]	4.04% [3.17%-5.13%]	6.29% [4.85%-8.13%]	5.24% [4.03%-6.79%]	21.43% [18.06%-25.23%]
**Cardiovascular system disorders: High blood pressure**	5.28% [4.52%-6.17%]	4.97% [3.92%-6.28%]	6.90% [4.88%-9.68%]	6.10% [4.97%-7.47%]	4.79% [3.59%-6.38%]	4.37% [3.24%-5.87%]	5.52% [4.00%-7.59%]
**Other: Any other conditions, including those not sufficiently specific to classify**	4.94% [4.20%-5.80%]	5.89% [4.73%-7.32%]	5.87% [4.17%-8.20%]	6.32% [5.22%-7.62%]	6.64% [5.26%-8.34%]	3.77% [2.85%-4.97%]	4.63% [3.31%-6.44%]
**Neurodevelopmental or neurobehavioral disorders: Attention Deficit Disorder (ADD) or Attention Deficit-Hyperactivity Disorder (ADHD)**	4.93% [4.10%-5.92%]	9.67% [7.98%-11.67%]	2.81% [1.68%-4.66%]	1.66% [1.15%-2.37%]	2.89% [2.05%-4.06%]	1.79% [1.04%-3.05%]	2.96% [1.92%-4.54%]
**Mental or emotional disorders: Any other or unspecified**	4.53% [3.75%-5.47%]	8.76% [7.17%-10.66%]	3.54% [2.17%-5.72%]	2.49% [1.79%-3.45%]	6.08% [4.65%-7.91%]	2.86% [1.78%-4.58%]	3.03% [1.96%-4.64%]
**Mental or emotional disorders: Bipolar disorder**	3.48% [2.82%-4.30%]	6.53% [5.21%-8.15%]	2.25% [1.25%-4.01%]	1.96% [1.37%-2.81%]	3.69% [2.60%-5.20%]	2.40% [1.49%-3.83%]	1.64% [0.89%-3.01%]
**Respiratory disorders: Chronic Obstructive Pulmonary Disease (COPD)**	2.94% [2.42%-3.57%]	2.92% [2.13%-3.98%]	4.54% [3.09%-6.62%]	4.76% [3.86%-5.86%]	3.97% [3.01%-5.24%]	3.42% [2.53%-4.61%]	2.80% [1.90%-4.09%]

© 2026 Ne’eman A. *JAMA Health Forum*.

**Neurodevelopmental or neurobehavioral disorders: Autism Spectrum Disorder and Asperger Syndrome**	2.81% [2.15%-3.67%]	5.15% [3.88%-6.81%]	2.80% [1.58%-4.91%]	0.87% [0.43%-1.72%]	5.35% [3.87%-7.36%]	0.91% [0.38%-2.12%]	1.60% [0.80%-3.16%]
**Mental or emotional disorders: Trauma and stressor-related disorders**	2.80% [2.22%-3.53%]	4.60% [3.53%-5.97%]	3.02% [1.87%-4.84%]	2.42% [1.74%-3.37%]	2.88% [2.01%-4.10%]	3.60% [2.47%-5.22%]	2.95% [1.72%-5.02%]
**Neurodevelopmental or neurobehavioral disorders: Intellectual disability (due to a congenital disorder, such as Down Syndrome, or some other cause)**	2.73% [2.09%-3.56%]	5.21% [3.91%-6.91%]	7.80% [5.13%-11.68%]	2.26% [1.52%-3.35%]	6.67% [4.95%-8.94%]	0.74% [0.35%-1.53%]	1.71% [0.93%-3.13%]
**Other: Pain (unspecified)**	2.63% [2.09%-3.31%]	3.47% [2.57%-4.69%]	4.27% [2.59%-6.96%]	3.60% [2.73%-4.72%]	3.87% [2.73%-5.46%]	1.90% [1.16%-3.11%]	2.51% [1.58%-3.97%]
**Neurological disorders: Stroke or Brain Aneurysm**	2.54% [2.04%-3.16%]	3.50% [2.63%-4.65%]	3.98% [2.69%-5.84%]	3.14% [2.43%-4.04%]	3.37% [2.47%-4.58%]	3.30% [2.39%-4.56%]	3.15% [2.07%-4.76%]
**Cancer, tumor, cyst, or growth**	2.20% [1.73%-2.78%]	2.53% [1.78%-3.59%]	3.10% [2.00%-4.78%]	2.55% [1.93%-3.37%]	2.76% [1.97%-3.86%]	1.85% [1.19%-2.87%]	1.84% [1.09%-3.10%]
**Other: Aging**	1.88% [1.47%-2.42%]	1.41% [0.94%-2.11%]	1.87% [1.12%-3.10%]	2.48% [1.83%-3.34%]	1.89% [1.31%-2.72%]	3.21% [2.29%-4.48%]	2.01% [1.25%-3.21%]
**Digestive system disorders (including liver**	1.84% [1.36%-2.49%]	2.82% [1.93%-4.11%]	1.59% [0.84%-2.99%]	1.94% [1.39%-2.69%]	2.10% [1.28%-3.43%]	1.35% [0.79%-2.28%]	1.35% [0.73%-2.49%]

© 2026 Ne’eman A. *JAMA Health Forum*.

**conditions, stomach problems)**							
**Musculoskeletal issues: Rheumatoid arthritis**	1.84% [1.44%-2.35%]	1.45% [0.97%-2.17%]	4.54% [3.09%-6.61%]	3.00% [2.31%-3.91%]	2.39% [1.66%-3.43%]	1.59% [1.01%-2.48%]	2.43% [1.50%-3.92%]
**Sensory or speech disorders: Any other or unspecified (including speech disorders, vestibular problems)**	1.76% [1.33%-2.31%]	1.56% [0.97%-2.50%]	2.18% [1.31%-3.60%]	2.32% [1.72%-3.13%]	2.89% [1.95%-4.28%]	2.19% [1.51%-3.19%]	1.18% [0.65%-2.15%]
**Neurodevelopmental or neurobehavioral disorders: Learning disability**	1.75% [1.25%-2.43%]	3.00% [2.06%-4.36%]	0.89% [0.19%-4.10%]	0.76% [0.37%-1.55%]	1.89% [1.15%-3.09%]	1.08% [0.58%-2.01%]	0.87% [0.43%-1.77%]
**Neurological disorders: Dementia or Alzheimer's disease**	1.71% [1.34%-2.19%]	3.44% [2.66%-4.43%]	4.50% [3.14%-6.42%]	2.35% [1.76%-3.13%]	4.20% [3.19%-5.50%]	2.37% [1.61%-3.47%]	1.94% [1.24%-3.02%]
**Neurological disorders: Epilepsy or seizures**	1.61% [1.21%-2.15%]	2.54% [1.80%-3.57%]	2.53% [1.39%-4.57%]	1.32% [0.87%-2.00%]	2.75% [1.89%-3.99%]	1.02% [0.55%-1.90%]	1.33% [0.71%-2.49%]
**Neurodevelopmental or neurobehavioral disorders: Any other and unspecified**	1.54% [1.10%-2.14%]	2.52% [1.71%-3.69%]	5.43% [3.48%-8.37%]	2.07% [1.38%-3.09%]	3.45% [2.32%-5.09%]	0.78% [0.32%-1.89%]	1.89% [1.02%-3.49%]

© 2026 Ne'eman A. *JAMA Health Forum*.

**Respiratory disorders: Asthma**	1.45% [1.04%-2.02%]	1.30% [0.79%-2.11%]	2.71% [1.46%-4.96%]	1.65% [1.11%-2.46%]	1.72% [1.02%-2.87%]	1.05% [0.50%-2.21%]	2.29% [1.22%-4.25%]
**Genitourinary disorders**	1.40% [1.03%-1.92%]	1.04% [0.61%-1.74%]	2.36% [1.29%-4.29%]	1.80% [1.23%-2.62%]	1.93% [1.18%-3.14%]	1.06% [0.62%-1.82%]	1.84% [1.08%-3.12%]
**Immune system disorders**	1.39% [0.98%-1.98%]	1.65% [0.99%-2.74%]	1.59% [0.62%-4.03%]	1.63% [1.05%-2.52%]	1.65% [0.90%-3.01%]	0.60% [0.29%-1.23%]	2.22% [1.19%-4.08%]
**Respiratory disorders: Any other or unspecified**	1.29% [0.96%-1.73%]	1.21% [0.74%-1.97%]	1.17% [0.56%-2.44%]	1.80% [1.28%-2.52%]	1.52% [0.96%-2.40%]	1.09% [0.64%-1.87%]	0.77% [0.40%-1.47%]
**Endocrine disorders: Any other or unspecified**	0.70% [0.48%-1.04%]	0.64% [0.35%-1.15%]	1.20% [0.60%-2.36%]	1.07% [0.69%-1.65%]	0.92% [0.54%-1.57%]	0.48% [0.23%-1.01%]	0.22% [0.05%-1.07%]
**Other: COVID-19 or coronavirus**	0.26% [0.13%-0.54%]	0.43% [0.17%-1.06%]	0.81% [0.25%-2.61%]	0.36% [0.14%-0.91%]	0.44% [0.14%-1.39%]	0.34% [0.10%-1.09%]	0.31% [0.04%-2.16%]

All cells column percentages

© 2026 Ne’eman A. *JAMA Health Forum*.

Appendix Table S25: Prevalence of Diagnoses, Overall and by ACS-6 Question - Some College

Diagnosis	Overall	Cognitive	Self-Care	Mobility	Independent Living	Hearing	Vision
**Musculoskeletal issues: Any other or unspecified**	17.19% [15.71%-18.77%]	11.07% [9.31%-13.10%]	22.19% [18.31%-26.62%]	25.29% [23.02%-27.71%]	17.37% [14.85%-20.22%]	15.87% [13.36%-18.75%]	13.60% [10.75%-17.07%]
**Musculoskeletal issues: Back or spine problems**	14.12% [12.75%-15.62%]	12.01% [10.05%-14.29%]	22.98% [18.82%-27.73%]	20.62% [18.49%-22.93%]	17.66% [14.95%-20.75%]	13.25% [10.93%-15.97%]	10.32% [7.97%-13.25%]
**Musculoskeletal issues: Arthritis (other or unspecified)**	12.75% [11.52%-14.09%]	9.86% [8.25%-11.74%]	13.02% [10.30%-16.33%]	17.94% [16.06%-19.99%]	14.19% [11.90%-16.84%]	10.77% [8.87%-13.01%]	12.41% [9.65%-15.83%]
**Mental or emotional disorders: Depression**	12.18% [10.78%-13.72%]	23.46% [20.65%-26.53%]	11.48% [8.67%-15.05%]	9.08% [7.53%-10.91%]	15.61% [12.77%-18.95%]	7.22% [5.43%-9.53%]	10.36% [7.90%-13.46%]
**Mental or emotional disorders: Anxiety or obsessive-compulsive disorders**	11.56% [10.21%-13.07%]	21.63% [18.91%-24.62%]	9.27% [6.81%-12.49%]	7.49% [6.07%-9.20%]	16.65% [13.75%-20.02%]	7.60% [5.79%-9.90%]	9.44% [7.10%-12.44%]
**Neurological disorders: Any other or unspecified**	10.89% [9.70%-12.22%]	14.29% [12.22%-16.64%]	17.94% [14.45%-22.07%]	13.86% [12.08%-15.86%]	15.39% [12.90%-18.25%]	9.43% [7.61%-11.64%]	8.95% [6.69%-11.89%]
**Cardiovascular system disorders: Any other or unspecified**	7.97% [6.97%-9.11%]	5.99% [4.78%-7.48%]	10.01% [7.38%-13.44%]	10.51% [8.97%-12.26%]	9.92% [7.92%-12.36%]	8.37% [6.73%-10.35%]	7.34% [5.37%-9.95%]
**Sensory or speech disorders: Deafness or hearing difficulty**	7.11% [6.05%-8.36%]	2.94% [2.04%-4.21%]	2.12% [1.00%-4.46%]	2.29% [1.62%-3.24%]	2.71% [1.75%-4.19%]	23.20% [19.98%-26.77%]	3.03% [1.89%-4.81%]

© 2026 Ne’eman A. *JAMA Health Forum*.

**Neurodevelopmental or neurobehavioral disorders: Attention Deficit Disorder (ADD) or Attention Deficit-Hyperactivity Disorder (ADHD)**	6.43% [5.43%-7.61%]	13.40% [11.23%-15.91%]	3.46% [2.13%-5.58%]	2.01% [1.41%-2.85%]	4.99% [3.59%-6.91%]	2.69% [1.73%-4.15%]	2.71% [1.55%-4.71%]
**Other: Any other conditions, including those not sufficiently specific to classify**	6.18% [5.25%-7.25%]	6.80% [5.33%-8.63%]	7.54% [5.22%-10.77%]	7.82% [6.44%-9.46%]	7.95% [6.07%-10.35%]	4.41% [3.10%-6.23%]	4.61% [3.10%-6.80%]
**Endocrine disorders: Diabetes**	5.73% [4.90%-6.69%]	4.80% [3.69%-6.23%]	7.14% [5.05%-10.01%]	7.82% [6.53%-9.35%]	6.84% [5.21%-8.92%]	5.50% [4.17%-7.22%]	9.36% [7.06%-12.31%]
**Sensory or speech disorders: Blindness or vision problems**	5.25% [4.41%-6.24%]	2.92% [2.10%-4.04%]	3.80% [2.52%-5.68%]	3.61% [2.81%-4.63%]	5.22% [3.91%-6.93%]	3.43% [2.47%-4.76%]	23.51% [19.69%-27.81%]
**Mental or emotional disorders: Trauma and stressor-related disorders**	4.11% [3.32%-5.07%]	7.12% [5.57%-9.06%]	5.64% [3.60%-8.73%]	3.56% [2.59%-4.88%]	4.96% [3.49%-7.00%]	3.42% [2.25%-5.18%]	2.09% [1.11%-3.89%]
**Cardiovascular system disorders: High blood pressure**	3.95% [3.29%-4.74%]	2.77% [1.99%-3.86%]	2.67% [1.68%-4.21%]	4.60% [3.68%-5.74%]	3.60% [2.59%-4.98%]	4.25% [3.14%-5.74%]	5.87% [4.15%-8.25%]
**Mental or emotional disorders: Bipolar disorder**	3.85% [3.08%-4.80%]	7.55% [5.98%-9.51%]	5.31% [3.40%-8.18%]	3.05% [2.18%-4.25%]	6.10% [4.41%-8.38%]	2.89% [1.78%-4.64%]	3.31% [1.91%-5.67%]
**Mental or emotional disorders: Any other or**	3.67% [2.91%-4.61%]	7.48% [5.82%-9.56%]	3.30% [1.98%-5.45%]	1.97% [1.30%-2.96%]	4.47% [3.10%-6.39%]	1.64% [0.90%-2.95%]	3.50% [1.96%-6.16%]

© 2026 Ne’eman A. *JAMA Health Forum*.

**unspecified**							
**Other: Pain (unspecified)**	3.60% [2.91%-4.43%]	5.56% [4.27%-7.22%]	7.27% [5.00%-10.47%]	5.16% [4.06%-6.54%]	6.04% [4.43%-8.17%]	2.20% [1.44%-3.34%]	4.65% [2.95%-7.27%]
**Respiratory disorders: Chronic Obstructive Pulmonary Disease (COPD)**	2.84% [2.19%-3.67%]	1.67% [1.12%-2.49%]	3.76% [2.46%-5.69%]	3.44% [2.67%-4.43%]	3.41% [2.46%-4.69%]	3.24% [2.30%-4.55%]	3.47% [1.58%-7.47%]
**Cancer, tumor, cyst, or growth**	2.61% [2.06%-3.30%]	2.33% [1.58%-3.43%]	4.47% [2.74%-7.22%]	3.56% [2.68%-4.70%]	4.56% [3.18%-6.49%]	3.45% [2.38%-4.97%]	3.73% [2.26%-6.10%]
**Neurological disorders: Stroke or Brain Aneurysm**	2.51% [1.99%-3.17%]	3.37% [2.46%-4.61%]	5.87% [3.94%-8.66%]	3.61% [2.77%-4.69%]	4.76% [3.46%-6.52%]	2.02% [1.34%-3.04%]	2.36% [1.37%-4.02%]
**Immune system disorders**	2.44% [1.88%-3.15%]	2.99% [2.07%-4.31%]	5.49% [3.50%-8.52%]	3.12% [2.29%-4.25%]	3.91% [2.63%-5.77%]	1.30% [0.72%-2.34%]	3.08% [1.83%-5.15%]
**Respiratory disorders: Any other or unspecified**	1.92% [1.43%-2.58%]	1.33% [0.79%-2.23%]	3.20% [1.78%-5.69%]	2.77% [1.99%-3.85%]	2.93% [1.84%-4.63%]	2.15% [1.27%-3.60%]	1.48% [0.80%-2.73%]
**Respiratory disorders: Asthma**	1.69% [1.12%-2.55%]	0.82% [0.42%-1.61%]	1.98% [0.91%-4.24%]	2.06% [1.40%-3.02%]	1.81% [1.03%-3.18%]	1.28% [0.60%-2.68%]	2.24% [0.70%-6.93%]
**Digestive system disorders (including liver conditions, stomach problems)**	1.66% [1.10%-2.50%]	1.79% [1.13%-2.84%]	2.00% [0.94%-4.20%]	1.58% [0.98%-2.53%]	1.30% [0.65%-2.56%]	1.44% [0.80%-2.60%]	2.75% [1.04%-7.09%]

© 2026 Ne’eman A. *JAMA Health Forum*.

**Neurodevelopmental or neurobehavioral disorders: Autism Spectrum Disorder and Asperger Syndrome**	1.61% [1.06%-2.43%]	3.47% [2.24%-5.35%]	0.88% [0.39%-2.00%]	0.55% [0.30%-1.02%]	2.26% [1.24%-4.09%]	0.65% [0.24%-1.76%]	0.45% [0.16%-1.22%]
**Musculoskeletal issues: Rheumatoid arthritis**	1.55% [1.12%-2.14%]	1.09% [0.59%-1.99%]	2.37% [1.28%-4.34%]	2.33% [1.63%-3.32%]	1.83% [1.10%-3.04%]	1.12% [0.57%-2.20%]	2.30% [1.17%-4.48%]
**Genitourinary disorders**	1.45% [1.09%-1.94%]	1.39% [0.86%-2.22%]	2.77% [1.71%-4.47%]	1.85% [1.32%-2.58%]	1.51% [0.92%-2.48%]	1.74% [1.02%-2.95%]	1.88% [1.01%-3.49%]
**Sensory or speech disorders: Any other or unspecified (including speech disorders, vestibular problems)**	1.43% [1.04%-1.96%]	0.64% [0.34%-1.23%]	1.29% [0.59%-2.76%]	2.07% [1.45%-2.93%]	1.20% [0.69%-2.09%]	1.54% [0.92%-2.57%]	1.39% [0.70%-2.73%]
**Neurological disorders: Dementia or Alzheimer's disease**	1.32% [0.97%-1.78%]	2.99% [2.20%-4.07%]	4.59% [3.15%-6.66%]	1.81% [1.28%-2.56%]	3.93% [2.86%-5.37%]	1.50% [0.91%-2.46%]	1.81% [1.03%-3.18%]
**Endocrine disorders: Any other or unspecified**	1.28% [0.85%-1.94%]	1.18% [0.57%-2.44%]	2.07% [0.95%-4.45%]	1.45% [0.89%-2.35%]	1.02% [0.42%-2.48%]	1.09% [0.46%-2.54%]	0.67% [0.26%-1.73%]
**Other: Aging**	1.14% [0.84%-1.54%]	1.15% [0.72%-1.82%]	2.08% [1.14%-3.77%]	1.50% [1.03%-2.16%]	1.68% [1.07%-2.62%]	1.96% [1.30%-2.95%]	1.10% [0.58%-2.07%]
**Neurodevelopmental or neurobehavioral disorders:**	0.86% [0.56%-1.31%]	1.04% [0.57%-1.90%]	0.40% [0.12%-1.40%]	0.58% [0.30%-1.13%]	0.31% [0.08%-1.13%]	0.38% [0.13%-1.05%]	1.09% [0.46%-2.56%]

© 2026 Ne'eman A. *JAMA Health Forum*.

**Learning disability**							
**Other: COVID-19 or coronavirus**	0.57% [0.29%-1.14%]	0.72% [0.32%-1.57%]	1.78% [0.78%-4.01%]	0.92% [0.43%-1.95%]	1.24% [0.58%-2.64%]	0.24% [0.08%-0.69%]	0.22% [0.05%-0.92%]
**Neurodevelopmental or neurobehavioral disorders: Any other and unspecified**	0.56% [0.30%-1.04%]	0.85% [0.35%-2.01%]	0.90% [0.38%-2.16%]	0.54% [0.28%-1.02%]	1.07% [0.52%-2.18%]	0.00% [0.00%-0.00%]	0.31% [0.06%-1.62%]
**Neurodevelopmental or neurobehavioral disorders: Intellectual disability (due to a congenital disorder, such as Down Syndrome, or some other cause)**	0.42% [0.22%-0.81%]	0.86% [0.43%-1.72%]	1.31% [0.54%-3.11%]	0.40% [0.16%-0.97%]	1.06% [0.49%-2.31%]	0.36% [0.10%-1.23%]	0.00% [0.00%-0.00%]
**Neurological disorders: Epilepsy or seizures**	0.36% [0.18%-0.69%]	0.56% [0.26%-1.21%]	0.59% [0.16%-2.17%]	0.30% [0.12%-0.76%]	1.00% [0.48%-2.07%]	0.22% [0.05%-0.90%]	0.21% [0.05%-0.89%]

All cells column percentages

© 2026 Ne’eman A. *JAMA Health Forum*.

Appendix Table S26: Prevalence of Diagnoses, Overall and by ACS-6 Question - BA+

Diagnosis	Overall	Cognitive	Self-Care	Mobility	Independent Living	Hearing	Vision
**Musculoskeletal issues: Any other or unspecified**	13.76% [12.32%-15.33%]	8.60% [6.77%-10.87%]	18.87% [14.76%-23.82%]	23.00% [20.52%-25.69%]	14.07% [11.35%-17.32%]	11.01% [8.92%-13.51%]	12.45% [9.55%-16.07%]
**Mental or emotional disorders: Anxiety or obsessive-compulsive disorders**	10.37% [8.98%-11.95%]	22.35% [19.18%-25.88%]	9.14% [6.24%-13.18%]	5.80% [4.50%-7.45%]	13.02% [10.19%-16.49%]	4.78% [3.38%-6.71%]	5.37% [3.40%-8.40%]
**Mental or emotional disorders: Depression**	9.75% [8.48%-11.19%]	19.84% [16.95%-23.08%]	9.31% [6.39%-13.37%]	6.22% [4.87%-7.91%]	10.65% [8.15%-13.80%]	5.91% [4.38%-7.92%]	5.90% [4.08%-8.48%]
**Musculoskeletal issues: Arthritis (other or unspecified)**	9.50% [8.36%-10.78%]	6.12% [4.51%-8.26%]	11.43% [8.50%-15.20%]	16.36% [14.32%-18.63%]	10.09% [7.94%-12.75%]	9.14% [7.43%-11.19%]	7.43% [5.53%-9.92%]
**Neurological disorders: Any other or unspecified**	9.43% [8.23%-10.78%]	11.48% [9.46%-13.87%]	17.76% [13.46%-23.07%]	13.76% [11.66%-16.18%]	15.63% [12.53%-19.33%]	7.33% [5.65%-9.46%]	9.04% [6.52%-12.42%]
**Sensory or speech disorders: Deafness or hearing difficulty**	9.39% [8.28%-10.62%]	2.14% [1.36%-3.36%]	2.61% [1.14%-5.84%]	2.72% [1.87%-3.94%]	2.28% [1.35%-3.83%]	27.30% [24.31%-30.51%]	2.17% [1.26%-3.70%]
**Musculoskeletal issues: Back or spine problems**	9.38% [8.24%-10.65%]	6.49% [5.02%-8.35%]	13.05% [9.84%-17.11%]	14.55% [12.56%-16.79%]	9.19% [7.10%-11.81%]	8.44% [6.68%-10.60%]	7.03% [5.14%-9.55%]
**Neurodevelopmental or neurobehavioral disorders: Attention Deficit Disorder (ADD) or Attention Deficit-Hyperactivity**	8.14% [6.90%-9.57%]	18.90% [15.96%-22.25%]	3.82% [2.13%-6.75%]	2.81% [1.89%-4.15%]	3.23% [2.11%-4.91%]	2.44% [1.55%-3.82%]	3.48% [2.06%-5.81%]

© 2026 Ne’eman A. *JAMA Health Forum*.

**Disorder (ADHD)**							
**Other: Any other conditions, including those not sufficiently specific to classify**	6.18% [5.11%-7.47%]	9.48% [7.24%-12.32%]	7.62% [5.05%-11.32%]	7.26% [5.71%-9.18%]	7.25% [5.15%-10.13%]	2.69% [1.82%-3.97%]	4.37% [2.83%-6.70%]
**Cardiovascular system disorders: Any other or unspecified**	5.83% [4.94%-6.88%]	4.69% [3.35%-6.53%]	9.70% [6.78%-13.70%]	8.53% [7.03%-10.32%]	8.87% [6.58%-11.87%]	6.91% [5.31%-8.96%]	6.33% [4.49%-8.85%]
**Sensory or speech disorders: Blindness or vision problems**	4.59% [3.79%-5.55%]	2.47% [1.61%-3.77%]	4.33% [2.57%-7.21%]	3.59% [2.61%-4.93%]	6.03% [4.27%-8.45%]	3.16% [2.23%-4.47%]	19.96% [16.40%-24.07%]
**Mental or emotional disorders: Trauma and stressor-related disorders**	4.22% [3.23%-5.49%]	9.15% [6.81%-12.18%]	6.59% [3.20%-13.06%]	2.82% [1.90%-4.17%]	4.01% [2.54%-6.29%]	1.91% [1.08%-3.34%]	2.77% [1.31%-5.75%]
**Endocrine disorders: Diabetes**	4.13% [3.31%-5.14%]	3.93% [2.64%-5.80%]	6.18% [3.78%-9.95%]	5.81% [4.50%-7.46%]	6.44% [4.44%-9.27%]	2.71% [1.73%-4.23%]	4.82% [3.20%-7.21%]
**Other: Pain (unspecified)**	3.51% [2.67%-4.59%]	4.49% [3.18%-6.30%]	7.11% [4.36%-11.38%]	5.28% [3.79%-7.31%]	5.24% [3.46%-7.86%]	2.45% [1.41%-4.24%]	2.28% [1.31%-3.94%]
**Cardiovascular system disorders: High blood pressure**	3.41% [2.66%-4.37%]	2.45% [1.59%-3.77%]	6.11% [4.12%-8.97%]	4.46% [3.21%-6.18%]	5.09% [3.51%-7.34%]	2.22% [1.50%-3.28%]	3.98% [2.57%-6.12%]
**Cancer, tumor, cyst, or growth**	2.14% [1.62%-2.82%]	1.86% [1.15%-3.02%]	3.83% [1.99%-7.24%]	2.38% [1.66%-3.41%]	4.12% [2.62%-6.40%]	2.34% [1.59%-3.43%]	2.33% [1.32%-4.10%]

© 2026 Ne’eman A. *JAMA Health Forum*.

**Neurological disorders: Stroke or Brain Aneurysm**	2.14% [1.58%-2.90%]	3.90% [2.69%-5.63%]	5.57% [3.33%-9.18%]	3.27% [2.27%-4.70%]	4.24% [2.83%-6.32%]	1.62% [0.91%-2.88%]	2.54% [1.38%-4.63%]
**Musculoskeletal issues: Rheumatoid arthritis**	2.13% [1.49%-3.03%]	2.01% [0.98%-4.07%]	3.19% [1.42%-7.04%]	3.05% [2.11%-4.38%]	3.28% [1.99%-5.36%]	0.63% [0.24%-1.64%]	1.45% [0.67%-3.09%]
**Digestive system disorders (including liver conditions, stomach problems)**	2.02% [1.50%-2.71%]	2.70% [1.78%-4.06%]	2.18% [1.02%-4.60%]	2.89% [2.01%-4.14%]	3.23% [2.06%-5.03%]	1.51% [0.82%-2.79%]	2.39% [1.29%-4.40%]
**Immune system disorders**	1.98% [1.36%-2.87%]	2.86% [1.64%-4.92%]	1.68% [0.78%-3.58%]	2.28% [1.45%-3.56%]	2.86% [1.54%-5.26%]	1.74% [0.93%-3.24%]	2.33% [0.97%-5.50%]
**Mental or emotional disorders: Bipolar disorder**	1.88% [1.38%-2.55%]	4.09% [2.89%-5.77%]	1.51% [0.63%-3.57%]	1.24% [0.76%-2.00%]	2.13% [1.25%-3.61%]	0.95% [0.49%-1.86%]	0.69% [0.24%-1.96%]
**Other: Aging**	1.87% [1.41%-2.47%]	1.54% [0.93%-2.54%]	2.59% [1.30%-5.10%]	2.53% [1.73%-3.70%]	3.45% [2.29%-5.16%]	2.63% [1.76%-3.92%]	1.45% [0.71%-2.96%]
**Mental or emotional disorders: Any other or unspecified**	1.71% [1.19%-2.45%]	3.70% [2.52%-5.40%]	2.38% [0.91%-6.06%]	1.53% [0.88%-2.66%]	2.37% [1.25%-4.45%]	0.74% [0.26%-2.10%]	1.04% [0.46%-2.34%]
**Genitourinary disorders**	1.61% [1.11%-2.34%]	2.31% [1.32%-4.02%]	3.08% [1.54%-6.06%]	1.92% [1.25%-2.93%]	3.96% [2.33%-6.66%]	1.04% [0.58%-1.86%]	2.00% [0.98%-4.02%]
**Sensory or speech disorders: Any other or unspecified (including speech disorders, vestibular**	1.36% [0.97%-1.90%]	1.16% [0.63%-2.15%]	1.58% [0.69%-3.59%]	1.92% [1.30%-2.83%]	2.33% [1.37%-3.95%]	2.13% [1.29%-3.50%]	0.73% [0.35%-1.53%]

© 2026 Ne’eman A. *JAMA Health Forum*.

**problems)**							
**Neurological disorders: Dementia or Alzheimer's disease**	1.26% [0.94%-1.69%]	3.24% [2.40%-4.36%]	5.20% [3.44%-7.77%]	1.57% [1.05%-2.33%]	4.42% [3.19%-6.10%]	1.81% [1.18%-2.77%]	2.03% [1.18%-3.49%]
**Respiratory disorders: Asthma**	1.26% [0.86%-1.84%]	1.37% [0.71%-2.64%]	0.16% [0.02%-1.11%]	1.54% [0.93%-2.54%]	0.83% [0.36%-1.91%]	0.82% [0.42%-1.59%]	1.13% [0.41%-3.03%]
**Respiratory disorders: Chronic Obstructive Pulmonary Disease (COPD)**	1.18% [0.76%-1.84%]	0.87% [0.34%-2.15%]	1.75% [0.80%-3.76%]	1.62% [1.03%-2.54%]	3.60% [2.03%-6.28%]	1.23% [0.65%-2.32%]	1.48% [0.75%-2.91%]
**Respiratory disorders: Any other or unspecified**	1.05% [0.72%-1.51%]	0.93% [0.44%-1.94%]	2.33% [1.06%-5.05%]	1.80% [1.18%-2.75%]	1.92% [1.02%-3.61%]	0.66% [0.32%-1.33%]	1.56% [0.67%-3.57%]
**Neurodevelopmental or neurobehavioral disorders: Learning disability**	1.03% [0.63%-1.68%]	1.96% [1.09%-3.47%]	1.51% [0.45%-4.96%]	1.23% [0.63%-2.39%]	1.17% [0.44%-3.03%]	0.46% [0.17%-1.26%]	0.60% [0.19%-1.85%]
**Endocrine disorders: Any other or unspecified**	0.96% [0.63%-1.45%]	0.68% [0.31%-1.48%]	0.66% [0.16%-2.63%]	0.94% [0.51%-1.73%]	0.62% [0.23%-1.69%]	1.14% [0.58%-2.25%]	1.48% [0.70%-3.08%]
**Neurodevelopmental or neurobehavioral disorders: Autism Spectrum Disorder and Asperger Syndrome**	0.93% [0.60%-1.45%]	2.27% [1.43%-3.60%]	1.16% [0.45%-2.98%]	0.55% [0.25%-1.20%]	0.67% [0.26%-1.71%]	0.40% [0.12%-1.32%]	0.47% [0.12%-1.87%]

© 2026 Ne'eman A. *JAMA Health Forum*.

**Neurological disorders: Epilepsy or seizures**	0.89% [0.52%-1.52%]	2.01% [1.10%-3.63%]	1.02% [0.28%-3.65%]	0.91% [0.43%-1.91%]	2.63% [1.32%-5.16%]	0.15% [0.04%-0.61%]	0.36% [0.05%-2.54%]
**Other: COVID-19 or coronavirus**	0.45% [0.21%-0.96%]	0.83% [0.31%-2.16%]	0.70% [0.15%-3.08%]	0.65% [0.23%-1.83%]	1.07% [0.33%-3.44%]	0.00% [0.00%-0.00%]	0.34% [0.05%-2.39%]
**Neurodevelopmental or neurobehavioral disorders: Any other and unspecified**	0.28% [0.11%-0.72%]	0.28% [0.06%-1.16%]	0.72% [0.10%-4.92%]	0.31% [0.07%-1.35%]	0.40% [0.06%-2.82%]	0.12% [0.02%-0.87%]	0.20% [0.03%-1.44%]
**Neurodevelopmental or neurobehavioral disorders: Intellectual disability (due to a congenital disorder, such as Down Syndrome, or some other cause)**	0.17% [0.06%-0.48%]	0.20% [0.05%-0.83%]	0.58% [0.14%-2.39%]	0.32% [0.10%-1.03%]	0.20% [0.03%-1.41%]	0.33% [0.10%-1.04%]	0.15% [0.02%-1.07%]

All cells column percentages

© 2026 Ne’eman A. *JAMA Health Forum*.

Appendix Figure S1: Frequency of Reporting No Diagnosis, By Age Group

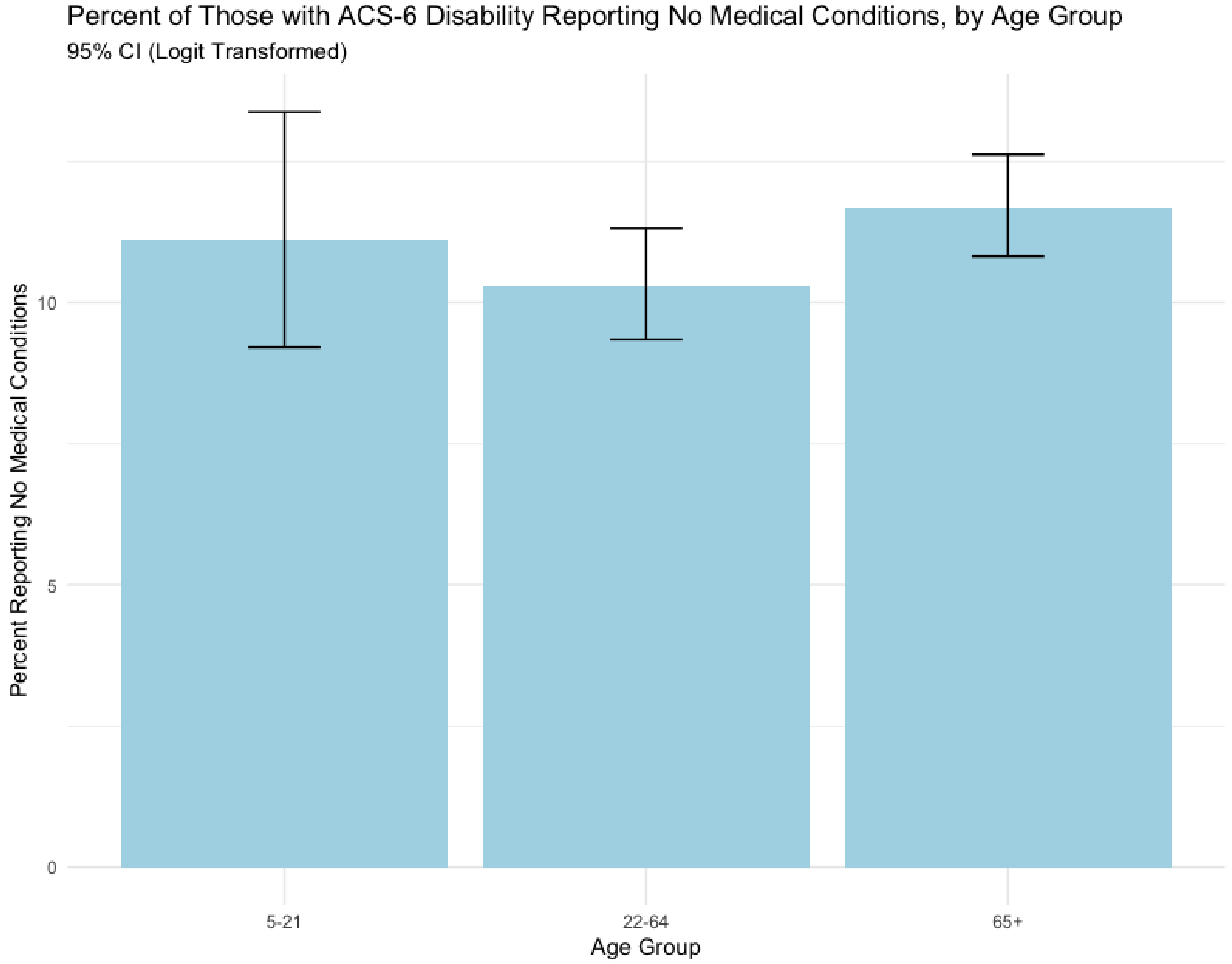

© 2026 Ne'eman A. *JAMA Health Forum*.

Appendix Figure S2: Frequency of Reporting No Diagnosis, By Age Group (2024 Data Only)

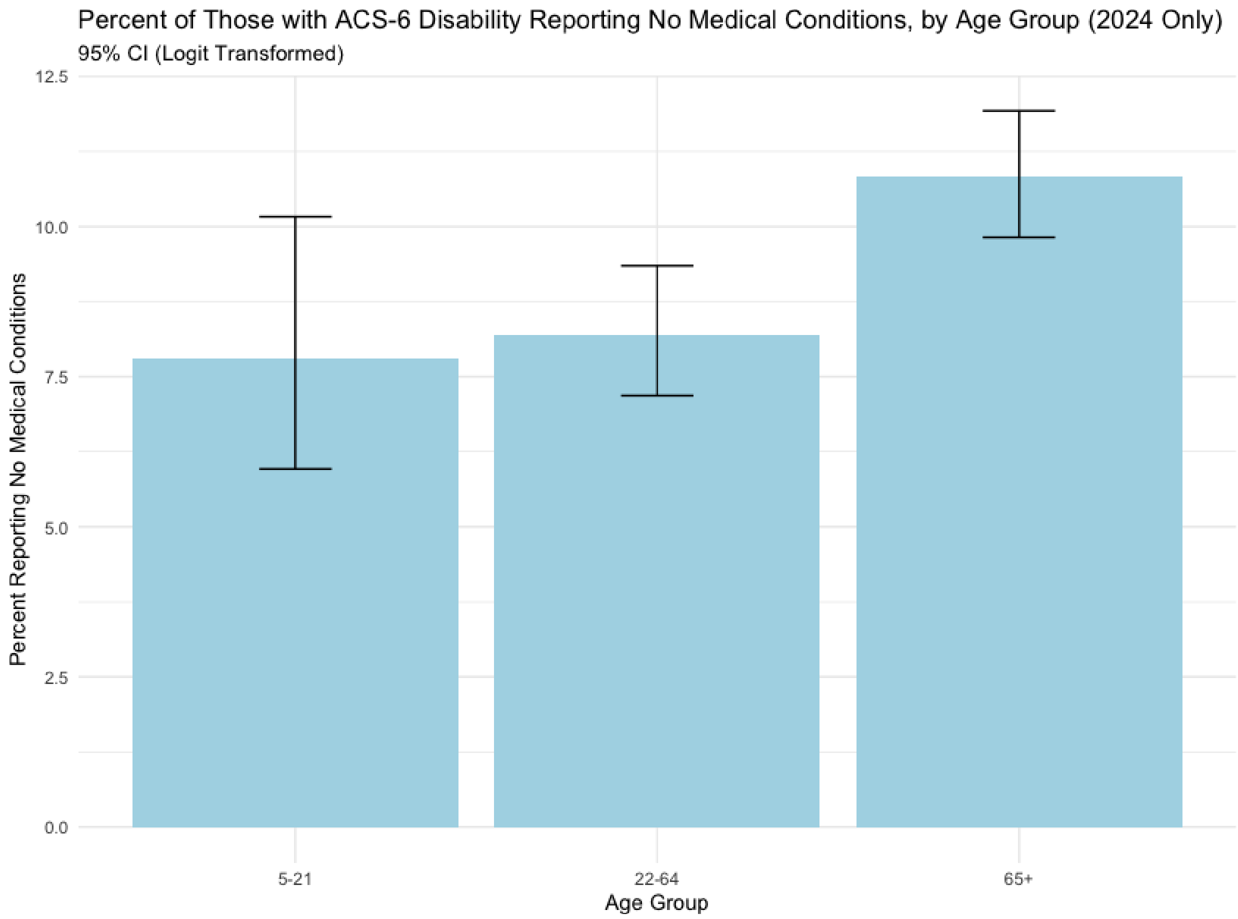

© 2026 Ne'eman A. *JAMA Health Forum*.

Appendix Figure S3: Change Over Time in Cognitive Disability Status Relative to 2019, 2018-2023

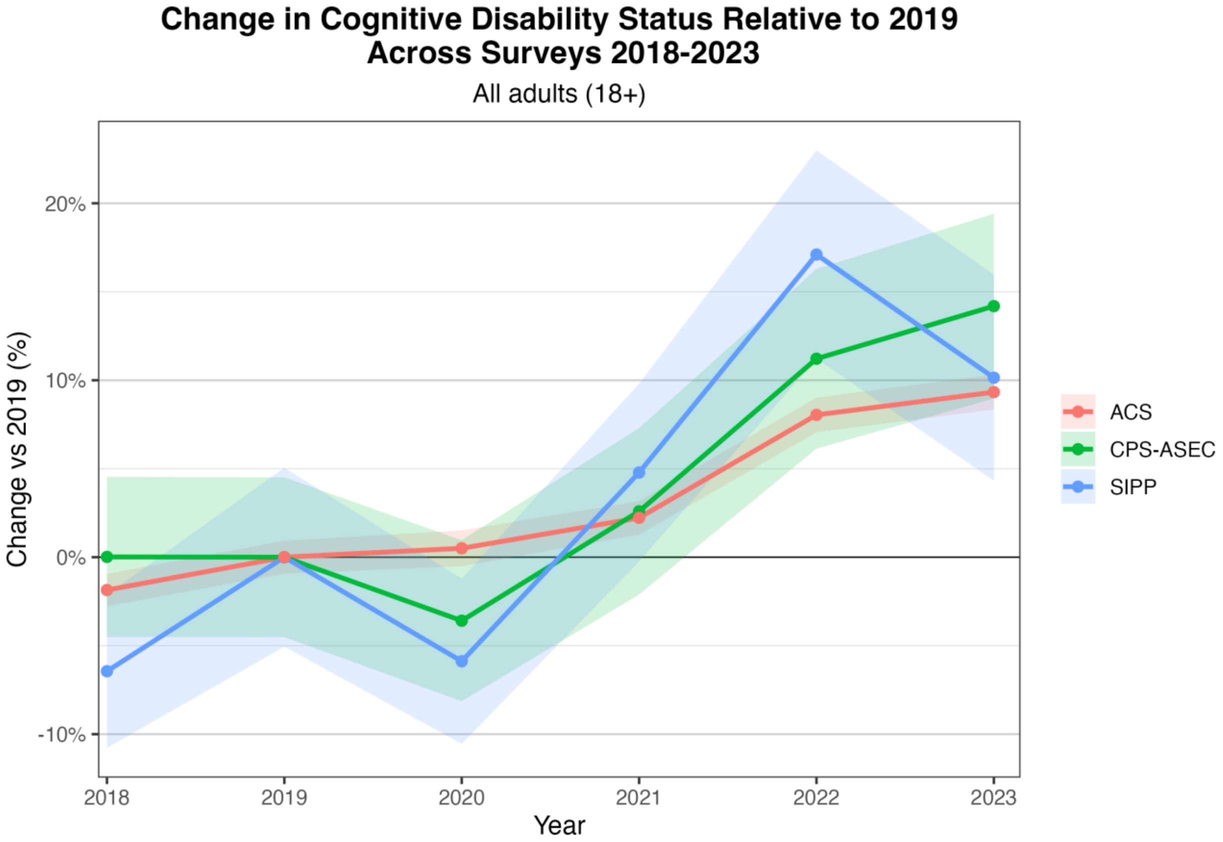

© 2026 Ne'eman A. *JAMA Health Forum*.
