# Supplementary material for: Disability Diagnoses Identified by the American Community Survey 6-Question Sequence
Source: JAMA Health Forum. 2026 Jan 23;7(1):e256302. doi: 10.1001/jamahealthforum.2025.6302 (PMC12831152; doi:10.1001/jamahealthforum.2025.6302)
Supplement: Supplement 2. — Data Sharing Statement [file jamahealthforum-e256302-s002.pdf]

Data Sharing Statement

Ne'eman. Disability Diagnoses Identified by the American Community Survey 6-Question Sequence. *JAMA Health Forum*. Published January 23, 2026.
doi:10.1001/jamahealthforum.2025.6302

Data
Data available: No

Additional Information
Explanation for why data not available: Data is already available in the Survey of Income and Program Participation public use files.
